# BiFeO_3_ Ceramics: Structure–Property Relationships and Doping Strategies

**DOI:** 10.3390/nano16191231

**Published:** 2026-09-30

**Authors:** Saba Aziz, Ritu Rawat, Silvia Rizzato, Angelo Leo, Gabriella Maria De Luca, Shahid Khalid, Sourav Kuila, Giuseppe Maruccio, Anna Grazia Monteduro

**Affiliations:** 1 Omnics Research Group, Department of Mathematics and Physics “Ennio De Giorgi”, University of Salento, Institute of Nanotechnology CNR-Nanotec, INFN Sezione di Lecce, Via per Monteroni, 73100 Lecce, Italy; saba.aziz@unisalento.it (S.A.); ritu.rawat@unisalento.it (R.R.); angelo.leo@unisalento.it (A.L.); souravbypass@gmail.com (S.K.); annagrazia.monteduro@unisalento.it (A.G.M.); 2 Dipartimento di Fisica “E. Pancini”, University of Naples “Federico II”, Complesso Monte Sant’Angelo, Via Cinthia, 80126 Napoli, Italy; gabriellamaria.deluca@unina.it; 3 Department of Materials Science, University of Milano Bicocca, 20126 Milan, Italy; shahid.khalid@unimib.it

**Keywords:** BiFeO_3_, multiferroics, ceramics, structure–property relationships, magnetoelectric coupling, doping, dielectric, ferroelectric, antiferromagnetism

## Abstract

Bismuth ferrite (BiFeO_3_, BFO) is a lead-free perovskite that combines robust ferroelectricity and antiferromagnetism at room temperature, making it one of the most extensively studied room-temperature multiferroics for spintronics, sensors, and multifunctional devices. In bulk ceramics, however, Bi volatility, oxygen-vacancy-driven leakage, and parasitic phases complicate synthesis and obscure intrinsic ferroic response. This review critically revisits the fundamental physical properties of bulk BiFeO_3_ ceramics by elucidating the relationships between crystal structure, defect chemistry, processing, and ferroic functionalities. Particular emphasis is placed on the evolution of the understanding of the intrinsic ferroelectric, magnetic, dielectric, and magnetoelectric properties of bulk BiFeO_3_, highlighting the landmark experimental and theoretical studies that have shaped the current picture of this material. The review further examines A-site (Bi) and B-site (Fe) substitutions and selected co-doping strategies, assessing how they modify structural stability and ferroic behavior. Finally, representative performance benchmarks for undoped and doped bulk ceramics are compared, and the remaining challenges toward achieving reproducible room-temperature magnetoelectric functionality are critically discussed. Overall, this review provides a unified framework linking crystal chemistry, processing methods, microstructure, doping engineering, and ferroic functionalities, offering practical guidelines for the rational design of high-performance BiFeO_3_-based bulk ceramics for next-generation multifunctional devices.

## 1. Introduction

Advances in functional materials strengthen modern technologies—from information and communication technology (ICT) and power electronics to advanced manufacturing and medical devices—delivering improvements in efficiency, functionality, and quality of life. A key area of ongoing research is the development of multifunctional materials that integrate multiple coexisting order parameters within a single phase, enabling further miniaturization and low-power operation, while expanding the range of achievable device functionalities. In this context, multiferroic materials combine ferroelectric and magnetic order, allowing information to be encoded in ferroic domains and written or read using either electric or magnetic fields, thereby enabling cross-field control for non-volatile, energy-efficient memory and hybrid devices [1,2,3,4].

Several classes of single-phase multiferroics have been extensively investigated, including BiMnO_3_, hexagonal manganites such as YMnO_3_, and spin-driven Type-II systems, such as TbMnO_3_ and Ni_3_V_2_O_8_, in which ferroelectric polarization emerges as a consequence of magnetic ordering. These materials exhibit different microscopic origins of ferroelectricity and magnetoelectric coupling, as discussed in detail in Section 2.2. However, in many of these systems, the coexistence of magnetic and ferroelectric order is restricted to temperatures well below room temperature, while spin-driven multiferroics generally exhibit relatively small spontaneous polarization. Among single-phase multiferroics, bismuth ferrite (BiFeO_3_, BFO) occupies a distinctive position because it combines robust ferroelectricity and G-type antiferromagnetic order at room temperature.

The antiferromagnetic order sets in below the Néel temperature (T_N_ ≈ 643 K), whereas ferroelectricity persists below the Curie temperature (T_C_ ≈ 1103 K) [5]. In bulk ceramics, however, defect chemistry—particularly oxygen vacancies and mixed Fe^2+^/Fe^3+^ valence states—strongly affects charge transport and dielectric response, often obscuring the intrinsic functional properties of BFO [6]. A particularly attractive feature of BFO is the coupling between its ferroelectric polarization and antiferromagnetic order through the magnetoelectric (ME) effect, enabling electric-field manipulation of magnetic states at room temperature. This electrical control of antiferromagnetic domains has been demonstrated in specific architectures, establishing a practical magnetoelectric (ME) pathway relevant to memory and sensing [7,8,9,10]. These results have driven broad theoretical and experimental studies of ME coupling across BFO forms and compositions [11,12].

BFO has been extensively investigated in different forms—including bulk ceramics, single crystals, thin films, and nanostructures—each offering distinct advantages and limitations [13,14,15]. Several review articles have summarized these developments from a complementary perspective. Rojac et al. [6] reviewed the processing, electrical, and electromechanical properties of BFO ceramics, highlighting the strong influence of processing and microstructure on the properties of bulk materials. Yang et al. [9] focused on BFO thin films, with particular emphasis on electric-field control of their multifunctional properties, while Liu et al. [8] discussed electrical, magnetoelectric, and optical functionalities through domain engineering. Wu et al. [16] provided a broader overview of BFO-based materials spanning bulk ceramics, thin films, and nanostructures. More recent reviews have further addressed magnetoelectric coupling, BFO-based lead-free multifunctional materials, and the enhancement of multiferroic properties through chemical substitution and structural phase transitions [13,17,18].

Among the different forms of BFO, bulk ceramics are particularly relevant for investigating processing-dependent structure–property relationships in polycrystalline materials without the additional influence of epitaxial constraints and, at the same time, represent a potentially scalable platform for multifunctional applications. Their preparation, however, remains particularly challenging because high-temperature processing can promote Bi volatility, secondary-phase formation, oxygen non-stoichiometry, and associated electrical leakage. Understanding how these interconnected factors govern the functional response is therefore essential both for identifying the intrinsic behavior of bulk BFO and for developing effective compositional and processing strategies.

The present review addresses this need by examining the literature on bulk BFO ceramics and systematically compiling the main results obtained through chemical substitution at the A and B sites and through co-doping strategies. Attention is given to the structural and functional properties of the investigated compositions in relation to their synthesis routes and microstructural characteristics, allowing the effects of chemical substitution to be considered together with processing- and microstructure-related contributions. To provide a broader perspective on the evolution of BFO properties, selected results obtained for undoped and doped BFO nanoparticles are also included, enabling comparison between bulk ceramics and nanoscale systems and providing further insight into the combined effects of chemical substitution and nanostructuring. Within this framework, the review aims to establish clearer structure–processing–defect–property relationships and to identify the main factors governing the functional response of BFO-based materials. Finally, key challenges and future research directions are critically discussed.

## 2. Fundamentals in Multiferroics

Multiferroic materials combine multiple ferroic order parameters within a single material, either intrinsically in a single phase or through interfacial coupling in artificially engineered heterostructures. Their unique appeal lies in the possibility of coupling these order parameters, allowing one ferroic state to be controlled through the external field conjugated to another. This concept extends beyond conventional ferroic materials, where coupling between an order parameter and its conjugate field gives rise to functional responses such as piezoelectricity, magnetostriction, and, in multiferroics, magnetoelectric coupling. The term ferroic encompasses the ferroelectric, ferromagnetic (including ferrimagnetic and antiferromagnetic), ferroelastic, and ferrotoroidic states [19,20]. Each ferroic order is characterized by a spontaneous order parameter that can be switched by its corresponding conjugate external field.

**Figure 1:**
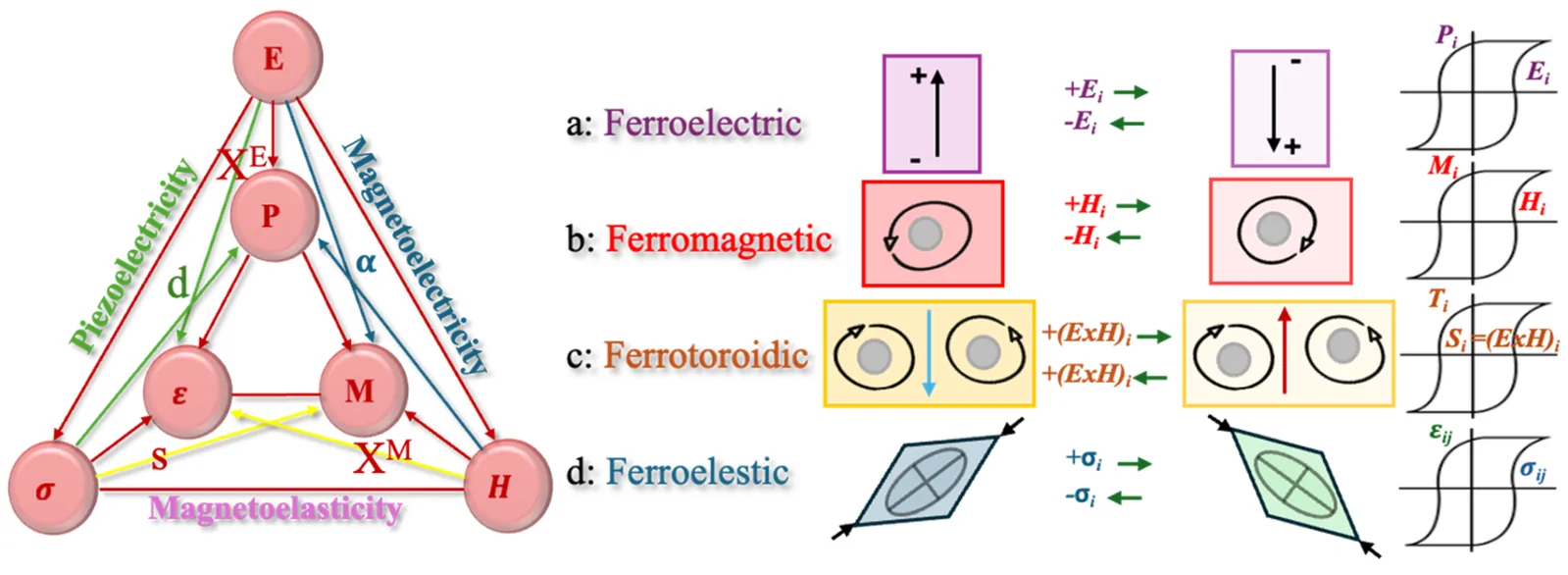
(**left**) The three most common ferroic orders (ferroelectric, ferromagnetic, and ferroelastic) and their coupling via magnetoelectricity, piezoelectricity, and magnetoelasticity. (**right**) (a–d) The main ferroics (including also ferrotoroidic) with schematics for their ordering parameters, direct control fields, and hysteresis curves. The symmetry symbols that are used for the toroidal moments are related to the coaxial mirror plane cross-section of toroid cores with an even number of turns (windings) [20].

Figure 1 schematically illustrates the three primary ferroic orders in multiferroic materials—ferroelectricity, ferromagnetism, and ferroelasticity—together with their corresponding order parameters, conjugate external driving fields, and the principal coupling mechanisms connecting them. The interplay between these ferroic orders gives rise to a variety of cross-coupled physical phenomena that are at the origin of the multifunctional behavior of multiferroics. Among these coupling mechanisms, magnetoelectric (ME) coupling is particularly important because it allows the magnetic state to be manipulated by an electric field and, conversely, the electric polarization to be controlled by a magnetic field [1,2,3]. This bidirectional coupling forms the basis for numerous applications, including low-power non-volatile memory, sensors, actuators, and emerging spintronic and multifunctional electronic devices.

The coexistence and coupling of multiple ferroic orders within a single material arise from specific symmetry-breaking mechanisms in the crystal structure. As a result, ferroic order parameters that would otherwise be symmetry-forbidden can coexist and interact within the same phase. In particular, the reduction in crystal symmetry enables the simultaneous emergence of ferroic properties that are generally incompatible in higher-symmetry structures [20].

### 2.1. Magnetoelectric Coupling in Multiferroics

Among the various coupling phenomena occurring in multiferroics, magnetoelectric (ME) coupling is arguably the most technologically relevant because it enables direct interaction between electric polarization and magnetic order. The possibility of controlling magnetic order through an electric field provides a promising strategy for the development of ultra-low-power spintronic devices. It is also highly relevant for magnonics, where information is carried by spin waves rather than charge currents, thereby enabling low-power signal processing [21].

One of the most promising applications of ME coupling is magnetoelectric random-access memory (MERAM) [22], together with advanced magnetic sensors and reconfigurable spintronic devices [23]. Unlike conventional Magnetic Random Access Memory (MRAM) technologies, which rely on current-induced switching, MERAM combines the advantages of FeRAM (Ferroelectric Random Access Memory) and MRAM by exploiting electric-field control of magnetic order, significantly reducing write energy through modulation of the interfacial exchange coupling between a multiferroic material and an adjacent ferromagnet [22].

Beyond its technological impact, ME coupling represents a unique physical platform for investigating the interplay between spin, charge, lattice, and orbital degrees of freedom in strongly correlated materials. Understanding these interactions is essential for the rational design of next-generation multifunctional materials with enhanced magnetoelectric responses.

The scientific basis of ME coupling is rooted in crystal symmetry and energy minimization principles. Within this framework, Landau theory provides a phenomenological description of the magnetoelectric effect by expressing the free energy (F) of the system as a function of the electric (E) and magnetic (H) fields. Magnetoelectric coupling arises from symmetry-allowed terms in the free-energy expansion, with the lowest-order interaction described by the linear magnetoelectric tensor (α) [24,25]. This framework establishes the symmetry conditions for ME coupling and provides the thermodynamic basis for cross-field control of electric and magnetic order parameters. Using Einstein summation notation for an infinite, homogeneous, and stress-free medium, the free energy can be expressed as follows:

(1) $$\begin{array}{cc} F\left(E,H\right)= & F_{0}-P_{i}^{s}E_{i}-M_{i}^{s}H_{i}-\frac{1}{2}\varepsilon_{0}\varepsilon_{\mathrm{ij}}E_{i}E_{j}-\frac{1}{2}\mu_{0}\mu_{\mathrm{ij}}H_{i}H_{j}-\alpha_{\mathrm{ij}}E_{i}H_{j}-\frac{1}{2}\beta_{\mathrm{ijk}}E_{i}H_{j}H_{k}\\ & -\frac{1}{2}\gamma_{\mathrm{ijk}}H_{i}E_{j}E_{k}+\cdots . \end{array}$$

In Equation (1), the electric (E) and magnetic (H) fields are expressed through their Cartesian components, while ε*_ij_* and μ*_ij_* denote the dielectric permittivity and magnetic permeability tensors, respectively. The first term F_0_ represents the free energy density of the system in the absence of external electric and magnetic fields. In non-ferroic materials, both spontaneous polarization and spontaneous magnetization vanish in the absence of external fields, and consequently no ferroic hysteresis is observed. By contrast, in ferroic materials the second and third terms account for the contributions associated with the spontaneous polarization (P is) and spontaneous magnetization (M is), respectively. The fourth and fifth terms describe the linear dielectric and magnetic responses of the material to externally applied electric and magnetic fields, respectively, whereas the sixth term represents the linear magnetoelectric interaction through the second-rank tensor α*_ij_*_._ The third-rank tensors β*_ijk_* and γ*_ijk_* account for higher-order magnetoelectric interactions [3].

Within this framework, the magnetoelectric response can be described in terms of the electric polarization *P_i_* and magnetization *M_i_*, both of which depend on the electric and magnetic fields. The constitutive relations for polarization *P_i_* and magnetization *M_i_* are obtained by differentiating the free-energy expansion with respect to their conjugate fields [2,3]:

(2) $$P_{i}\left(E,H\right)=-\frac{\partial F}{\partial E_{i}}{=P}_{i}^{s}\;+\varepsilon_{0}\varepsilon_{\mathrm{ij}}E_{j}+\;\alpha_{\mathrm{ij}}H_{j}+\frac{1}{2}\beta_{\mathrm{ijk}}H_{j}H_{k}+\gamma_{\mathrm{jik}}H_{j}E_{k}-\cdots$$

whereas the corresponding constitutive relation for magnetization is given by

(3) MiE, H=−∂F∂Hi=M is+μ0μijHj+αjiEj+βjikEjHk+12γijkEjEk+⋯

Equations (2) and (3) show that both the electric polarization and the magnetization depend not only on their respective conjugate fields but also on the complementary field through the magnetoelectric coupling terms, highlighting the intrinsic coupling between the electric and magnetic degrees of freedom. Therefore, a non-zero linear magnetoelectric coefficient α*_ij_* implies that an applied magnetic field can induce electric polarization, while an applied electric field can induce magnetization. When the tensors β*_ijk_* and γ*_ijk_* are non-zero, higher-order magnetoelectric contributions also arise. For the linear magnetoelectric response, thermodynamic stability imposes an upper bound on the magnitude of the magnetoelectric coefficient, relating it to the dielectric permittivity and magnetic permeability of the material [3]:

(4) $$\alpha_{\mathrm{ij}}^{2}\;\leq \varepsilon_{0}\mu_{0}\varepsilon_{\mathrm{ii}}\mu_{\mathrm{jj}}$$

Equation (4) is obtained within the linear approximation of the free-energy expansion, where higher-order magnetoelectric coupling terms are neglected. It establishes an upper bound for the linear magnetoelectric coefficient, with the maximum allowed value determined by the geometric mean of the corresponding diagonal components of the dielectric permittivity and magnetic permeability tensors. Accordingly, large dielectric permittivity and magnetic permeability increase the thermodynamically allowed upper bound for, but do not necessarily lead to, a large magnetoelectric response. The actual magnitude of the magnetoelectric response ultimately depends on the effectiveness of the microscopic mechanisms coupling the electric and magnetic degrees of freedom.

### 2.2. Multiferroic Materials—A Journey

The experimental realization of magnetoelectric coupling has been the subject of extensive research for several decades. Debye [26] first introduced the term “magnetoelectric” in 1926 to describe the coupling between electric and magnetic properties. For the antiferromagnetic Cr_2_O_3_, Dzyaloshinskii [27] predicted the existence of the linear magnetoelectric effect on the basis of symmetry considerations, providing the first theoretical framework for magnetoelectric coupling. This prediction was experimentally confirmed shortly afterwards by Astrov [28], who demonstrated electric-field-induced magnetization, followed by complementary observations of magnetic-field-induced polarization by Rado and Folen [29,30]. These pioneering discoveries stimulated the search for single-phase materials combining magnetic and ferroelectric order. Early efforts, beginning in the 1950s, explored complex perovskite oxides combining ferroelectric and magnetic constituents [31]. Although these early studies established the foundations of multiferroic materials research, a major turning point occurred in 2003 with the landmark studies on TbMnO_3_ and BiFeO_3_ [32,33], which stimulated renewed interest in the microscopic mechanisms of multiferroicity and magnetoelectric coupling. Subsequent studies further demonstrated remarkable cross-coupled phenomena, including magnetic-field-induced polarization reversal and magnetically driven ferroelectric order [1,34,35].

The wide variety of multiferroic materials investigated over the past decades can be classified according to the microscopic origin of ferroelectricity and its relationship with magnetic ordering. Following the classification proposed by Khomskii [36], multiferroics are broadly divided into two principal classes, referred to as **Type-I** and **Type-II** systems. This distinction is particularly relevant because the microscopic origin of ferroelectricity and its relationship with magnetic order strongly influence the magnitude of the spontaneous polarization, the characteristic ordering temperatures, and the strength of the magnetoelectric coupling. As summarized in Table 1, **Type-I** multiferroics include materials in which ferroelectricity originates from lone-pair activity, charge ordering, or geometric distortions. In these materials, ferroelectricity and magnetism originate from different microscopic mechanisms, and coupling between the two order parameters may still produce a magnetoelectric response. Ferroelectric ordering generally occurs at considerably higher temperatures than magnetic ordering, while the spontaneous polarization is often relatively large compared with that of Type-II multiferroics. For example, **YMnO_3_** exhibits a spontaneous polarization of approximately 6 µC cm^−2^ (*T_N_* = 75–76 K and *T_FE_* ∼ 914 K) [37], whereas **BiFeO_3_** exhibits experimentally measured polarization values of approximately 60 μC cm^−2^ (*T_N_* = 643 K, *T_FE_* ≈ 1100 K) in high-quality samples [15]. In BiFeO_3_, the Fe^3+^ 3d electrons are responsible for the canted G-type antiferromagnetic order, while the stereochemically active Bi^3+^ 6s^2^ lone pair drives the ferroelectric distortion [36,38]. This design strategy has enabled the development of numerous Bi-based Type-I multiferroics [39]. A different route to coupling magnetic and polar degrees of freedom is provided by **EuTiO_3_**, where epitaxial strain can induce a ferroelectric distortion coupled to the Eu^2+^ magnetic moments [40]. Fluorides, **BaMF_4_** (M = Mn, Ni, Co, Fe, and Cu), represent another important family of Type-I multiferroics that were discovered several decades ago but have attracted renewed attention in recent years [41,42,43]. In such materials, the collective rotation of MF_6_ octahedra is thought to be the source of ferroelectricity, while transition metals are responsible for magnetism. Some fluorides have ferroelectric Curie temperatures that can even approach 1000 K. The AFM ordering, however, normally appears at a considerably lower temperature, often T_N_ ~ 50 K. Another representative family of Type-I multiferroics is the hexagonal manganites **h-RMnO_3_** (R = rare-earth element, Y, Ho, Dy, In, etc.). Interestingly, chemical substitution and strain engineering can transform the hexagonal phase into the orthorhombic structure, resulting in completely different multiferroic behavior and magnetoelectric coupling (Table 1) [33,44,45]. Closely related hexagonal ferrites (**h-RFeO_3_**) also exhibit geometrically driven multiferroicity [46,47,48]. Compared with h-RMnO_3_, hexagonal RFeO_3_ is thermodynamically less stable than its orthorhombic counterpart but generally exhibits a significantly higher Néel temperature, making it particularly attractive for multiferroic applications [49]. Unlike BiFeO_3_, switchable ferroelectric photovoltaic effects have recently been demonstrated in h-RFeO_3_ [50].

**Type-II** multiferroics differ fundamentally from Type-I materials because the ferroelectric polarization is directly induced by magnetic ordering. Depending on the microscopic mechanism responsible for polarization, Type-II multiferroics can be broadly divided into two categories: (i) materials in which ferroelectricity is induced by spiral magnetic order and (ii) materials in which ferroelectricity arises within collinear magnetic structures. Numerous Type-II multiferroics have been reported in recent years, spanning **transition-metal oxides** and **chalcogenides** (Table 1). Representative examples include **(Ni, Co)_2_V_2_O_7_** [51], **LiFe(WO_4_)_2_** [52], **α-NaFeO_2_** [53], **Ni_3_V_2_O_8_** [54], **RFeWO_6_** [55], **(La, Bi)Mn_3_ Cr_4_ O_12_** [56,57], **Mn_2_O_3_** [58], **Ni_3_TeO_6_** [59,60], **Co_4_Nb_2_O_9_** [61], **(Fe, Mn)_2_Mo_3_O_8_** [62,63], **Mn_2_MnWO_6_** [64], I**n_2_NiMnO_6_** [65], **NaFeSi_2_O_6_** [66]. Beyond oxides, compounds containing other anions, such as sulfur and selenium, also exhibit Type-II multiferroicity, including **BaFe_2_Se_3_** [67], **CaOFeS** [68], **MnSb_2_S_4_** [69], **CsCuCl_3_** [70], **Cu_3_Bi(SeO_3_)_2_O_2_Cl** [71], and **CuBr_2_** [72]. The number of spin-driven multiferroics continues to grow, and materials exhibiting improved electric and magnetic responses at or near room temperature are actively being explored. Nevertheless, two major challenges remain. First, most spin-driven and collinear-magnetism-induced multiferroics exhibit multiferroicity only at temperatures well below room temperature. Second, the spontaneous polarization generated through magnetic ordering is generally much smaller than that found in conventional ferroelectrics [3,36]. Despite the stringent symmetry requirements governing multiferroicity, simultaneous ferroelectric and magnetic ordering have been achieved in only a limited number of single-phase materials, especially oxide multiferroics containing transition-metal cations, such as hexagonal **YMnO_3_** and perovskite **BiFeO_3_** and **BiMnO_3_** [12,73,74,75]. Among these compounds, BiFeO_3_ occupies a unique position owing to the coexistence of robust ferroelectric and antiferromagnetic order well above room temperature, making it the benchmark single-phase multiferroic for both fundamental studies and technological development [12]. Together with its lone-pair-driven ferroelectricity and lead-free composition, these high ordering temperatures make BFO particularly attractive for non-volatile memories, sensors, actuators, and other multifunctional electronic devices [12,14]. Furthermore, concerns regarding the toxicity of lead and regulatory restrictions on lead-containing materials have stimulated the development of lead-free alternatives to conventional lead-based piezoelectric materials such as Pb(Zr_x_Ti_1−x_)O_3_ (PZT) [76,77]. Consequently, the development of lead-free functional materials has become an important research direction for applications including sensors, actuators, and transducers. Although PZT remains the industrial benchmark and is still permitted under specific exemptions, the development of sustainable lead-free alternatives has become a major research priority [77].

**Table 1:** Classification of Type-I and Type-II multiferroics according to the microscopic origins of ferroelectricity and magnetism. Representative compounds, characteristic properties, strengths, limitations, and major research and application areas are summarized. BiFeO_3_ is underlined as the benchmark room-temperature single-phase multiferroic and the primary focus of this review.

Class	Origin of Ferroic Orders	Compounds	Typical Properties	Strengths	Limitations	Main Research/Application Interest
**Type-I** Lone pair driven	**FE:** Off-centering induced by stereochemically active *ns*^2^ lone pairs (Bi^3+^, Pb^2+^) [36,39] **Magnetism:** Independent ordering of transition-metal ions (e.g., Fe^3+^, Mn^3+^), resulting in weak intrinsic ME coupling	**BiFeO_3_**, BiMnO_3_	T_FE_: 600–1100 K; T_N_: up to ~650 K; P: up to 100 μC cm^−2^. **BiFeO_3_**: T_FE_ = 1103 K, T_N_ = 643 K, P_s_ ≈ 60 μC cm^−2^ [78,79]	Large spontaneous polarization High ordering temperatures Stable RT multiferroicity	FE and magnetism originate from different microscopic mechanisms, resulting in weak intrinsic ME coupling. Leakage current and secondary phases (especially in BiFeO3)	Non-volatile memories spintronics ME sensors actuators multifunctional devices
**Type-I** Charge-order (CO)-driven	**FE:** Non-centrosymmetric ordering of mixed-valence ions [6] **Magnetism:** Independent magnetic ordering with weak intrinsic ME coupling.	LuFe_2_O_4_, Fe_3_O_4_, (Pr,Ca)MnO_3_	Moderate polarization; charge ordering generally occurs near or below RT. **LuFe_2_O_4_**: T_CO_ ≈ 330 K, T_N_ ≈ 240 K [20].	Purely electronic ferroelectricity Fast electronic response	Charge-order mechanism remains debated Limited number of established compounds [6,9]	Fundamental studies of correlated-electron systems and electronic ferroelectricity
**Type-I** Geometric ferroelectricity	**FE:** Collective structural distortions (tilting/rotation of polyhedra) [37] **Magnetism:** Independent ordering of transition-metal magnetic moments with weak intrinsic ME coupling.	h-RMnO_3_, h-RFeO_3_, BaNiF_4_	T_FE_ up to ~1000–1200 K. T_N_ ≈ 50–150 K; P < 10 μC cm^−2^. **h-YMnO_3_**: P ≈ 6.5 μC cm^−2^ [37]; **BaNiF_4_**: P ≈ 0.01 μC cm^−2^ [41]	Robust structural origin of ferroelectricity excellent thermal stability	Weak intrinsic ME coupling Moderate polarization (Additional theoretical and experimental studies are needed to fully understand polarization mechanisms)	Improper ferroelectricity domain-wall engineering topological vortex structures
**Type-II** Spin-spiral driven	**Magnetism:** Inverse DM interaction induced by non-collinear spin spirals [7,48] Spiral magnetic ordering directly induces **FE**, giving strong intrinsic ME coupling.	TbMnO_3_, DyMnO_3_, Ni_3_V_2_O_8_, CuBr_2_	P ≈ 10^−3^–1 μC cm^−2^; magnetic ordering typically below 50 K. **TbMnO_3_**: T_N1_ ≈ 41 K, T_N2_ ≈ 28 K [34]	Very strong intrinsic ME coupling and electric-field control of magnetic textures.	Very low operating temperatures Small spontaneous polarization	Magnetoelectric physics spintronics electric-field control of magnetic textures
**Type-II** Collinear spin-order driven	**FE:** Exchange striction associated with collinear magnetic structures [36]. **Magnetism:** Collinear magnetic ordering directly induces ferroelectricity, resulting in strong intrinsic ME coupling.	o-HoMnO_3_, Ca_3_CoMnO_6_, In_2_NiMnO_6_, Co_4_Nb_2_O_9_, RMn_2_O_5_ (R = Y, Dy, Tb, Gd)	P up to ~1 μC cm^−2^; ordering generally below 100 K. **GdMn_2_O_5_**: ΔPb ≈ 0.5 μC cm^−2^ at 2 K under 4.7 T [80].	Strong ME coupling and larger polarization than spin-spiral systems.	Most compounds operate only at cryogenic temperatures, limiting practical applications.	Magnetoelectric switching spintronics lectrically controlled magnetic devices

## 3. Bismuth Ferrite (BFO) Multiferroic Ceramics

Among the different forms of BFO, bulk ceramics are particularly relevant for investigating fundamental structure–property relationships while also providing a scalable platform for multifunctional devices. However, achieving high-performance bulk BFO remains considerably more challenging than fabricating thin films or single crystals. Although thin films often exhibit enhanced polarization and switching characteristics due to epitaxial strain and substrate-induced effects, bulk ceramics provide a practically scalable platform for studying structure–property relationships in polycrystalline BFO [81,82,83].

The preparation of single-phase BFO ceramics is, however, complicated by the volatility of Bi^3+^ during high-temperature processing, which is responsible for high leakage current in BFO ceramics, the narrow temperature range for phase stabilization, and the formation of secondary phases such as Bi_25_FeO_39_, Bi_2_Fe_4_O_9_, and Bi_2_O_3_ [79,84]. In addition, oxygen vacancies and mixed-valence iron ions (Fe^3+^/Fe^2+^) can substantially increase electrical conductivity, resulting in high leakage current, degraded dielectric and ferroelectric properties, and difficulty in achieving complete electrical poling [84,85,86]. Secondary phases and defect-mediated mechanisms can also affect the measured magnetic response. These processing- and defect-related challenges have motivated extensive research aimed at optimizing synthesis routes, controlling defect chemistry, and tailoring the structural and ferroic properties of BFO through compositional and microstructural engineering.

To improve functional properties, different ceramic processing strategies have been explored, including conventional solid-state reaction, microwave-assisted sintering, rapid liquid-phase sintering, ball-milling-assisted routes, spark plasma sintering, and two-step sintering. Indeed, the processing conditions can strongly affect phase formation, density, grain size, porosity, defect concentration, and, consequently, the resulting electrical and magnetic response. Alongside processing optimization, chemical substitution at the A site, B site, or simultaneously at both sites represents one of the most widely investigated strategies for controlling phase stability, defect chemistry, and the functional properties of BFO.

In this review, the principal results reported for A-site-, B-site-, and co-doped BFO ceramics are systematically compared, together with the available information on synthesis routes and microstructural characteristics. This approach allows the reported functional properties to be considered in relation not only to dopant chemistry and concentration but also to processing- and microstructure-dependent effects. Nanostructuring provides an additional route for modifying the properties of BFO.

BFO nanoparticles have been synthesized through several chemical routes, including sol–gel, co-precipitation, and hydrothermal methods, in which parameters such as temperature, precursor chemistry, solvent, and pressure can be used to control particle size, morphology, and crystallinity [87,88]. Size reduction can also strongly affect the magnetic response; in particular, dimensions approaching or below the characteristic spin-cycloid period (~62 nm) can contribute to enhanced magnetization through suppression or modification of the cycloidal spin structure. Selected results for undoped and doped BFO nanoparticles are therefore also considered in this review to provide a broader perspective on the effects of chemical substitution and nanostructuring on BFO properties and to facilitate comparison with corresponding bulk ceramic systems.

### 3.1. Crystal Structure of BFO Ceramics

**Figure 2:**
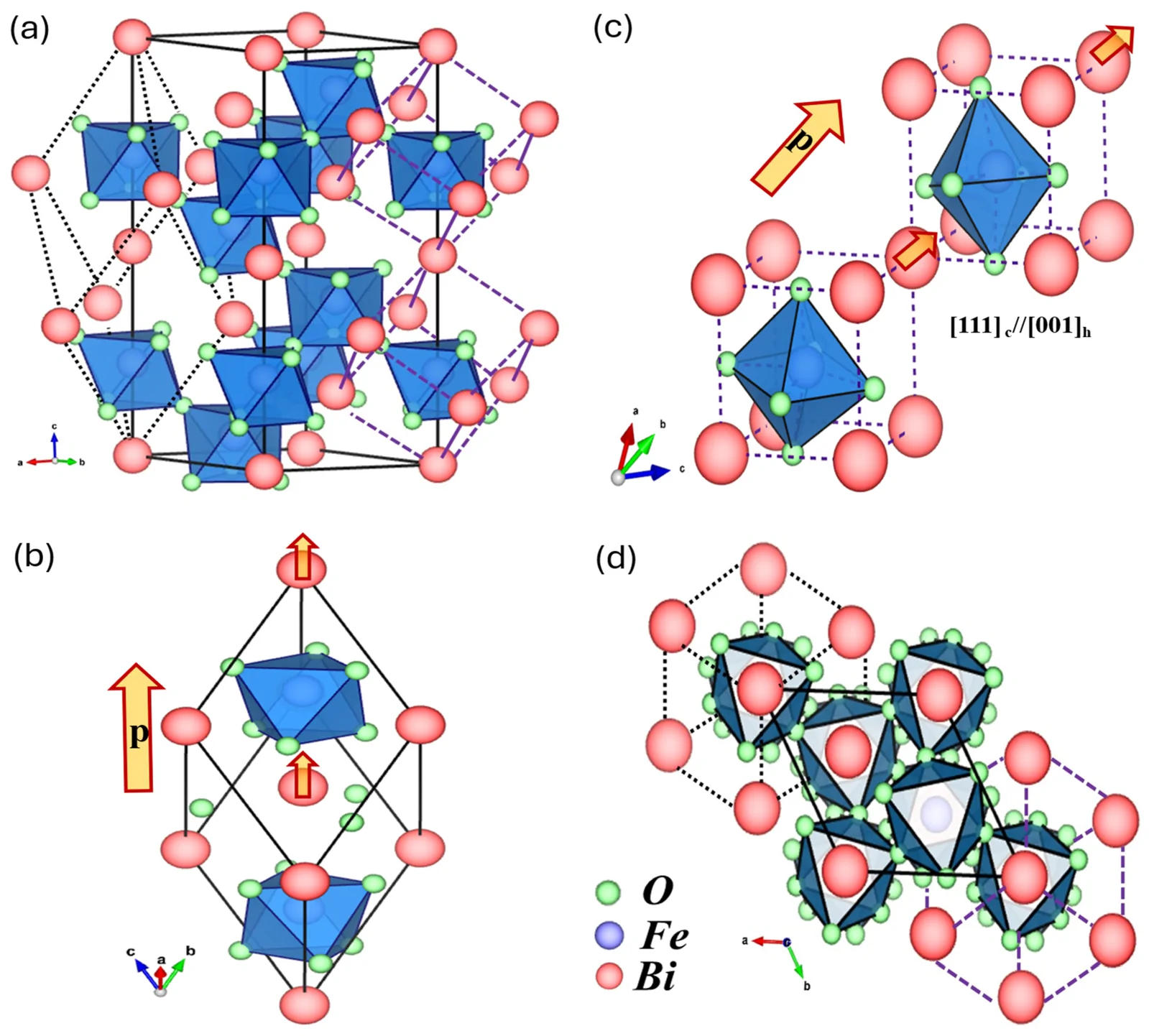
(**a**–**d**) Crystallographic representations of the BiFeO_3_ structure. (**a**) The hexagonal unit cell contains six formula units. (**b**) The rhombohedral unit cell contains two formula units. (**c**) The pseudocubic unit cell contains one formula unit. The [111] direction in the rhombohedral and the pseudocubic notation corresponds to the [001] direction in the hexagonal notation. Yellow and blue arrows indicate the displacements of the Bi^3+^ and Fe^3+^ cations, respectively, whereas green arrows represent the antiphase rotation of the FeO_6_ octahedra. (**d**) Top view showing FeO_6_ octahedral tilting, Bi ion positions, and the G-type antiferromagnetic ordering of Fe magnetic moments.

The crystal structure of BiFeO_3_ has been extensively investigated owing to its fundamental role in determining the material’s ferroic properties. The rhombohedrally distorted perovskite structure of BFO was first established by Michel et al. using single-crystal X-ray diffraction in 1969 [89] and was subsequently confirmed by Moreau et al. through neutron diffraction studies on polycrystalline samples [90]. At room temperature and atmospheric pressure, bulk BFO crystallizes in the polar rhombohedral *R3c* space group, which represents the thermodynamically stable phase under ambient conditions (Table 2). As illustrated in Figure 2, the crystal structure may be described using three alternative crystallographic descriptions: the rhombohedral (rh), hexagonal (hex), and pseudocubic (pc) unit cells, where the crystallographic directions [111]_rh_, [001]_hex_, and [111]_pc_, are equivalent, respectively. In the rhombohedral description, the unit cell contains two formula units and may be geometrically viewed as two distorted perovskite pseudocells stacked along the threefold polar axis. Among these settings, the pseudocubic notation is commonly adopted for describing polarization, domain structures, and magnetic ordering, whereas the rhombohedral and hexagonal settings are generally preferred for crystallographic analysis.

**Figure 3:**
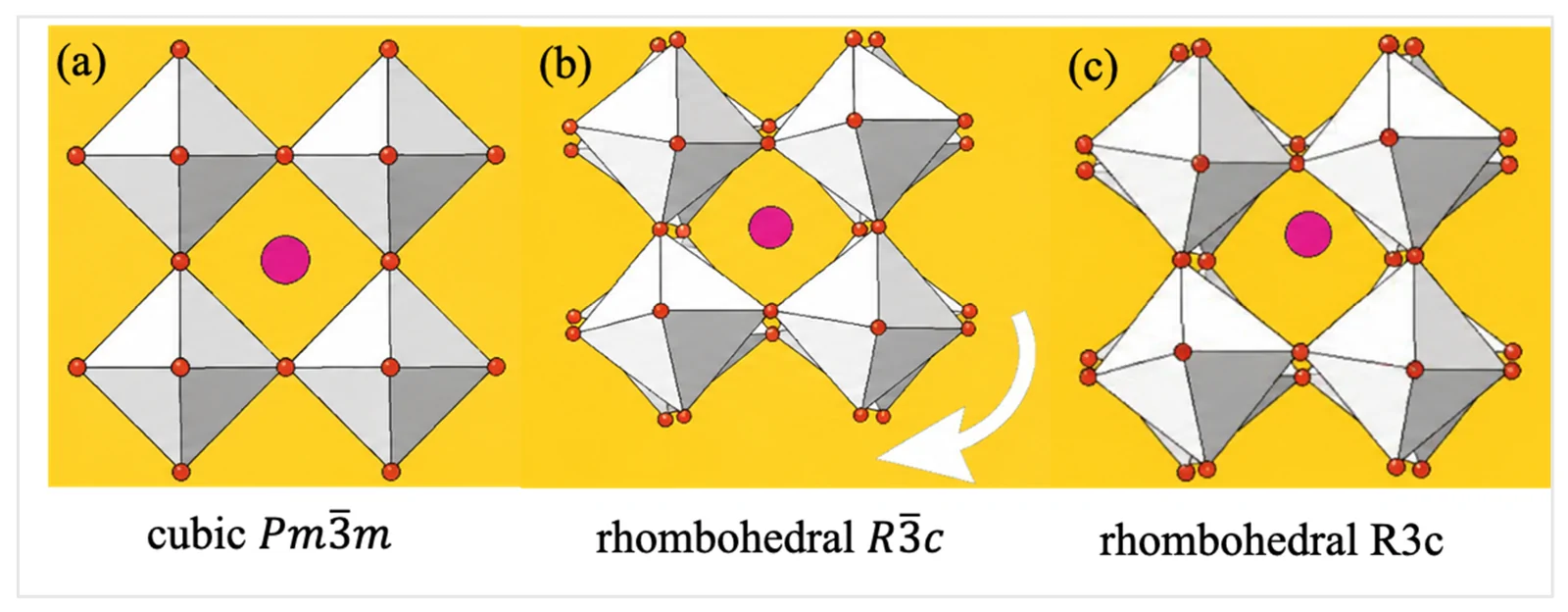
Schematic illustration of the structural distortions of the rhombohedral BiFeO_3_ ground state into its two primary lattice distortions: (**a**) ideal cubic perovskite $\mathrm{Pm}\bar{3}m$ structure without octahedral tilting (*a*^0^*a*^0^*a*^0^); (**b**) centrosymmetric rhombohedral ($R\bar{3}c$) structure generated by cooperative antiphase rotations of the FeO_6_ octahedra, corresponding to the Glazer tilt system (*a*^−^*a*^−^*a*^−^); and (**c**) polar rhombohedral (*R3c*) structure formed by the additional off-center displacement of the Bi^3+^ and Fe^3+^ cations along the pseudocubic [111] direction, resulting in spontaneous ferroelectric polarization. Adopted from [94].

The rhombohedral distortion of the BiFeO_3_ perovskite structure originates from the combined effects of ionic size mismatch, octahedral rotations, and the stereochemically active Bi^3+^ lone-pair electrons. The structural stability of perovskite oxides is commonly described by the Goldschmidt tolerance factor, $t=\;\frac{r_{A}+\;r_{O}}{\surd 2(r_{B}+\;r_{O})}$, where *r_A_*, *r_B_*, and *r_O_* represent the ionic radii of the A-site cation, B-site cation, and oxygen, respectively. For BiFeO_3_, the calculated tolerance factor $t=\;\frac{r_{\mathrm{Bi}}+\;r_{O}}{\surd 2(r_{\mathrm{Fe}}+\;r_{O})}$ is approximately 0.88 using Shannon’s ionic radii, typically considering Bi^3+^ in eightfold coordination and high-spin Fe^3+^ in sixfold coordination, indicating a significant deviation from the ideal cubic perovskite structure (t ≈ 1) [38,91]. The relatively small tolerance factor indicates that the ideal cubic perovskite framework is geometrically unstable, favoring cooperative antiphase rotations of the FeO_6_ octahedra about the three pseudocubic axes, corresponding to the Glazer tilt system *a*^−^,*a*^−^,*a*^−^ [92] with octahedral rotation angles typically ranging from approximately 11° to 14°, depending on the experimental conditions and structural refinement [93]. Considered independently, this nonpolar distortion lowers the symmetry of the ideal cubic *Pm*$\bar{3}m$ perovskite to the centrosymmetric rhombohedral *R*$\bar{3}c$ structure. The additional polar displacements of the Bi^3+^ and Fe^3+^ cations along the pseudocubic [111] direction remove inversion symmetry and give rise to the polar *R3c* ground state [38,89,93]. The stereochemically active 6*s*^2^ lone-pair electrons of the Bi^3+^ ions drive pronounced off-center displacements of the A-site cations, while the Fe^3+^ ions undergo much smaller off-center shifts within the oxygen octahedra. Structural refinements and first-principles calculations generally indicate Bi^3+^ off-center displacements of approximately 0.5–0.6 Å, whereas Fe^3+^ exhibits substantially smaller displacements of approximately 0.1–0.2 Å, although these values depend on the refinement method and measurement conditions. These cooperative ionic displacements generate a spontaneous polarization oriented along the pseudocubic [111] direction, constituting the microscopic origin of ferroelectricity in BiFeO_3_. Thus, the room-temperature *R3c* structure can be understood as arising from two coupled structural distortions: antiphase rotations of the FeO_6_ octahedra and polar displacements of the Bi^3+^ and Fe^3+^ cations, as schematically illustrated in Figure 3 [94].

These two structural distortions play distinct but complementary functional roles. Polar displacements of the Bi^3+^ and Fe^3+^ cations generate the spontaneous polarization, whereas FeO_6_ octahedral rotations modify the Fe–O–Fe bond angles and thereby control the strength and symmetry of the magnetic superexchange interactions between neighboring Fe^3+^ ions. Their coexistence within the same crystallographic framework establishes the structural conditions governing the ferroelectric polarization and Fe–O–Fe superexchange interactions in BiFeO_3_. Accordingly, even small variations in bond angles, displacement amplitudes, or local symmetry induced by temperature, pressure, defects, or chemical substitution can significantly modify its electrical, magnetic, and magnetoelectric response.

The crystallographic structure of BiFeO_3_ is also strongly dependent on temperature. With increasing temperature, gradual changes in the lattice parameters, unit-cell volume, Bi–O and Fe–O bond lengths, and Fe–O–Fe bond angles progressively modify the rhombohedral distortion and weaken the spontaneous polarization. At the Curie temperature (~1103 K), BiFeO_3_ undergoes a transition from the ferroelectric rhombohedral *R3c* phase to a paraelectric β phase, generally assigned to orthorhombic symmetry [95]. However, its exact space group remains debated because of the narrow stability range of the β phase and the onset of thermal decomposition at elevated temperatures. At still higher temperatures, a further transformation toward a higher-symmetry phase, frequently described as cubic or cubic-like, has been proposed. However, its precise symmetry and even its existence remain uncertain because BiFeO_3_ begins to decompose near this temperature, making experimental characterization particularly challenging [95]. These structural changes are accompanied by corresponding variations in the electrical, magnetic, and dielectric properties, highlighting the close relationship between crystal symmetry and the multifunctional behavior of BiFeO_3_. Recent work has further demonstrated that the *R3c* space-group symmetry is not merely a structural descriptor but a key parameter governing polarization, octahedral distortion, magnetic ordering, magnetoelectric coupling, and energy-related functionalities in BFO [96].

The close interplay between crystal structure and ferroic behavior makes structural engineering a powerful approach for tailoring the functional properties of BiFeO_3_. Variations in synthesis conditions, defect chemistry, grain size, and chemical substitution modify the octahedral rotation angles, polar displacement amplitudes, bond geometry, and local symmetry, thereby tailoring the ferroelectric, magnetic, dielectric, and magnetoelectric responses of the material. The polar distortions described above provide the microscopic basis of the dielectric and ferroelectric behavior of BiFeO_3_ examined in the following section.

**Table 2:** Selected structural and physical properties of BiFeO_3_.

Property	Information	Source
**Family**	Perovskite (ABO_3_)	[79]
**Crystal system**	Distorted rhombohedral perovskite with *R3c* space group (R phase) *a*rh = 5.6343 Å, αrh = 59.348° *a*pc = 3.965 Å, αpc = 89.35° ahex = 5.58 Å, chex = 13.90 Å, α = β = 90°, γ = 120°	[38,79,89,90,97]
**Space group**	*R3c*	[89,90]
**Tolerance factor (t)**	0.88	[79]
**FE Curie temperature (Tc)**	830 °C = 1103 K	[97]
**Polarization direction**	pseudocubic [111]	[38,89]
**Antiferromagnetic Néel temperature (T_N_)**	370 °C = 643 K	[79]
**Decomposition temperature**	934 °C	[98]
**Density (** ρ **)**	8.40 g cm^−3^	[96]
**Polyhedral volume ratio (VA/VB)**	4.727	[99]
**Optical band gap (Eg)**	2.1–2.8 eV	[100]
**Thermal expansion type**	Non-linear and anisotropic	[101]

### 3.2. Dielectric Properties of BFO Ceramics

Dielectric spectroscopy has played a pivotal role in elucidating the intrinsic dielectric response of bulk BiFeO_3_, while simultaneously revealing the significant influence of extrinsic phenomena arising from electrical conductivity and microstructural heterogeneity. In BiFeO_3_, the measured dielectric response often cannot be interpreted solely in terms of intrinsic lattice polarization because oxygen vacancies, mixed-valence Fe^2+^/Fe^3+^ ions, grain boundaries, and electrode interfaces promote defect-assisted charge transport and interfacial polarization that often dominate the measured response [86,102]. Consequently, establishing the intrinsic dielectric behavior of BFO has required progressively refined experimental approaches, including temperature-dependent impedance spectroscopy, broadband dielectric spectroscopy, infrared reflectivity, microwave spectroscopy, and contact-free dielectric techniques. By measuring the frequency and temperature dependence of the dielectric permittivity (ε′) and dielectric loss (tan δ), dielectric spectroscopy provides valuable information on polarization mechanisms, dielectric relaxation, electrical conductivity, and defect-related charge transport in BiFeO_3_.

**Figure 4:**
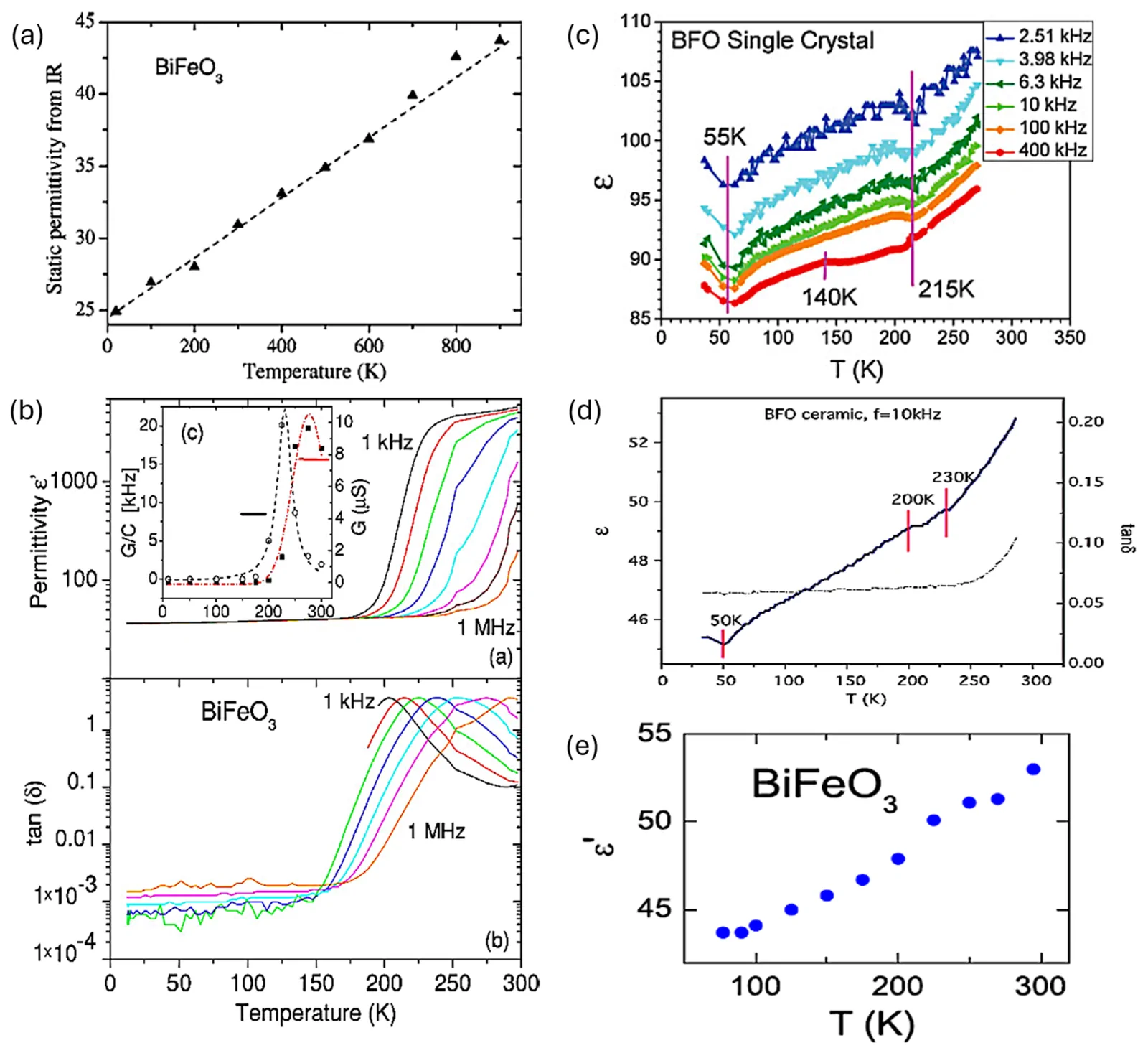
Landmark studies on the dielectric properties of bulk BiFeO_3_. (**a**) Temperature dependence of the intrinsic static dielectric permittivity determined from infrared reflectivity measurements, showing an approximately linear increase with temperature. (**b**) Temperature dependence of the dielectric permittivity (ε′) and dielectric loss (tan δ) measured at different frequencies, illustrating the strong frequency dispersion arising from extrinsic polarization and conductivity effects. (**c**) Low-temperature dielectric permittivity of BiFeO_3_ single crystals, revealing weak dielectric anomalies around 55, 140, and 215 K associated with spin-lattice interactions rather than additional ferroelectric phase transitions. (**d**) Corresponding dielectric response of polycrystalline BiFeO_3_ ceramics, showing analogous anomalies near 50, 200, and 230 K together with the dielectric loss, highlighting the influence of microstructure on the dielectric behavior. (**e**) Intrinsic dielectric permittivity of high-quality BiFeO_3_ single crystals obtained by contact-free millimeter-wave spectroscopy, providing a reliable estimate of the lattice dielectric constant, thereby minimizing Maxwell–Wagner and conductivity-related contributions. Panels (**a**,**b**) are reproduced from Kamba et al. [103], panels (**c**,**d**) from Redfern et al. [104], and panel (**e**) from Lu et al. [105].

One of the first comprehensive studies addressing this issue was carried out by Kamba et al. [103], who combined broadband dielectric spectroscopy with infrared reflectivity measurements to separate lattice contributions from extrinsic dielectric effects. Their work demonstrated that the intrinsic static dielectric permittivity (approximately 40–55 over the investigated temperature range) is considerably smaller than many values previously reported in the literature and exhibits an almost linear increase with temperature (Figure 4a,b). More importantly, they showed that the exceptionally large dielectric constants frequently measured at low frequencies cannot be explained by the lattice response alone, providing further evidence that interfacial polarization and electrical conductivity make substantial contributions to the dielectric spectra. These findings represented a major step toward distinguishing the intrinsic dielectric response of BiFeO_3_ from measurement artifacts associated with conductive samples and interfacial effects.

Further insight into the dielectric response of BiFeO_3_ was provided by Catalan and co-workers, who investigated the low-temperature dielectric behavior of both single crystals and ceramics, revealing weak dielectric anomalies occurring around 50 K, 140 K, and 200 K–230 K [104] (Figure 4c,d). These anomalies coincide with previously reported magnetic and elastic transitions and were interpreted as signatures of subtle spin-lattice interactions rather than evidence of additional ferroelectric phase transitions. The relatively small amplitude of these anomalies, together with the absence of significant changes in dielectric loss, indicates that the ferroelectric polarization remains essentially unaffected, whereas changes occur in the magnetic and elastic subsystems. This work considerably strengthened the emerging view that the dielectric response of BiFeO_3_ is intimately coupled to its magnetic order and lattice dynamics, providing an important experimental basis for understanding magnetoelectric coupling in this multiferroic system.

A further milestone was achieved by Lu et al. [105], who combined broadband dielectric spectroscopy with contact-free millimeter-wave measurements on high-quality single crystals in order to isolate the intrinsic dielectric response from Maxwell–Wagner contributions. Their results demonstrated that conventional low-frequency measurements above room temperature are largely dominated by interfacial polarization, whereas contact-free measurements yield an intrinsic dielectric permittivity of approximately 53 at room temperature, decreasing smoothly upon cooling (Figure 4e). Moreover, the electrical conductivity exhibited thermally activated behavior with an activation energy of about 0.39 eV together with a pronounced frequency dependence characteristic of Fe^2+^/Fe^3+^ hopping conduction, confirming that defect-assisted charge transport is the principal origin of the large dielectric losses commonly observed in BiFeO_3_. Importantly, no convincing evidence of intrinsic structural or ferroelectric phase transitions below room temperature was found, further supporting the interpretation that many previously reported dielectric anomalies originate from extrinsic effects or weak spin-lattice interactions rather than changes in the ferroelectric state.

The understanding developed from single-crystal investigations has subsequently been extended to polycrystalline ceramics, where dielectric spectroscopy has highlighted the critical role of the microstructure. In dense ceramics, semiconducting grains are typically separated by highly resistive grain boundaries, giving rise to Maxwell–Wagner interfacial polarization. Consequently, space-charge polarization dominates the low-frequency dielectric response, giving rise to very large dielectric permittivity. As the applied frequency increases, the accumulated interfacial charges can no longer follow the alternating electric field, resulting in a progressive decrease in both dielectric permittivity and dielectric loss. The dielectric losses are further enhanced by oxygen-vacancy migration and electron hopping between Fe^2+^ and Fe^3+^ ions, making the dielectric response strongly dependent on synthesis conditions, oxygen stoichiometry, grain size, and phase purity [106,107,108]. These observations underline that the dielectric behavior of bulk BiFeO_3_ is governed by a delicate interplay between intrinsic lattice polarization and extrinsic microstructural effects. Therefore, reliable determination of the intrinsic dielectric properties requires highly insulating samples together with high-frequency or contact-free dielectric techniques capable of minimizing interfacial polarization.

### 3.3. Ferroelectricity in BFO Ceramics

The ferroelectric state of BiFeO_3_ remains stable up to a Curie temperature of approximately 1103 K (Table 2), reflecting the remarkable stability of its polar state [89,90]. In addition, spontaneous polarization values approaching 60 μC cm^−2^ have been reported for high-quality single crystals and epitaxial thin films, placing BFO among the strongest ferroelectric perovskite oxides known to date [32,79]. Although bulk ceramics generally exhibit lower remanent polarization than single crystals or epitaxial thin films owing to leakage current, domain-wall pinning, porosity, and other microstructural defects, they provide an ideal platform for investigating how defect chemistry and microstructure influence the intrinsic ferroelectric response of BiFeO_3_.

As discussed in Section 3.1, the polar *R3c* structure permits a spontaneous polarization along the pseudocubic [111] direction [90,91], giving rise to eight energetically equivalent ferroelectric domain variants. These variants correspond to the body diagonals of the pseudocubic perovskite lattice. Within an individual ferroelectric domain, the spontaneous polarization is uniformly oriented, whereas adjacent domains are separated by narrow domain walls that minimize the total electrostatic and elastic energy of the crystal [8].

**Figure 5:**
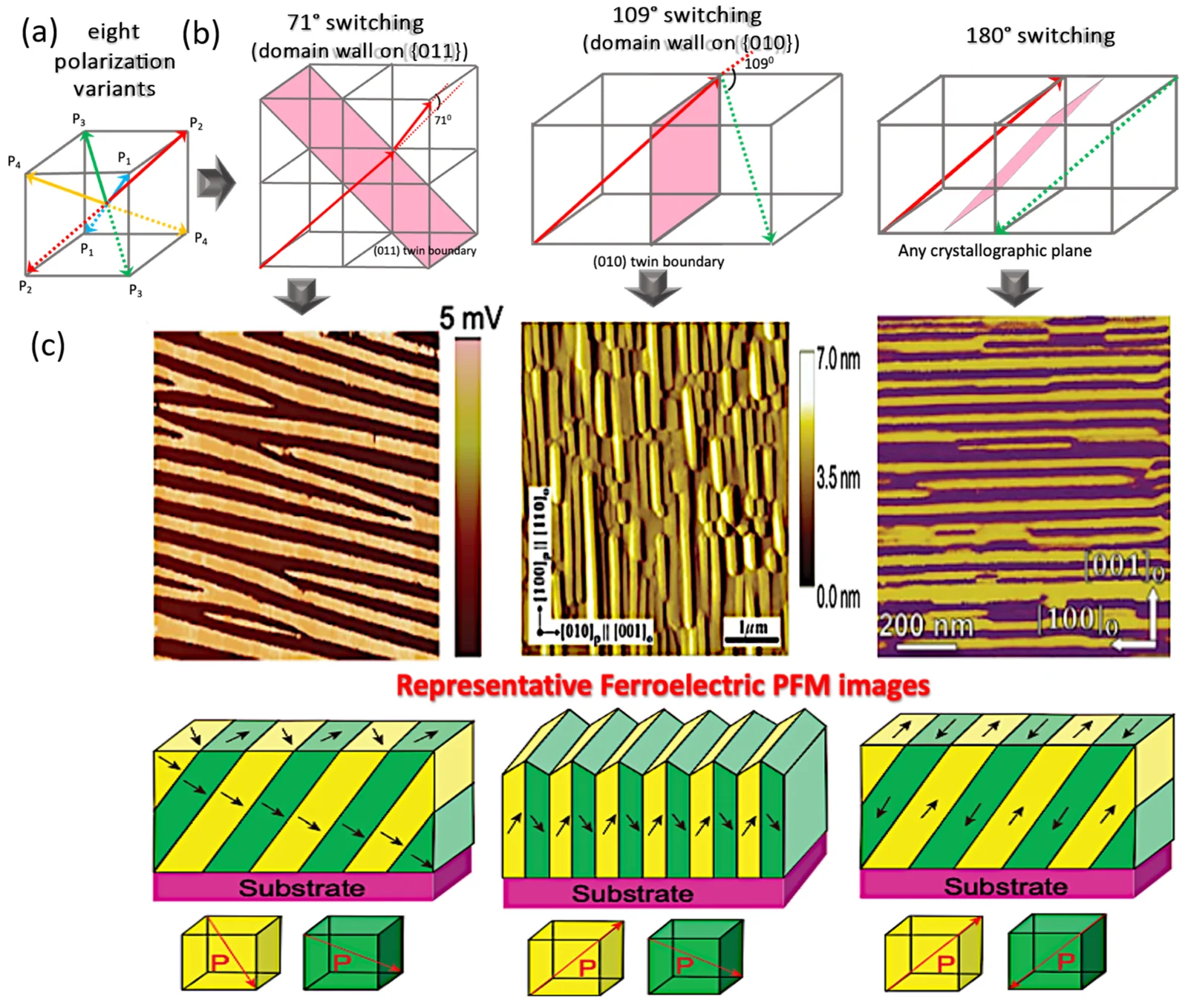
Ferroelectric domain variants and polarization switching mechanisms in rhombohedral BiFeO_3_. (**a**) Eight equivalent spontaneous polarization variants permitted by the rhombohedral *R3c* crystal symmetry, corresponding to the pseudocubic [111] directions. (**b**) Schematic illustration of the three crystallographically allowed polarization switching pathways (71°, 109°, and 180°), where the switching angle is defined by the angle between the spontaneous polarization vectors of adjacent domains. The preferred crystallographic orientations of the corresponding domain walls are also indicated. (**c**) Representative piezoresponse force microscopy (PFM) images together with the corresponding schematic domain configurations illustrating experimentally observed 71°, 109°, and 180° ferroelectric domain structures. (**a**,**b**) are adapted from Liu et al. [8], whereas (**c**) is adapted from Huyan et al. [109].

Polarization reversal between neighboring domains occurs through three crystallographically distinct switching pathways, namely 71°, 109°, and 180° switching. These switching angles are defined by the angle between the spontaneous polarization vectors of adjacent domains rather than by the orientation of the domain wall itself (Figure 5). A 71° switch involves reversal of one polarization component, a 109° switch involves reversal of two components, whereas a 180° switch corresponds to complete reversal of all three polarization components. Consequently, 71° and 109° switching are both ferroelectric and ferroelastic, because they involve changes in both polarization and spontaneous strain, whereas 180° switching is purely ferroelectric, since the spontaneous strain remains unchanged [8,109,110].

The crystallographic orientation of the domain walls is governed by simultaneous satisfaction of charge-neutrality and mechanical compatibility conditions. Accordingly, 71° domain walls preferentially lie on [110] planes, and 109° domain walls on [100] planes, whereas 180° domain walls may occur on crystallographic planes parallel to the spontaneous polarization direction. These crystallographic constraints govern the domain configurations commonly observed in BiFeO_3_ and strongly influence ferroelectric switching behavior and domain evolution under an applied electric field [8].

**Figure 6:**
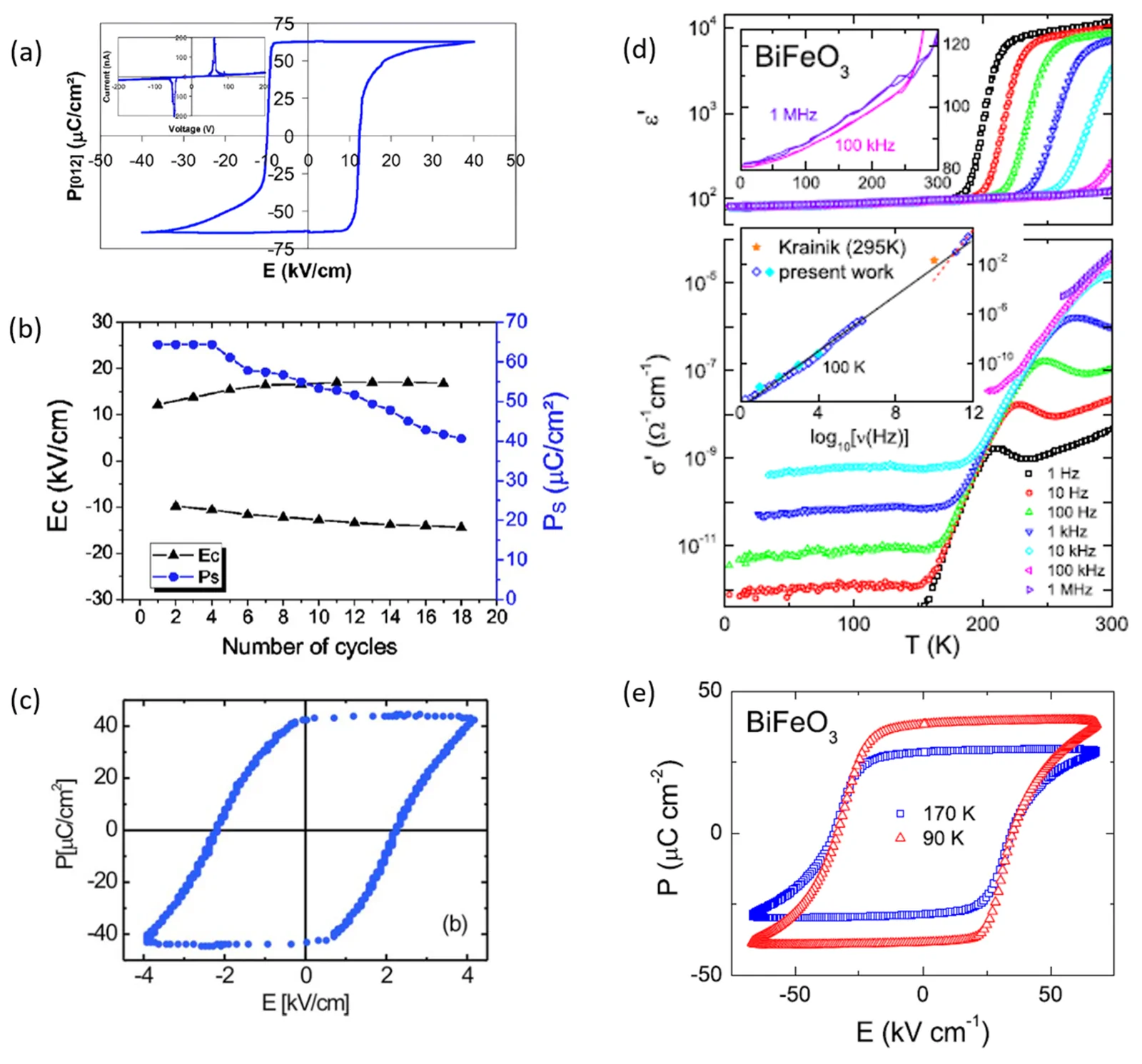
Landmark experimental studies on the ferroelectric behavior of bulk BiFeO_3_. (**a**) Room-temperature polarization–electric field (P–E) hysteresis loop measured on a high-quality BiFeO_3_ single crystal, demonstrating a remanent polarization of approximately 60 μC cm^−2^ and confirming the existence of a giant intrinsic polarization in bulk crystals. (**b**) Evolution of the coercive field and spontaneous polarization during repeated electric-field cycling. (**c**) Saturated P–E hysteresis loop obtained for optimized BiFeO_3_ ceramics, demonstrating that optimized bulk polycrystalline BiFeO_3_ can exhibit large switchable polarization despite the presence of grain boundaries. (**d**) Temperature- and frequency-dependent dielectric response together with the corresponding conductivity, highlighting the increasing influence of Maxwell–Wagner polarization and thermally activated charge transport at elevated temperatures. (**e**) Ferroelectric hysteresis loops measured below 200 K, where leakage-current contributions are significantly reduced, providing a reliable assessment of the intrinsic ferroelectric response of BiFeO_3_ single crystals. Panels (**a**,**b**) reproduced from Lebeugle et al. [78], panel (**c**) from Shvartsman et al. [114], and panels (**d**,**e**) from Lu et al. [105].

Early ferroelectric measurements performed during the late 1960s and early 1970s reported relatively small polarization values of approximately 6 μC cm^−2^ for single crystals, primarily because complete polarization saturation could not be achieved owing to leakage current, limited crystal quality, and experimental constraints [111]. More than three decades later, significant advances in crystal growth, thin-film deposition, and ceramic processing demonstrated that these low values did not represent the intrinsic behavior of BiFeO_3_. A major breakthrough was achieved by Lebeugle et al. [78,83], who reported the first well-saturated room-temperature ferroelectric hysteresis loops on high-quality BiFeO_3_ single crystals. Owing to the exceptionally high electrical resistivity of the crystals (6 × 10^10^ Ω cm), leakage-current effects were sufficiently suppressed to allow reliable polarization measurements. The authors measured a remanent polarization of approximately 60 μC cm^−2^ along the [012]_hex_ direction and inferred an intrinsic spontaneous polarization close to 100 μC cm^−2^ along the polar [001]_hex_ axis, in excellent agreement with first-principles calculations [112,113]. These results demonstrated that the giant polarization of BiFeO_3_ is an intrinsic property of the bulk crystal rather than a peculiar feature of epitaxial thin films (Figure 6a,b). Building upon these findings, Shvartsman et al. [114] demonstrated that an optimized low-temperature sintering route could produce high-quality BiFeO_3_ ceramics characterized by large, well-organized ferroelectric domains. Piezoresponse force microscopy (PFM) revealed a strong local electromechanical response, from which the authors estimated a polarization component of approximately 45 μC cm^−2^ along the measurement direction. After accounting for the crystallographic orientation of the polarization vector, an intrinsic spontaneous polarization of 75–80 μC cm^−2^ was inferred, in close agreement with first-principles calculations. This estimate was further supported by independent macroscopic polarization measurements using a conventional Sawyer–Tower circuit, which yielded saturation polarization values of approximately 40 μC cm^−2^ (Figure 6c). These findings provided evidence that the low polarization values previously reported for bulk BiFeO_3_ mainly resulted from extrinsic factors, including microstructural defects, porosity, random grain orientation, and incomplete polarization switching, rather than from intrinsic limitations of the material.

A further milestone was provided by Lu et al. [115], who carried out one of the most comprehensive investigations of the intrinsic dielectric and ferroelectric properties of high-quality BiFeO_3_ single crystals (Figure 6d,e). By minimizing the influence of leakage currents, the authors demonstrated that reliable ferroelectric hysteresis loops could be obtained below 200 K, yielding a remanent polarization close to 40 μC cm^−2^ in excellent agreement with previous single-crystal studies. Their dielectric investigation also clarified the origin of the extrinsic contributions affecting conventional dielectric measurements, providing a comprehensive framework for interpreting the intrinsic dielectric response of BiFeO_3_, as discussed in the previous section.

Together, these studies established that the intrinsic dielectric and ferroelectric behavior of bulk BiFeO_3_ had long been obscured by leakage currents, defect-assisted conduction, and Maxwell–Wagner polarization. When these extrinsic effects are minimized through optimized synthesis and appropriate experimental methodologies, bulk BiFeO_3_ exhibits a spontaneous polarization fully consistent with first-principles predictions and comparable to that of epitaxial thin films. These findings also highlight that the ferroelectric performance of bulk ceramics is controlled not only by the intrinsic crystal structure but largely by the nature and concentration of structural defects introduced during processing. In polycrystalline BiFeO_3_, oxygen vacancies, mixed-valence Fe^3+^/Fe^2+^ ions, grain boundaries, residual porosity, and secondary phases strongly influence domain-wall dynamics by acting as pinning centers that hinder polarization switching.

Fine-grained ceramics are particularly susceptible to these effects because the higher density of grain boundaries disrupts domain continuity and increases charge trapping. Consequently, obtaining well-saturated polarization–electric field (P–E) hysteresis loops in bulk BiFeO_3_ ceramics remains challenging, as leakage currents frequently mask the intrinsic ferroelectric response and hinder complete domain switching. Nevertheless, optimized synthesis and measurement conditions have enabled well-defined and, in some cases, saturated P–E loops to be obtained [78,105,114].

The recognition that defect chemistry and microstructure govern the macroscopic ferroelectric response has stimulated extensive efforts to optimize the processing of bulk BiFeO_3_ ceramics. Among the various strategies explored, chemical substitution has proven particularly effective for modifying defect chemistry, suppressing leakage current, and promoting more complete polarization switching [86,116,117,118]. The influence of chemical substitution on the structural, ferroelectric, magnetic, and multifunctional properties of BiFeO_3_ is discussed in detail in Section 4.

### 3.4. Antiferromagnetism in BFO Ceramics

BiFeO_3_ exhibits long-range antiferromagnetic ordering below its Néel temperature of approximately 643 K (Table 2) [119,120]. The magnetic moments originate from the high-spin Fe^3+^ (3d^5^) ions occupying the B-sites of the perovskite lattice. Below the Néel temperature, neighboring Fe^3+^ ions are coupled through superexchange interactions mediated by oxygen ions, resulting in a G-type antiferromagnetic structure, in which each Fe^3+^ spin is surrounded by six antiparallel nearest neighbors [119]. One of the most distinctive features of BiFeO_3_, however, lies in the complexity of its magnetic ground state, which extends well beyond that of a conventional G-type antiferromagnet. As discussed in Section 3.1, the magnetic behavior of BiFeO_3_ is intimately linked to its crystallographic structure. In particular, the strength of the Fe–O–Fe superexchange interaction depends sensitively on the Fe–O–Fe bond angle established by the cooperative rotation of the FeO_6_ octahedra. Consequently, variations in the crystal structure induced by temperature, strain, defects, or chemical substitution modify the superexchange strength and can significantly alter the magnetic response of BiFeO_3_. For many years, BiFeO_3_ was assumed to exhibit a simple collinear G-type antiferromagnetic structure. A major breakthrough came with the neutron diffraction study of Sosnowska et al. [119], who demonstrated that the magnetic ground state is instead characterized by an incommensurate cycloidal spin structure with a remarkably long modulation period of approximately 62–64 nm. In this magnetic configuration, the local G-type antiferromagnetic ordering is preserved, while the antiferromagnetic vector continuously rotates through the crystal, giving rise to a long-wavelength spin modulation. The cycloidal modulation propagates along three equivalent crystallographic directions corresponding to the propagation vectors (δ, −δ, 0), (δ, 0, −δ), and (0, −δ, δ), thereby generating three symmetry-equivalent magnetic domains [121,122]. Within each domain, the spins rotate inside a magnetic easy plane perpendicular to the corresponding propagation vector, forming the long-wavelength spin cycloid.

The microscopic origin of this unusual magnetic ground state was subsequently clarified by Ederer and Spaldin through first-principles calculations based on the local spin-density approximation (LSDA) and LSDA + U formalism [93]. Their calculations demonstrated that weak ferromagnetism is an intrinsic consequence of the non-centrosymmetric *R3c* crystal structure, which permits an antisymmetric Dzyaloshinskii–Moriya (DM) interaction inducing a slight canting (≈1°) of neighboring Fe^3+^ spins and consequently generates a weak local ferromagnetic moment [93,119,123]. However, this uncompensated magnetization does not develop into a measurable macroscopic magnetic moment because the long-wavelength cycloidal modulation periodically averages the local spin canting over one cycloidal period. As a result, bulk BiFeO_3_ exhibits an extremely small net magnetization despite the presence of local spin canting.

The magnetic ground state was further refined by high-resolution neutron diffraction studies. In particular, Ramazanoglu et al. [124] provided one of the most complete experimental descriptions of the cycloidal magnetic structure using single-crystal neutron diffraction. Their work accurately determined the propagation vector, the spin rotation plane, and the relationship between the magnetic modulation and the crystallographic symmetry, while providing a refined microscopic picture of the cycloidal ground state. These results consolidated the current understanding of bulk BiFeO_3_ as a G-type antiferromagnet locally modulated by a long-wavelength spin cycloid, establishing one of the most complete experimental descriptions of its magnetic structure currently available.

Since the cycloidal spin modulation effectively suppresses the observable magnetization, considerable efforts have been devoted to destabilizing or completely suppressing this magnetic structure through chemical substitution, epitaxial strain, particle-size reduction, and interface engineering [125,126,127]. Suppressing the cycloidal modulation releases the underlying weak ferromagnetic component, enhances the measurable magnetization, and often strengthens the magnetoelectric response. Consequently, controlling the stability of the cycloidal spin structure has become one of the most effective strategies for tailoring the multifunctional properties of BiFeO_3_, as suppressing the cycloid releases the latent weak ferromagnetic moment and significantly enhances the measurable magnetoelectric response, as discussed in the following section.

**Figure 7:**
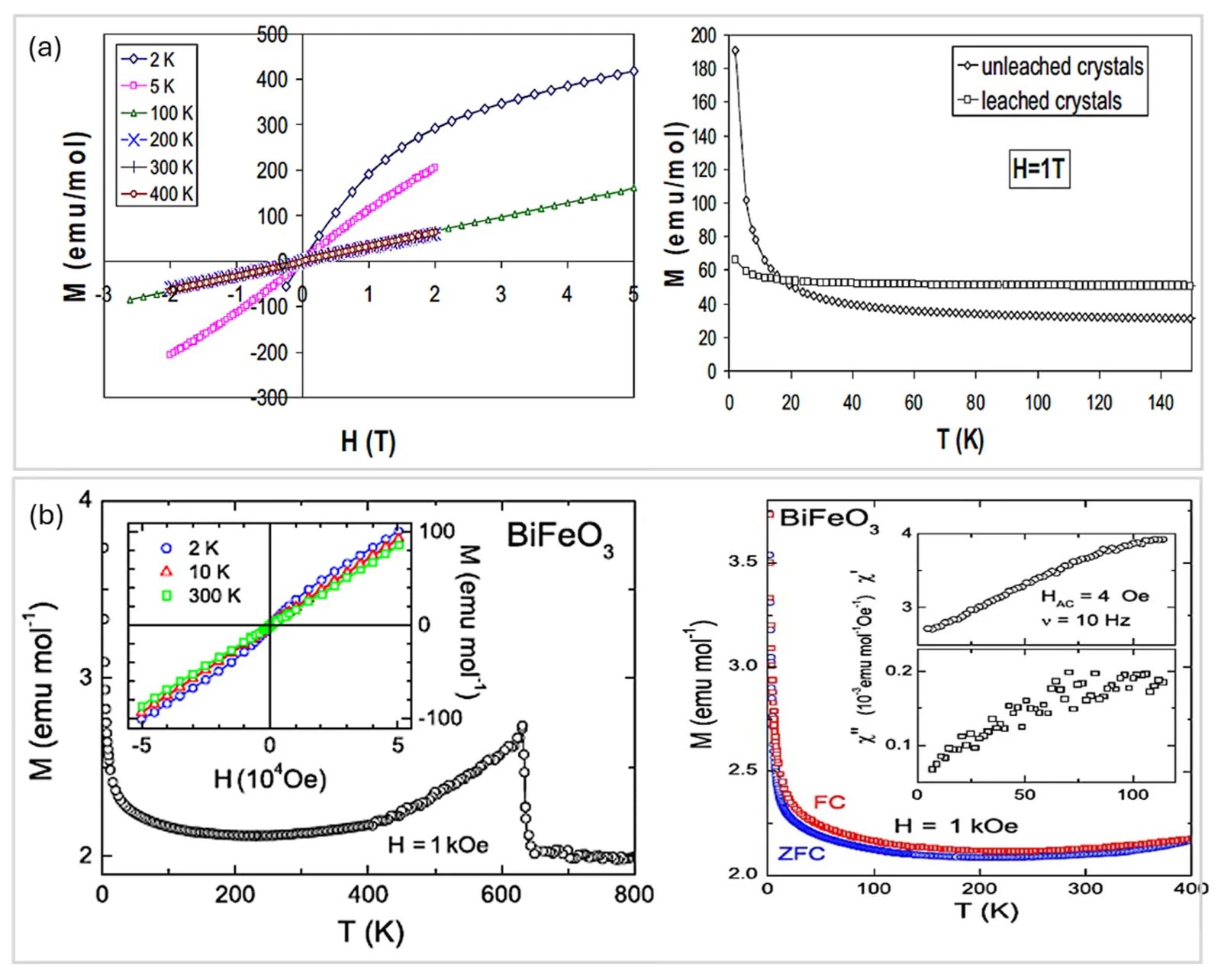
Landmark experimental studies on the magnetic properties of bulk BiFeO_3_. (**a**) Magnetic characterization of high-quality BiFeO_3_ single crystals reported by Lebeugle et al. [83]. **Left**: magnetization as a function of the applied magnetic field measured at different temperatures, showing an almost linear field dependence with negligible hysteresis, characteristic of the cycloidal antiferromagnetic ground state. **Right**: temperature dependence of the magnetization for untreated and chemically leached crystals, demonstrating that the removal of magnetic impurity phases markedly suppresses the low-temperature magnetic signal and confirms the intrinsically weak macroscopic magnetization of bulk BiFeO_3_. (**b**) Magnetic measurements performed by Lu et al. [115]. **Left**: temperature dependence of the dc magnetization measured up to 800 K, revealing the antiferromagnetic transition at the Néel temperature (≈643 K) together with the linear field dependence of the magnetization (inset). **Right**: overlapping zero-field-cooled (ZFC) and field-cooled (FC) magnetization curves together with the ac susceptibility (insets), providing strong evidence for the absence of intrinsic low-temperature spin-glass or spin-reorientation transitions in high-quality BiFeO_3_ single crystals.

The macroscopic magnetic response was subsequently clarified through high-quality magnetometry measurements on bulk single crystals, as illustrated in Figure 7. Lebeugle et al. [83] demonstrated (Figure 7a) that BiFeO_3_ exhibits an almost perfectly linear magnetization as a function of magnetic field up to several tesla, without measurable hysteresis, confirming the predominance of antiferromagnetic ordering despite the local spin canting predicted by theory. Complementary magnetic measurements by Lu et al. [115], shown in Figure 7b, included dc and ac susceptibility measurements on high-purity single crystals over an exceptionally broad temperature range (1.5–800 K). Their measurements revealed a clear antiferromagnetic transition at approximately 635 K, in excellent agreement with the accepted Néel temperature, followed by a smooth decrease in the magnetic susceptibility upon cooling without any additional anomalies. Moreover, field-cooled (FC) and zero-field-cooled (ZFC) magnetization curves were found to overlap over the entire investigated temperature range, while the ac susceptibility showed no frequency-dependent cusps or anomalies. These observations provided strong experimental evidence against the existence of intrinsic spin-glass freezing or spin-reorientation transitions in high-quality BiFeO_3_ single crystals, suggesting that several low-temperature magnetic anomalies previously interpreted as intrinsic magnetic transitions may instead originate from extrinsic effects, including oxygen non-stoichiometry, structural defects, or secondary magnetic phases.

Collectively, the studies of Lebeugle and Lu (Figure 7) established the intrinsic magnetic fingerprint of bulk BiFeO_3_: a robust G-type antiferromagnet with a long-wavelength cycloidal modulation, exhibiting an almost linear field dependence of the magnetization, a negligible macroscopic ferromagnetic moment, and no intrinsic low-temperature magnetic phase transitions. These benchmark results now provide experimental reference for assessing magnetic modifications induced by strain, chemical substitution, reduced dimensionality, or interface engineering.

### 3.5. Magnetoelectric Coupling in BFO Ceramics

Magnetoelectric (ME) coupling is one of the most distinctive characteristics of BiFeO_3_ because it enables coupling between the electric and magnetic order parameters within a single-phase material at room temperature. Owing to its high ferroelectric Curie temperature (T_C_ ≈ 1103 K) and antiferromagnetic Néel temperature (T_N_ ≈ 643 K), BiFeO_3_ has long been regarded as one of the most promising intrinsic multiferroics for electrically controlled spintronic, memory, and sensing applications [13,21,22,23,128,129]. Nevertheless, despite simultaneously exhibiting robust ferroelectricity and magnetic ordering, bulk BiFeO_3_ ceramics generally display only a weak measurable linear magnetoelectric response under ambient conditions. Consequently, improving the magnetoelectric coefficient (α_ME_) has become one of the central objectives in BiFeO_3_ research, with numerous studies focusing on chemical substitution and microstructural engineering to enhance the observable coupling effect while preserving the intrinsic ferroic properties.

As discussed in Section 3.4, the G-type antiferromagnetic ordering is modulated by a long-wavelength cycloidal spin structure with a periodicity of approximately 62–64 nm. Although the Dzyaloshinskii–Moriya (DM) interaction locally cants neighboring Fe^3+^ spins and generates weak ferromagnetic moments, the continuous rotation of spins within the cycloid causes these local moments to cancel over one cycloidal period. Consequently, the macroscopic linear magnetoelectric response is largely suppressed under low magnetic fields, and the induced polarization is dominated by a quadratic magnetic-field dependence (P ∝ H^2^) rather than the linear contribution associated with the linear magnetoelectric effect [129,130,131].

Direct experimental evidence for this mechanism has been obtained from high magnetic-field studies. When the applied magnetic field approaches approximately 18–20 T, the cycloidal spin modulation collapses and transforms into a homogeneous canted antiferromagnetic state [132]. Correspondingly, the magnetically induced polarization evolves from a predominantly quadratic dependence (P ∝ H^2^) to an approximately linear response (P ∝ H), revealing the strong influence of the cycloidal spin structure on the intrinsic magnetoelectric response [132]. Simultaneously, recovery of the weak ferromagnetic moment associated with DM spin canting provides further evidence that the magnetic cycloid strongly controls the observable macroscopic magnetoelectric response of bulk BiFeO_3_ [133,134].

These observations have fundamentally influenced the development of BiFeO_3_ ceramics over the past two decades. Instead of attempting to increase polarization or magnetization independently, efforts have focused on destabilizing the cycloidal spin structure through chemical substitution, phase-boundary engineering, strain, and microstructural optimization, thereby enhancing the observable magnetoelectric response [86,117,135,136,137,138].

Representative studies illustrating the evolution from conventional chemical substitution toward approaches combining compositional, structural, and defect engineering are summarized in Table 3. Early investigations showed that A-site substitution with divalent cations such as Sr or Ba produced a measurable transverse magnetoelectric coefficient (α_ME_), where the induced electric polarization is measured perpendicular to the applied magnetic field (α_ME_ ≈ 2.1 mV cm^−1^ Oe^−1^) [136]. The ME response was attributed by the authors to modifications of the magnetic/domain structure and magnetoelectric coupling.

Subsequent studies explored B-site substitution as a means of directly modifying the FeO_6_ octahedral network and altering the Fe–O–Fe superexchange interactions governing the magnetic ground state. Co-substituted BiFeO_3_ was reported to exhibit a pronounced magnetoelectric response, reaching α_ME_ = 11.3 mV cm^−1^ Oe^−1^ for BiFe_0.97_Co_0.03_O_3_. The authors attributed this response to Co-induced structural/magnetic modification and improved homogeneity resulting from the stirred hydrothermal synthesis route, which collectively contributed to the observable linear magnetoelectric response [137]. Similar improvements were reported for microwave-sintered La/Mn co-doped ceramics, where Bi_0.90_La_0.10_Fe_0.95_Mn_0.05_O_3_ exhibited α_ME_ = 10.11 mV cm^−1^ Oe^−1^, compared with 3.37 mV cm^−1^ Oe^−1^ for undoped BiFeO_3_ under identical measurement conditions. In this case, the enhancement was attributed to the combined effects of substantially reduced leakage current, improved insulation resistance, enhanced magnetic ordering, and microwave-induced lattice strain rather than to chemical substitution alone [138].

More recently, chemical substitutions have been increasingly employed to achieve phase-boundary engineering, in which the composition is tailored to stabilize the coexistence of two or more crystallographic phases (e.g., *R3c/Pna2_1_* or *R3c/Pna2_1_/Pbnm*) near a structural phase boundary. This approach aims not only to destabilize the cycloidal spin structure but also to exploit phase coexistence to favor ferroelectric switching, magnetic ordering, and the magnetoelectric response in bulk BiFeO_3_ ceramics.

A representative example is the (1−x)Bi_0.86_Sm_0.14_FeO_3_–xSrTiO_3_ system, in which the introduction of SrTiO_3_ into Sm-substituted BiFeO_3_ induced a structural evolution involving *Pna2_1_*, *R3c*, and *Pm*$\bar{3}m$ phases. In particular, at x = 0.05, the coexistence of these phases was accompanied by a remanent polarization of 32.28 μC cm^−2^, while the magnetoelectric coefficient (α_ME_) increased from approximately 0.20 mV cm^−1^ Oe^−1^ at the lowest applied DC magnetic field to a maximum value of 1.01 mV cm^−1^ Oe^−1^ at 2700 Oe. The authors attributed the observed magnetoelectric response to the synergistic improvement of the microstructure, ferroelectric, and magnetic properties associated with SrTiO_3_ modification [117]. Importantly, phase coexistence in BiFeO_3_ ceramics should not be inferred solely from peak splitting or peak broadening in XRD patterns, because such features may also arise from microstructural or compositional heterogeneity. In the SrTiO_3_-modified system, the evolution and coexistence of *Pna2_1_*, *R3c*, and *Pm*$\bar{3}m$ phases were evaluated using Rietveld refinement, providing stronger structural evidence than peak-shape changes alone [117]. In the multi-element La/Nd/Sm/Gd-substituted BiFeO_3_ system, the coexistence of *R3c* and *Pna2_1_* phases was likewise evaluated by Rietveld refinement, which provided quantitative phase fractions across the composition range [135]. For the x = 0.16 composition, this structural assignment was further supported by TEM/selected-area electron diffraction observations and by the uniform elemental distribution observed in the microstructure. Moreover, the change in the relative fractions of the *R3c* and *Pna2_1_* phases following electrical poling, together with the corresponding change in magnetization, provides additional evidence that the structural phases are functionally coupled to the electric-field-controlled magnetic response [135]. Nevertheless, these observations do not completely exclude local compositional or microstructural heterogeneity in polycrystalline ceramics. Therefore, enhancements in ferroic and magnetoelectric properties should be interpreted as arising from the combined effects of phase evolution, chemical substitution, defects, and microstructure rather than being attributed to phase coexistence alone.

Likewise, recent multi-element substitution strategies have demonstrated that careful control of the effective A-site ionic radius can stabilize the intermediate *Pna2_1_* phase, with important consequences for electric-field-controlled magnetism [86,135].

A representative example is Bi_1-x_(La_0.25_Nd_0.25_Sm_0.25_Gd_0.25_)_x_FeO_3_ ceramics. In this system, the effective ionic radius of the substituted rare-earth ions was designed to be approximately 1.100 Å, between the ionic radii of Nd^3+^ and Sm^3+^. With increasing substitution, the *R3c* phase fraction decreased while the *Pna2_1_* phase fraction increased. At x = 0.16, Rietveld refinement gave approximately 43.9% *R3c* and 50.5% *Pna2_1_*, indicating that the two phases have comparable fractions. The authors associated this composition with a small free-energy difference between the competing phases and proposed that such a condition could facilitate manipulation of the *Pna2_1_* → *R3c* transformation under an external electric field [135].

The role of the effective A-site ionic radius becomes clearer when the four-element La/Nd/Sm/Gd system, Bi_1−x_(La_0.25_Nd_0.25_Sm_0.25_Gd_0.25_)_x_FeO_3_, is compared with the five-element La/Nd/Sm/Gd/Lu system, Bi_1−x_(La_0.20_Nd_0.20_Sm_0.20_Gd_0.20_Lu_0.20_)_x_FeO_3_, reported in Ref. [86]. In the latter, the much smaller Lu^3+^ ion produces a larger difference between the substituted A-site ion and Bi^3+^, resulting in greater lattice distortion and stronger chemical pressure. The five-element system exhibits a direct *R3c* → *Pbnm* structural evolution without the intermediate *Pna2_1_* phase. In contrast, the four-element La/Nd/Sm/Gd system exhibits the *R3c* → *Pna2_1_* → *Pbnm* evolution. These results suggest that the effective A-site ionic radius and associated chemical pressure are key factors controlling whether the intermediate *Pna2_1_* phase can be stabilized [86,135].

This structural evolution is accompanied by changes in the ferroic properties. For the four-element system, a remanent polarization of 39.1 μC cm^−2^ was obtained at x = 0.14. At x = 0.16, the change in remanent magnetization after electrical poling reached 35.3 emu mol^−1^ (ΔMr = 53.3%). Moreover, XRD measurements before and after poling showed a change in the relative *R3c* and *Pna2_1_* contributions, while the magnetic measurements were consistent with an electric-field-induced *Pna2_1_* → *R3c* phase transition associated with the observed electric-field-controlled magnetism [135]. Importantly, the observed properties cannot be attributed to phase evolution alone. The multi-element substitution also reduced the leakage current by approximately five orders of magnitude compared with the undoped BiFeO_3_ reference [135], while the authors associated the improved ferroelectric properties with both the reduced leakage current and the coexistence of *R3c* and *Pna2_1_* phases. Thus, the reported improvement is better understood as arising from the combined effects of structural phase evolution and improved electrical properties rather than from phase coexistence alone.

Although these studies indicate that chemical modification can substantially improve the observable magnetoelectric response, direct comparison of the reported α_ME_ values should be made with caution. As evident from Table 3, the measurements were performed using different frequencies, AC magnetic-field amplitudes, DC bias fields, sample geometries, and electrical poling conditions, all of which significantly influence the measured magnetoelectric voltage coefficient. Furthermore, the measured response of bulk ceramics can contain both intrinsic contributions arising from coupling between electric and magnetic order parameters and extrinsic contributions associated with microstructural features such as grain boundaries, porosity, leakage, domain-wall motion, and mechanically or strain-mediated effects [139,140,141]. Accordingly, large reported α_ME_ values should not, by themselves, be regarded as direct evidence of stronger intrinsic magnetoelectric coupling. Consequently, differences in processing conditions and microstructure may also produce substantial variations in the measured magnetoelectric response even for nominally similar compositions. These observations highlight the importance of understanding how chemical substitution influences the underlying structure–property relationships governing the multifunctional behavior of bulk BiFeO_3_ ceramics. The following section examines these effects through A-site substitution, B-site substitution, and co-doping strategies.

**Table 3:** Representative strategies and reported magnetoelectric responses of bulk BiFeO_3_ ceramics. The table summarizes the composition, engineering strategy, measurement conditions, reported magnetoelectric coefficient (α_ME_), proposed origin of the ME response, and reported limitations for selected ceramic studies.

Composition	Engineering Strategy	ME Measurement Conditions	Peak α_ME_ (mV cm^−1^ Oe^−1^)	Proposed Origin of the ME Response	Reported Limitations
**Bi_0.7_Sr_0.15_Ba_0.15_FeO_3_** **[136]**	A-site Sr/Ba co-substitution	Dynamic lock-in technique; 7 kHz; AC field ≤ 10 Oe; DC bias up to ±3 kOe	2.1 (transverse) 0.28 (BFO)	modified magnetic domain structure and magnetoelectric coupling	Strong dependence on measurement geometry.
**BiFe_0.97_Co_0.03_O_3_** **[137]**	B-site Co substitution via stirred hydrothermal synthesis	Direct dynamic ME measurement	11.3 1.4 (BFO)	Co-induced structural/magnetic modification and cycloid suppression	Sensitive to Co content
**Bi_0.90_La_0.10_Fe_0.95_Mn_0.05_O_3_** **[138]**	La/Mn co-substitution with microwave sintering	Lock-in technique; 1 kHz; AC field = 1 Oe	10.11 3.37 (BFO)	Reduced leakage about 3 orders of magnitude; Cycloidal spin structure suppression; Enhanced magnetic response.	Response depends on La/Mn concentration and sintering conditions.
**(BiFeO_3_)–BaTiO_3_** **[142]**	Structural phase-boundary engineering	Dynamic lock-in technique; H_AC_ = 5 Oe; H_DC_ = 0.1–4.5 kOe; f = 1–10 kHz (αME-H_DC_ curves at 1 kHz); electrical poling (~3 kV mm^−1^) for BaTiO_3_ system	2.84 (x = 0.4, after electrical poling)	Cycloid suppression, Morphotropic/structural phase boundary, Electrical poling	Strong frequency dependence below 2 kHz
**Bi_1−x_Nd_x_FeO_3_** **[142]**	Nd-induced structural transition		2.70 (x = 0.2)	Cycloid suppression associated with rhombohedral–orthorhombic transition	ME response decreases away from optimum composition
**Bi_0.86_(LaNdSmGdLu)_0.14_FeO_3_** **[86]**	High-entropy-inspired multi-element A-site substitution	SuperME system; 2 kHz, H_AC_ = 1.07 Oe; RT; electrically poled	0.96	Reduced leakage and phase evolution	Strong dependence on substitution level and phase evolution
**0.95Bi_0.86_Sm_0.14_FeO_3_–0.05SrTiO_3_** **[117]**	SrTiO_3_-assisted phase-boundary engineering	SuperME system; 2 kHz	1.01 0.8 (BFO)	Triple-phase boundary; Improved microstructure.	Narrow composition window.
**Bi_0.84_(La_0.25_Nd_0.25_Sm_0.25_Gd_0.25_)_0.16_FeO_3_** **[135]**	Effective ionic-radius engineering through multi-element A-site substitution	Electric-field-controlled magnetization (no direct α_ME_ measurement)	α_ME_ not reported	Stabilization of the intermediate *Pna2_1_*	Strong dependence on A-.site effective ionic radius and phase evolution

*Note: Reported α_ME_ values were obtained under different experimental conditions and should not be directly compared as absolute performance metrics.*

## 4. Engineering the Functional Properties of BiFeO_3_ Through Chemical Substitution

The previous sections have shown that the multifunctional performance of bulk BiFeO_3_ is constrained by several interconnected intrinsic and processing-related factors, including Bi volatility during high-temperature sintering, the formation of oxygen vacancies and secondary phases, high leakage current, and the long-wavelength cycloidal spin structure that suppresses the macroscopic magnetoelectric response. Although optimization of synthesis and sintering conditions can improve phase purity, microstructure, and defect chemistry, it provides only limited control over the intrinsic structural and magnetic mechanisms governing the multifunctional response of BFO. Consequently, chemical substitution has emerged as a widely used and effective approach for tailoring the multifunctional behavior of bulk BiFeO_3_ by enabling simultaneous control of crystal structure, defect chemistry, electrical conductivity, magnetic ordering, and microstructure [17,18].

Importantly, chemical substitution in BiFeO_3_ directly alters the structural and electronic mechanisms governing its multifunctional response. Depending on the crystallographic site and nature of the dopant, substitution can modify the stereochemically active Bi^3+^ lone pair, alter Fe–O–Fe superexchange interactions, change lattice distortion and octahedral tilting, influence oxygen-vacancy formation, and destabilize the cycloidal spin structure [17,18]. Because these structural, electrical, and magnetic mechanisms are strongly coupled, modification at one crystallographic site frequently influences multiple ferroic properties simultaneously, leading to improved phase stability, reduced leakage current, enhanced ferroelectric polarization, enhanced magnetic response, and ultimately improved magnetoelectric performance [86].

**Figure 8:**
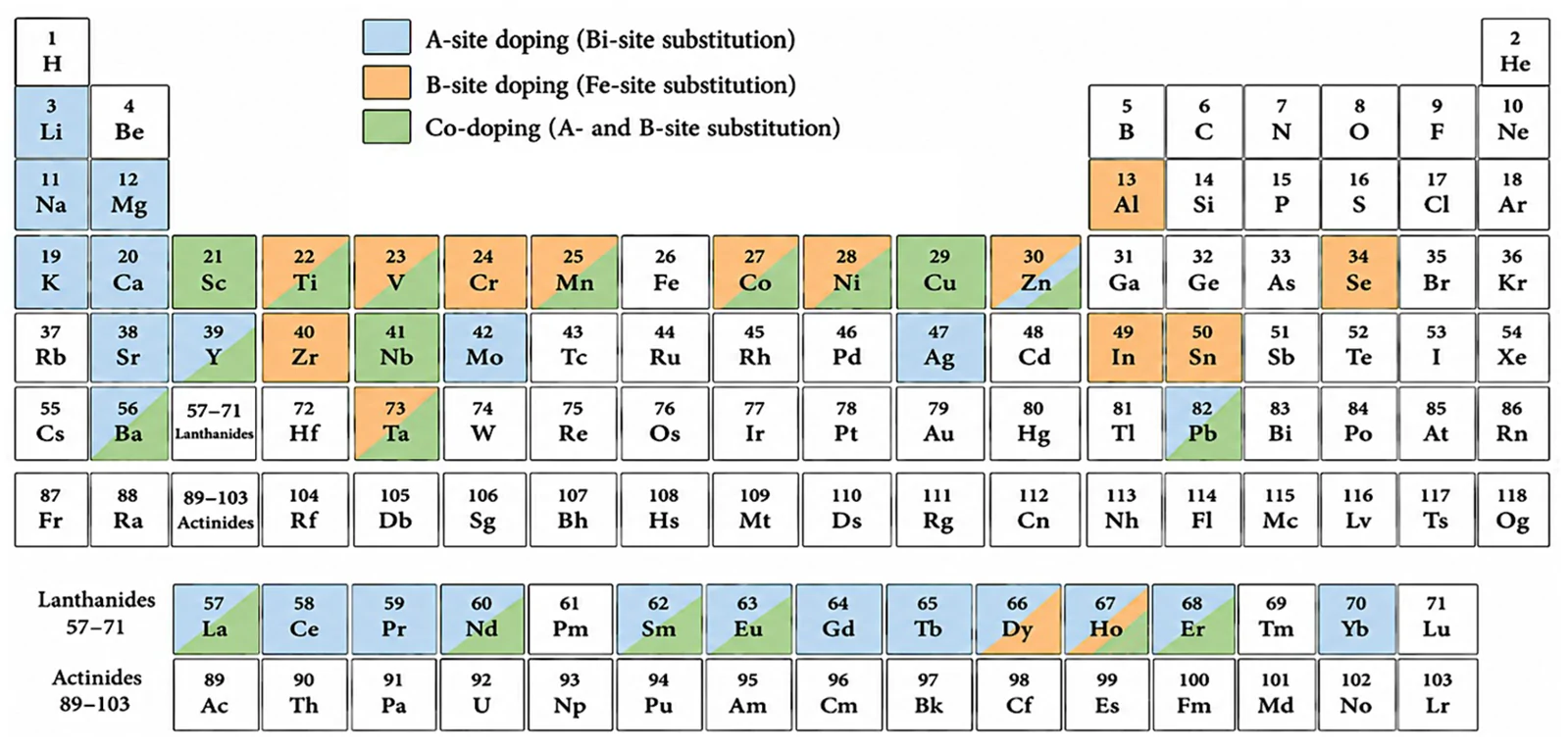
Periodic-table summary of the chemical elements investigated for substitution in bulk BiFeO_3_ ceramics. Blue, orange, and green indicate elements reported for A-site substitution, B-site substitution, and simultaneous A- and B-site co-doping, respectively, based on the literature reviewed in this work (Table 4, Table 5 and Table 6). Elements appearing in more than one category reflect their use in multiple substitution strategies.

From a crystal-chemical perspective, the effectiveness of chemical substitution is governed primarily by three factors: ***(i)*** the ionic radius of the dopant, which determines the degree of lattice distortion and internal chemical pressure; ***(ii)*** the dopant valence, which controls charge compensation and defect formation, particularly oxygen vacancies; and ***(iii)*** the crystallographic site occupied by the dopant, which dictates whether the modification predominantly affects the ferroelectric distortion associated with the Bi sublattice or the magnetic exchange interactions within the FeO_6_ octahedral framework [17,18]. Accordingly, substitution strategies are generally classified into A-site substitution, B-site substitution, and simultaneous A- and B-site substitution (co-doping). Figure 8 summarizes the chemical elements investigated for each substitution strategy in the literature reviewed in this work, while the corresponding structural evolution and multifunctional properties are discussed in Section 4.1, Section 4.2 and Section 4.3 and summarized in Table 4, Table 5 and Table 6.

### 4.1. A-Site (Bi-Site) Substitution

#### 4.1.1. Structural Effects of A-Site Substitution

A-site substitution directly targets the Bi sublattice and has been widely explored to improve phase stability and control defect formation in bulk BiFeO_3_. In this approach, Bi^3+^ ions are partially replaced by suitable cations, typically at low substitution levels (generally ≤ 10 mol%), to stabilize the perovskite lattice while suppressing defect formation. Besides improving phase stability, A-site substitution introduces lattice distortion through ionic-size mismatch and local strain, thereby influencing the dielectric, ferroelectric, magnetic, and magnetoelectric properties of BiFeO_3_ [18].

A frequently reported effect of A-site substitution is the suppression of secondary phases and improvement of phase purity. This trend has been reported for a wide range of rare-earth dopants, including La^3+^ [14,143,144], Ce^4+^ [145,146], Sm^3+^ [147,148], Gd^3+^ [149], Nd^3+^ [150], Yb^3+^ [151,152,153], Eu^3+^ [154], Er^3+^ [144], and Ho^3+^ [155], as well as aliovalent substitutions such as Ba^2+^ [156], Ca^2+^ [157], and Pb^2+^ [158]. Despite differences in ionic radius and valence state, suppression of secondary phases and improved phase purity emerge as common effects across a wide range of A-site dopants. In addition to improving phase stability, A-site substitution also modifies the crystal symmetry through dopant-induced lattice distortion, although the structural evolution depends strongly on the ionic radius, valence state, and concentration of the substituting cation. As summarized in Table 4, the parent rhombohedral *R3c* structure is often retained at low substitution levels for many dopant systems, whereas increasing the dopant concentration can promote greater lattice distortion, mixed-phase regions, or structural transitions toward orthorhombic, tetragonal, or pseudo-tetragonal symmetries [144,147,150,154]. This dopant-dependent structural evolution can be illustrated by several representative examples. Ba substitution preserves the rhombohedral perovskite structure while eliminating Bi_2_O_3_ and Bi_2_Fe_4_O_9_ secondary phases and gradually shifting the diffraction peaks toward lower diffraction angles, indicating lattice expansion and structural distortion without a complete crystallographic phase transition [156]. In contrast, Nd substitution produces a progressive symmetry evolution from the rhombohedral *R3c* phase through an intermediate polar *Pna2_1_* phase toward the orthorhombic *Pbnm* structure via morphotropic phase boundaries [150]. Eu substitution follows a different structural pathway, where increasing Eu concentration induces a rhombohedral-to-orthorhombic (*Pn2_1_a*) transition accompanied by lattice contraction near the optimum substitution level [154]. Collectively, these representative systems demonstrate that although suppression of secondary phases is a common consequence of A-site substitution, the accompanying structural evolution is highly dopant-dependent, ranging from subtle lattice distortion to symmetry modulation and crystallographic phase transformation.

#### 4.1.2. Functional Properties of A-Site Substituted BiFeO_3_

The structural changes discussed above are directly reflected in the dielectric, ferroelectric, magnetic, and magnetoelectric properties of BiFeO_3_, although the extent of these improvements depends strongly on the nature and concentration of the A-site dopant.

As shown in Table 4, numerous A-site substitutions have been reported to modify—and in optimized compositions enhance—dielectric and/or ferroelectric properties relative to undoped BFO [14,143,146,149,151,158]. Ba-, Nd-, and Eu-substituted systems provide representative examples of how these functional enhancements can arise through different structural mechanisms rather than from substitution alone. In Ba-substituted BiFeO_3_, the dielectric constant reaches its maximum at *x* = 0.20, coinciding with the appearance of a well-saturated ferroelectric hysteresis loop with a remanent polarization of approximately 15 μC cm^−2^. Further Ba addition (*x* = 0.25) produces a larger apparent polarization, but this is accompanied by increased leakage current rather than improved intrinsic ferroelectric switching [156]. In Nd-substituted BiFeO_3_, the optimum ferroelectric response occurs near the morphotropic phase boundaries, where the remanent polarization reaches approximately 33.8 μC cm^−2^ at x = 0.10 and remains high (32.2 μC cm^−2^) at x = 0.12 owing to electric-field-induced transformation between the intermediate *Pna2_1_* and rhombohedral *R3c* phases. The PFM images further corroborate this behavior by revealing a clear evolution of the ferroelectric domain morphology across the morphotropic phase boundary, consistent with the enhanced switchable polarization observed near the optimum Nd concentration [150]. In contrast, Eu substitution exhibits its maximum dielectric response at *x* = 0.20, corresponding to the onset of the rhombohedral-to-orthorhombic structural transition, after which the dielectric constant decreases with further Eu addition [154]. These representative systems demonstrate that the functional response is intrinsically dopant-dependent, with structural distortion, symmetry modulation, or phase transformation governing the dielectric and ferroelectric behavior depending on the substitution chemistry. Consequently, the optimum performance is achieved only within a limited composition range rather than increasing monotonically with dopant concentration.

The magnetic response exhibits even greater composition dependence because it is strongly influenced by structural distortion and the resulting modification of the Fe–O–Fe magnetic exchange pathways. As summarized in Table 4, enhanced magnetic properties have been reported for numerous rare-earth and divalent-cation substitutions [144,147,149,151,152,155,159], although the magnitude of improvement varies considerably with dopant species, concentration, and processing conditions. The Ba-, Nd-, and Eu-substituted systems discussed above also provide representative examples of this composition-dependent magnetic behavior, with magnetic enhancement following distinct structure-dependent pathways. In Ba-substituted BiFeO_3_, the remanent and saturation magnetization initially increase, reaching maximum values of approximately 0.65 emu g^−1^ and 1.25 emu g^−1^, respectively, at *x* = 0.15 before decreasing at higher Ba concentrations. This behavior has been attributed to changes in Fe^2+^/Fe^3+^ interactions, spin canting, and partial suppression of the cycloidal spin structure [156]. Similarly, Nd substitution produces optimum magnetic performance near the morphotropic phase boundaries (*x* ≈ 0.22), where the remanent magnetization reaches approximately 91.7 emu g^−1^, which was attributed by the authors to suppression of the spiral spin structure, followed by a decline as the orthorhombic *Pbnm* phase becomes dominant [150]. Eu substitution likewise exhibits maximum saturation magnetization (1.8 emu g^−1^) and remanent magnetization (0.6 emu g^−1^) at *x* = 0.20, corresponding to the structural transition region where distortion of the Fe–O–Fe bond geometry and collapse of the spin cycloid promote weak ferromagnetism before the magnetic properties deteriorate at higher Eu concentrations [154]. These observations demonstrate that magnetic enhancement is primarily governed by dopant-induced structural distortion and the associated modification of magnetic exchange interactions rather than by the intrinsic magnetic moment of the substituting ion.

The studies discussed above, together with the broader comparison summarized in Table 4, demonstrate that the functional properties of BiFeO_3_ can be effectively tailored through A-site substitution; however, the magnitude and origin of the improvements are strongly dependent on the dopant chemistry and the resulting structural evolution. Rather than following a universal trend, different dopants enhance the multifunctional response through distinct mechanisms, including lattice distortion, symmetry modulation, and crystallographic phase transitions. A further extension of A-site substitution involves increasing composition complexity through the simultaneous incorporation of multiple cations. Recently, high-entropy-inspired A-site substitution was demonstrated in BiFeO_3_ through the incorporation of La, In, Y, Nd, and Gd, showing that compositional complexity can modify the structural, dielectric, and magnetic properties of bulk BFO [160]. Because A-site substitution primarily alters the Bi sublattice and its associated structural environment, increasing compositional complexity provides an additional route for tuning the structure, dielectric, and magnetic responses of BFO. However, its influence on the Fe–O–Fe magnetic exchange network remains indirect, providing a clear rationale for the B-site substitution strategies discussed in the following section.

### 4.2. B-Site (Fe-Site) Substitution

#### 4.2.1. Structural Effects of B-Site Substitution

Unlike A-site substitution, which primarily modifies the Bi sublattice to improve structural stability and suppress defect-mediated electrical conduction, B-site substitution directly alters the FeO_6_ octahedral framework that governs the magnetic exchange interactions in BiFeO_3_. Since the antiferromagnetic ordering originates from Fe^3+^–O–Fe^3+^ superexchange, partial replacement of Fe^3+^ provides a direct crystal-chemical route for modifying the local octahedral environment and consequently the magnetic exchange pathways. Therefore, Fe-site substitution has emerged as a complementary strategy for tailoring the multifunctional properties of BiFeO_3_ through direct engineering of the magnetic sublattice [17,18].

The structural results summarized in Table 5 indicate that for most investigated dopants, the rhombohedral *R3c* perovskite structure remains stable over the reported substitution ranges, including Mn^2+^ (up to 20 mol%) [161], Ti^4+^ (25 mol%) [162], V^5+^ (3 mol%) [163], Ta^5+^ (3 mol%) [164], Zn^2+^ (10 mol%) [165], Ho^3+^ (15 mol%) [118], and Zr^4+^ (10 mol%) [166], although measurable variations in lattice parameters and octahedral distortion are commonly observed. By contrast, Dy substitution induces a rhombohedral-to-orthorhombic structural transformation at only 5 mol% substitution [167], whereas Co substitution exhibits a progressive structural evolution from rhombohedral (0 ≤ x ≤ 0.2) to monoclinic (x = 0.3) and ultimately to a tetragonal BiCoO_3_-type phase (0.5 ≤ x ≤ 0.6) with increasing Co concentration [168]. These observations demonstrate that structural evolution is dictated primarily by the crystal chemistry of the individual dopant rather than by B-site substitution itself.

The influence of B-site substitution on phase purity likewise varies with the substituting ion (Table 5). Several compositions, particularly Mn-, Ho-, and Zr-substituted BiFeO_3_, exhibit improved phase formation with suppression of secondary phases, whereas Ti-, Ta-, Zn-, and Ni-containing ceramics still contain detectable secondary phases, especially at higher substitution levels [118,162,164,165,166,168,169]. In the Co system, the dominant structural change is crystallographic phase evolution rather than simple impurity formation [168]. Collectively, these observations demonstrate that both phase evolution and phase purity are highly dopant-dependent, reflecting the combined influence of the substituting ion and the processing conditions. The implications of these structural changes for the dielectric, ferroelectric, and magnetic properties are discussed in the following section.

#### 4.2.2. Functional Properties of B-Site-Substituted BiFeO_3_

Fe-site substitution alters the dielectric, ferroelectric, and magnetic responses through dopant-dependent modifications of the crystal structure, defect chemistry, and Fe–O–Fe exchange interactions. Two representative examples illustrate how B-site substitution modifies the structure–property relationships of BiFeO_3_ through distinct mechanisms. Mn-substituted BiFeO_3_ was reported by Deng et al. [170], whereas Ho-substituted BiFeO_3_ was investigated by Pradhan et al. [118]. Although both dopants occupy the Fe site, they produce different structural evolutions and consequently different multifunctional responses.

For the Mn-substituted system, XRD analysis reveals a gradual structural evolution from the rhombohedral *R3c* phase toward the rhombohedral *R3m* phase with increasing Mn concentration, accompanied by improved crystallinity and preferred grain orientation. This structural modification has been attributed to Mn incorporation into the Fe sublattice, which also reduces the concentration of oxygen vacancies. The dielectric spectra exhibit systematic changes with increasing Mn content, while the M–H loops show enhanced magnetic hysteresis compared with undoped BiFeO_3_. Nevertheless, the hysteresis loops remain unsaturated, indicating that the Mn-substituted ceramics retain their predominantly antiferromagnetic character with only weak ferromagnetic contributions arising from the local structural distortion and reduced defect concentration. In contrast, increasing Ho content progressively suppresses the secondary phases while preserving the rhombohedral *R3c* crystal structure. Correspondingly, increasing Ho concentration leads to a higher dielectric constant, enhanced magnetization, and progressively better-defined ferroelectric P–E hysteresis loops, indicating reduced leakage current and improved ferroelectric switching behavior. These improvements were attributed to suppression of Fe^2+^ formation, reduced oxygen-vacancy concentration, stabilization of the FeO_6_ octahedral network, and the resulting decrease in leakage current, with x = 0.15 exhibiting the optimum multifunctional performance.

Though Mn^2+^ and Ho^3+^ illustrate two distinct structure–property relationships, the wider literature demonstrates that the dielectric response varies substantially with the nature of the B-site dopant, with some substitutions increasing dielectric permittivity, others primarily reducing dielectric loss, and a few improving both simultaneously. For example, Mn substitution at *x* = 0.20 increased the dielectric permittivity from about 10 to 50 at 1 MHz, although the dielectric loss remains stable at 1 [161]. Ho substitution at x = 0.15 increased the dielectric constant to approximately 240 at 1 kHz [118], while it remains almost stable at 1 MHz (about 30 with losses in the range 0.1–0.15). Ti^4+^ substitution (*x* = 0.25) produced dielectric constants of approximately 250 at 1 MHz with relatively low dielectric losses (0.03) [162]. In general, the dielectric response depends strongly on the substituting ion and its substitution level.

As far as ferroelectric properties are concerned, Ho substitution provides one of the clearest improvements, with BiFe_0.85_Ho_0.15_O_3_ exhibiting a remanent polarization of approximately 8.4 μC cm^−2^ together with a well-developed hysteresis loop [118].

The trends observed for the representative Mn- and Ho-substituted systems are also reflected in the broader literature summarized in Table 5. Compared with the dielectric and ferroelectric responses, enhancement of the magnetic properties is more consistently reported across a wide range of B-site dopants. For example, Dy substitution (x = 0.05) increased the remanent magnetization to 1.45 emu g^−1^ and a magnetization value of 49 emu g^−1^ was recorded at 20 kOe [167]. Mn substitution induces a weak ferromagnetic contribution to the predominantly antiferromagnetic response of BiFeO_3_, with the remanent magnetization reaching a maximum of 5.5 × 10^−3^ emu g^−1^ at x = 0.10 before decreasing at higher substitution levels [161]. Ho substitution (x = 0.15) produced Ms ≈ 0.364 emu g^−1^ with Mr ≈ 0.0258 emu g^−1^ [118], whereas Ni substitution increased the remanent magnetization from 0.006 to 0.10 emu g^−1^ together with a saturation magnetization of approximately 0.41 emu g^−1^ at x = 0.05 [169].

As discussed in Section 3.3 and Section 3.5, these improvements are primarily associated with modification of the Fe–O–Fe exchange pathway, enhanced Dzyaloshinskii–Moriya spin canting, and partial suppression of the cycloidal spin structure, depending on the specific dopant and composition. Nevertheless, the extent of magnetic enhancement remains highly system-dependent, reflecting the combined influence of dopant chemistry, substitution level, and the associated lattice distortion [17].

### 4.3. A- and B-Site Co-Substitution

#### 4.3.1. Structural Effects of A- and B-Site Co-Substitution

Although single-site substitution effectively improves selected properties of BiFeO_3_, neither A-site nor B-site engineering alone can simultaneously optimize its multifunctional response. A-site substitution primarily enhances structural stability and suppresses defect-mediated electrical leakage, whereas B-site substitution directly modifies the Fe–O–Fe exchange interactions responsible for magnetic ordering [17,18]. Their complementary effects have therefore motivated the development of co-doping, in which suitable cations are simultaneously introduced at both crystallographic sites to engineer structural stability, defect chemistry, electrical transport, and magnetic interactions in a cooperative manner.

As summarized in Table 6, a wide range of co-doping combinations have been explored, most commonly pairing rare-earth ions at the A site with transition-metal ions at the B site [163,171,172,173,174,175,176,177,178,179,180,181,182,183,184,185]. Compared with undoped BiFeO_3_, many co-doped systems exhibit improved phase purity and enhanced properties in one or more functional responses resulting from the cooperative engineering of both crystallographic sublattices. Structurally, simultaneous substitution provides greater flexibility for tuning the perovskite lattice than single-site doping, enabling rhombohedral-to-monoclinic, orthorhombic, tetragonal, or mixed-phase structures depending on the dopant combination and concentration [163,171,172,174,175]. For example, Ba^2+^/Ta^5+^ and Eu^3+^/Ti^4+^ substitutions promote rhombohedral-to-tetragonal evolution at higher dopant concentrations [171,172,186]. Likewise, Sm^3+^/Sc^3+^ and Nd^3+^/Nb^5+^ co-doping produce orthorhombic (*Pnma*) and mixed *R3c–Pnma* structures, respectively, illustrating that A- and B-site substitution offers an effective route for tailoring lattice distortion and phase stability [175,187].

#### 4.3.2. Functional Properties of Co-Substituted BiFeO_3_

While the structural effects of co-substitution provide the basis for phase stabilization and lattice engineering, their practical significance ultimately lies in the resulting enhancement of the functional properties. The cooperative interaction between A- and B-site substitutions enables the simultaneous optimization of multiple ferroic responses, although the magnitude of these improvements remains strongly composition-dependent. The influence of co-substitution is particularly evident in the dielectric response, which exhibits a pronounced dependence on both the dopant combination and its concentration. Several co-doped systems show a simultaneous enhancement of dielectric permittivity and reduction in dielectric loss, although the optimum performance generally occurs within a limited composition range. For example, Zn/Ti co-doping increases the dielectric constant at 1 MHz from approximately 40 to 85 at *x* = 0.025 [188], while Ba/Ta co-doping increases it from 110 to 206 at *x* = 0.1 before decreasing with further substitution [172]. Likewise, Nd/Mn co-doping raises the dielectric constant from 45 to 160 at 1 MHz while reducing dielectric loss from 0.112 to 0.008 [180], as do Eu/Ti substitutions in which the dielectric constant increases from 20 to 320 at x = 0.15, followed by a small reduction in the dielectric loss from 0.1 to 0.07 [186]. However, not all co-doping systems follow this trend; La/Er exhibit a gradual reduction in dielectric constant with increasing dopant concentration [171]. These composition-dependent dielectric behaviors are further illustrated by the representative systems, where Ba/Ta primarily enhances dielectric permittivity at intermediate compositions, Eu/Ti effectively suppresses dielectric loss through defect engineering, and La/Er demonstrates that structural modification alone does not guarantee an increase in dielectric constant, highlighting the important role of defect chemistry, in addition to structural modification and dopant concentration, in determining the dielectric response. The ferroelectric response of co-doped BiFeO_3_ ceramics varies markedly with dopant combination and concentration, and the available data do not indicate a systematic enhancement upon co-substitution (Table 6). La/Er doping provides a clear example of a progressive weakening of ferroelectricity: although well-defined P–E loops are retained at low Er concentrations, the remanent polarization decreases with increasing Er content, and the composition approaches a nearly paraelectric response [171]. In contrast, Nd/Mn co-doping produces one of the most pronounced improvements reported among the systems considered here. Bi_0.85_Nd_0.15_Fe_0.98_Mn_0.02_O_3_ exhibits a well-saturated ferroelectric hysteresis loop with Pr ≈ 31 μC cm^−2^, accompanied by a marked reduction in leakage current; the authors associated this behavior with the reduced concentration of oxygen vacancies and secondary phases together with the accompanying structural transformation [180]. Eu/Ti co-doping also progressively improves the shape of the P–E loops as Ti content increases, consistent with the reduction in leakage current, although the reported remanent polarization remains small (Pr ≤ 0.048 μC cm^−2^) with unsaturated P-E loops [186]. By comparison, Ba/Ta co-doped ceramics do not exhibit conventional ferroelectric hysteresis loops: the low-Ta compositions are strongly affected by leakage, whereas the higher-Ta samples display predominantly lossy-dielectric behavior [172]. Overall, these contrasting trends show that co-substitution can either enhance, preserve, or suppress the ferroelectric response depending on the specific dopant combination and composition, structure, and reduced leakage.

Magnetic enhancement likewise reflects the cooperative nature of simultaneous A- and B-site substitutions. Several co-doped systems exhibit enhanced magnetic response compared with undoped BiFeO_3_ (see Table 6). For example, Eu/Ti co-doping progressively increases the saturation magnetization from 0.219 to 0.547 emu g^−1^ [186]. Similarly, La/Co co-doping increases the remanent magnetization (Mr) from 0.04 to 0.23 emu g^−1^ [178], while Ba/Ta co-doping achieves Mr values approaching 1.5 emu g^−1^, indicating a comparatively large magnetic response among the systems considered here [172]. These representative systems further demonstrate that the magnetic improvements originate from different mechanisms. Specifically, La/Er ceramics exhibit enhanced magnetization associated with structural distortion and modification of the magnetic structure [171]. In contrast, Eu/Ti co-doping combines lattice distortion and defect-related modifications to improve magnetic ordering, whereas Ba/Ta co-doping introduces an additional superparamagnetic contribution together with weak ferromagnetism [172,186]. These results indicate that magnetic behavior is governed by the cooperative interaction between structural distortion, defect chemistry, and magnetic exchange interactions rather than by simple substitution of magnetic ions.

Despite these encouraging results, the multifunctional response remains highly dependent on both the dopant combination and its concentration. Most co-doping systems exhibit an optimum composition, beyond which further substitution offers diminishing returns and may even compromise other functional properties. Consequently, different co-doping strategies should be viewed as complementary approaches for tailoring specific electrical, ferroelectric, or magnetic characteristics according to the intended application rather than as universal solutions for improving all ferroic properties simultaneously. This highlights the importance of rational dopant selection according to the targeted functional response and device application.

**Table 4:** Effect of A-site (Bi-site) doping on the structural, dielectric, ferroelectric, and magnetic properties of BFO.

Dopants with Refs	Sample	Secondary Phase (s)/Crystallite/Particle/Grain Size	Structure/ Fabrication Method	ε’/tanδ @ 1 kHz		ε’/tanδ @ 1 MHz		Ec ~ (kV cm^−1^)	P_r_ ~ (μC cm^−2^)	P_s_ ~ (μC cm^−2^)	Hc ~ (Oe)	M_r_ ~ (emu g^−1^)	M_s_ ~/M_max_@H (emu g^−1^ @ Oe)	
**Ytterbium (Yb)** **[151]**	Bi_1−x_Yb_x_FeO_3_ x = 0	Secondary phases reduced with doping (crystallite 65–49 nm increasing Yb)	*R3c* retained/ sol–gel mediated auto combustion method	100/1.0		<10/<0.2		—	0.93	Unsaturated P–E loops	110	0.06	0.8@10k	AFM + weak FM
	x = 0.1			300/0.2					1.12		156	0.87	4.09@10k	
	x = 0.2			900/0.02					1.42		257	1.76	7.86@10k	FM
**Ytterbium (Yb)** **[152]**	Bi_1−x_Yb_x_FeO_3_ x = 0	Pure phase (crystallite 24–18 nm increasing Yb)	*R3c* retained/ hydrothermal treatment in autoclave	84/0.036		—		—	0.28	Unsaturated P–E loops	186	0.002	0.052@10k	AFM + weak FM
	x = 0.03			94/0.17		—			0.37		45	0.005	0.090@10k	
	x = 0.05			86/0.044		—			0.08		151	0.008	0.047@10k	weak FM
	x = 0.1			150/0.01		—			0.01		127	0.003	0.038@10k	AFM + weak FM
**Ytterbium (Yb)** **[153]**	Bi_1−x_Yb_x_FeO_3_ x = 0	Not reported	Perovskite-type structure and lattice distortion/ rapid liquid-phase sintering method	60/--		55/--			2.1	3.3	—	—	—	
	x = 0.05			77/--		72/--								
	x = 0.1			79/--		74/--								
	x = 0.15			100/--		92/--		50	8.5	11.7				
	x = 0.2			95/--		80/--								
**Lanthanum (La)** **[143]**	Bi_1−x_La_x_FeO_3_ x = 0	Pure phase (grain 1–5 µm undoped, 3–9 µm doped)	*R3c* retained/ solid state reaction method	—		33/0.2–0.4		—	—	—	—	—	AFM	
	x = 0.05			—		57/(2–4) × 10^−3^						~0.008 sintered @ 910 °C, ~0.002 @ 940 °C, ~0.14 @ 950 °C	AFM behavior + weak FM contribution	
**Lanthanum (La)** **[14]**	Bi_1−x_La_x_FeO_3_ x = 0	Secondary phases eliminated	*R3c* retained/ solid state reaction method	—		55–60/0.18		—	—	—	—	—	—	
	x = 0.05			—		65–70/0.02								
**Lanthanum (La)** **[144]**	Bi_1−x_La_x_FeO_3_ x = 0	Minor Bi_2_O_3_ secondary phase (~2%)	Rhombohedral → orthorhombic; transition near x = 0.30/ solid state reaction method	—		—		—	—	Unsaturated P–E loops	20 × 10^3^	0.035	AFM	
	x = 0.1			150/0.2		42/2.3		—	—		40 × 10^3^	0.07	AFM behavior + FM contribution	
	x = 0.2			375/0.6		93/0.007		—	—		40 × 10^3^	0.09		
	x = 0.3			800/0.7		270/0.2		60	9		40 ×10^3^	0.15		
**Cerium (Ce)** **[145]**	Bi_1−x_Ce_x_FeO_3_ x = 0	pure phase (crystallite 76, 27, 26, 17 nm from x = 0 to 0.5)	*R3c* retained/ sol–gel route	52/-		44/-		—	—	—	220	0.09	0.41	
	x = 0.1			13/--		11/--					102	0.22	1.56	
	x = 0.3			26/--		18/--					110	0.49	3.03	
	x = 0.5			23/--		19/--					101	0.18	1.17	
**Cerium (Ce)** **[146]**	Bi_1−x_Ce_x_FeO_3_ x = 0	Secondary phases suppressed at x = 0.15 (grain 5–30 µm for undoped, reduced size with Ce)	*R3c* → cubic/ solid-state reaction method followed by rapid liquid-phase sintering (quenching of the sample after calcination)	950/0.6		40/0.3		—	0.02	Unsaturated P–E loops	AFM			
	x = 0.05			460/0.25		120/0.1			0.07		Weak FM contribute increase from 0 to 0.15 (max Hc = 4000 @ Mr = 0.07) then decrease		AFM behavior + FM contribution	
	x = 0.1			610/0.19		220/0.04			0.2					
	x = 0.15			720/0.06		560/0.04			0.31					
	x = 0.2			270/0.06		160/0.03			0.17					
**Samarium (Sm)** **[147]**	Bi_1−x_Sm_x_FeO_3_ x = 0	Secondary phases eliminated with doping (crystallite 66–44 nm)	*R3c* → orthorhombic (Pbam)/ sol–gel method	19/0.13		17/0.03		—	0.77 × 10^−2^	Unsaturated P–E loops	−100/220	−0.26/0.14	0.67	
	x = 0.05			26/0.11		24/0.02			0.91 × 10^−2^		0/260	−0.33/0	0.84	
	x = 0.1			27/0.1		26/0.01			0.15 × 10^−2^		−10/110	−0.33/−0.04	1.16	
	x = 0.15			31/0.08		28/0.01			0.72 × 10^−2^		−60/350	−0.96/0.18	1.85	
**Samarium (Sm)** **[148]**	Bi_1−x_Sm_x_FeO_3_ x = 0	Pure phase (grain 4–5 µm for undoped, 1–2 µm doped)	Not reported/ solid-state-reaction method	—		—		—	—	—	26.5	3.17 × 10^−4^	AFM	
	x = 0.10										2089	2.2 × 10^−2^	AFM behavior + FM contribution	
**Terbium (Tb)** **[189]**	Bi_1−x_Tb_x_FeO_3_ x = 0	Pure phase (crystallite 15–20 nm, particle ~36 nm)	*R3c* → mixed phases R3c + orthorhombic (*Pnma*)/ sol–gel method	—		—		—	—	—	—	—	AFM	
	x = 0.03										132	1.4	9.7 @12k	AFM + weak FM
	x = 0.04										—	—	—	
	x = 0.06										—	—	—	
	x = 0.09										—	—	—	
	x = 0.12										132	0.4	3.3 @12k	AFM + weak FM
**Neodymium (Nd)** **[150]**	Bi_1−x_Nd_x_FeO_3_ x = 0.08	Pure phase	*R3c* → *Pna2_1_* → *Pbnm*; *R3c/Pna2_1_* coexistence at x ≈ 0.12–0.14 and *Pna2_1_/Pbnm* coexistence at x ≈ 0.22–0.24/ solid-state sintering method	—		—		125	14	15	—	—	AFM behavior + FM contribution	
	x = 0.10							110	33.8	40	3000	10 emu mol^−1^		
	x = 0.12							110	32.2	36	5000	21 emu mol^−1^		
	x = 0.22							—	—	—	3000	91.7 emu mol^−1^		
	x = 0.24							—	1.3	—	5000	75 emu mol^−1^		
**Zinc (Zn)** **[190]**	Bi_1−x_Zn_x_FeO_3_ x = 0	Minor secondary phases	*R3c* retained/ low temperature sol–gel auto-combustion methods	—		250		—	—	—	105	0.019	AFM behavior + weak FM contribution	
	x = 0.05			—		250					152	0.021		
	x = 0.15			—		115					180	0.257		
**Europium (Eu)** **[154]**	Bi_1−x_Eu_x_FeO_3_ x = 0	Pure phase (crystallite 30.45, 37.17, 30.96 nm from x = 0 to 0.3; particle 60.53 nm@ x = 0) and 67.44 nm@x = 0.2)	*R3c* for x ≤ 0.15 → *Pn2_1_a* for x ≥ 0.20/ sol–gel method	127/1.4		—		—	—	—	—	—	AFM	
	x = 0.2			302/1.4							—	0.6	AFM + weak FM 1.8@15k	
	x = 0.3			129/1.5										
**Gadolinium (Gd)** **[149]**	Bi_1−x_Gd_x_FeO_3_ x = 0	Bi_2_Fe_4_O_9_ and Bi_36_Fe_2_O_57_ in BFO; strongly reduced with Gd	*R3c* retained; reduced rhombohedral distortion with increasing Gd/ sol–gel method	40/0.02		40/0.02			0.04	Unsaturated P–E loops	AFM behavior + FM contribution; M from 0.05 (x = 0) to 0.2 (x = 0.15) @1T			
	x = 0.05			60/0.02		60/0.02			0.04					
	x = 0.1			230/0.7		90/0.2			0.04					
	x = 0.15			120/0.4		60/0.04			0.04					
**Potassium (K^+^)** **[191]**	Bi_1−x_K_x_FeO_3_ x = 0	Mostly pure phase (trace of Bi_2_O_3_ and Bi_2_Fe_4_O_9_)	*R3c* retained; evolution toward pseudo-cubic symmetry with increasing K/ flux technique	—		—		—	—	—	AFM behavior retained; magnetization decreases and AFM character is enhanced with increasing K content.			
	x = 0.03													
	x = 0.05													
	x = 0.07													
**Calcium (Ca)** **[157]**	Bi_1−x_Ca_x_FeO_3_ x = 0	Bi_2_O_3_ (particle 114.8, 82.57, and 76.4 nm from x = 0 to x = 0.08)	*R3c* retained; lattice distortion/ sol–gel route	—		—		—	—	—	180	0.88	3.3	
	x = 0.06							2445	0.023	Unsaturated P–E loops	122	0.26	1.6	
	x = 0.08							5654	0.16		83	0.23	2.0	
**Yttrium (y)** **[159]**	Bi_1−x_Y_x_FeO_3_ x = 0	Bi_2_Fe_4_O_9_, Bi_25_FeO_40_ (minor) (particle from 30 to 60 nm increasing Y concentration)	*R3c* (x ≤ 0.20) → orthorhombic (x = 0.25)/ solid-state sintering method	—		—		—	—	—	1738	0.03	0.121 @20k	AFM behavior + weak FM contribution
	x = 0.05										857	0.014	0.129 @20k	
	x = 0.1										957	0.021	0.158 @20k	
	x = 0.15										960	0.034	0.160 @20k	
	x = 0.2										2049	0.040	0.162 @20k	
	x = 0.25										2138	0.111	0.467 @20k	
**Erbium (Er)** **[144]**	Bi_1−x_Er_x_FeO_3_ x = 0	Pure phase	Rhombohedral (x ≤ 0.1) → tetragonal (x ≥ 0.2)/ solid-state sintering route	—		—		Unsaturated/leaky P–E loops			20 × 10^3^	0.04	AFM behavior + FM contribution	
	x = 0.1			190/0.8		50/0.1					44 × 10^3^	0.09		
	x = 0.2			320/0.6		80/0.4					39 × 10^3^	0.06		
	x = 0.3			650/1.2		240/0.1					39 × 10^3^	0.10		
**Holmium (Ho)** **[155]**	Bi_1−x_Ho_x_FeO_3_ x = 0	Pure phase	*R3c* (x ≤ 0.10); Pnma (x ≥ 0.15)/ rapid liquid-phase sintering method	1000	*tanδ* 0.4–1.5	83	*tanδ* 0.3–1	—	—	—	—	2.4 × 10^−3^	AFM 0.2@30k	
	x = 0.05			2900		97					—	7.5 × 10^−3^	0.5 @30k	AFM + weak FM
	x = 0.1			2700		150					—	8.4 × 10^−3^	0.9 @30k	
	x = 0.15			650		100					—	115 × 10^−3^	1.1@30k	
	x = 0.2			750		80					—	117 × 10^−3^	1.3@30k	
**Lead (Pb)** **[158]**	Bi_1−x_Pb_x_FeO_3_ x = 0	pure phase (change particles shape with doping, from sphere to cubic and rods)	Rhombohedral (*R3c*) → tetragonal tendency with increasing Pb content/ chemical combustion	6.2/0.12		5.1/0.01		7.66	0.35	Unsaturated P–E loops	162	1	4.5	
	x = 0.05			1.9/0.05		1.8/0.01		6.03	0.63		191	1.8	8.4	
	x = 0.075			13/0.25		10.6/0.05		8.55	1.57		71	0.2	3	
	x = 0.1			34/0.7		12/0.3		5.06	2.63		187	2.9	9	
**Silver (Ag)** **[192]**	Bi_1−x_Ag_x_FeO_3_ x = 0	Single phase up to x = 0.15; Ag secondary phase above x = 0.15 (crystallite 17.9, 12.7, 8.3 nm (x = 0, 0.05, 0.10); ~75 and ~90 nm length (x = 0.15, 0.20))	*R3c* retained/ glycine-assisted flash auto-combustion	30/0.6		18/0.08		—	—	—	92	0.005	AFM + weak FM 0.33	
	x = 0.05			39/0.5		18/0.08					91.7	0.150	1.19	Enhanced FM component
	x = 0.10			80/0.2		20/0.08					32	0.017	0.83	
	x = 0.15			60/0.5		20/0.08					124.4	0.294	1.95	
	x = 0.20			39/2.0		20/0.08					122.8	0.360	2.43	
**Magnesium (Mg)** **[193]**	Bi_1−x_Mg_x_FeO_3_ x = 0	Secondary phases present (Bi_2_Fe_4_O_9_/Bi_25_FeO_40_) (crystallite 37.44, 28.36, 25.60, 22.80 nm increasing doping)	*R3c* retained; lattice expansion/ solid-state reaction technique	53/0.08		51/0.006		0.98	0.62	Unsaturated P–E loops	—	—	—	
	x = 0.10			148/0.17		115/0.04		0.85	0.75					
	x = 0.15			178/0.33		143/0.2		0.76	0.9					
	x = 0.20			216/0.52		182/0.4		0.66	1.05					
**Dysprosium (Dy)** **[194]**	Bi_1−x_Dy_x_FeO_3_ x = 0 ceramic	Phase improved (reduced Bi_2_Fe_4_O_9_/Bi_25_FeO_40_)	*R3c* retained; peak shift to higher angles/ solid-state reaction technique (bulk) + sonication (nanoparticles)	—		—		7	10	leaky	132	0.001	AFM behavior + weak FM contribution	
	x = 0.10 (ceramic, grain 1 µm)			—		—		14	6.24	9	3793	0.118		
	x = 0.10 (nanoparticles 75–100 nm)			—		—		13	16.98	leaky	1822	0.05		
**Sodium (Na)** **[195]**	Bi_1−x_Na_x_FeO_3_ x = 0	Pure except minor secondary phase of Bi_2_Fe_4_O_9_ in x = 0.05 (particles ~50–60 nm)	*R3c* retained; lattice distortion/ sol–gel method	235/0.17		125/0.15		—	—	—	25	-	AFM behavior + weak FM contribution	
	x = 0.01			225/0.35		100/0.05					6	4 × 10^−3^		
	x = 0.03			150/0.02		150/0.02					1	4.5 × 10^−3^		
	x = 0.05			120/0.03		100/0.02					1.5	4 × 10^−3^		
**Praseodymium (Pr)** **[196]**	Bi_0.9−x_La_0.1_Pr_x_FeO_3_ x = 0	Improved with Pr (impurities eliminated at x = 0.2)	*R3c* retained/ solid-state reaction method	—		—		—	—	—	—	—	AFM behavior	
	x = 0.1			2000/0.6		600/0.4		—	—	—	4373	0.047	AFM behavior + FM contribution	
	x = 0.2			2500/0.4		2000/0.1		—	3.3	Unsaturated P–E loops	7467	0.169		
**Lithium (Li)** **[197]**	Bi_1−x_Li_x_FeO_3_ x = 0	pure phase (undoped particles 152 nm; doped particles ~70 nm)	*R3c* retained/ sol–gel method	—		—		—	—	—	105.39	0.02	0.08	
	x = 0.02										104.24	1.42	4.89	
	x = 0.04										106.91	1.18	4.03	
	x = 0.06										106.73	1.53	5.40	
**Molybdenum (Mo)** **[198]**	Bi_1−x_Mo_x_FeO_3_ x = 0	Bi_2_Fe_4_O_9_ suppressed with doping (crystallite ~21 nm; particle ~0.75, 16, 15, 13 µm increasing dopants)	*R3c* retained; peak shift to higher 2θ (lattice strain)/ sol–gel via auto-combustion method	—		—		—	—	—	164.43	1.829	9.968	
	x = 0.01										156.34	0.540	2.981	
	x = 0.03										154.97	1.307	8.069	
	x = 0.05										156.08	1.128	6.655	
**Strontium (Sr)** **[199]**	Bi_1−x_Sr_x_FeO_3_ x = 0	pure phase (particle 45–90 nm, decreasing with increasing Sr content)	*R3c* → pseudo-tetragonal P4/mmm (x = 0.25)/ tartaric acid mediated sol–gel route	—		—		—	—	—	1300	0.005	0.0099	FM contribution
	x = 0.15										480	0.02	0.085	
	x = 0.25										1100	0.22	0.72	
**Barium (Ba)** **[156]**	Bi_1−x_Ba_x_FeO_3_ x = 0	Pure phase (Bi_2_O_3_/Bi_2_Fe_4_O_9_ eliminated with doping)	Rhombohedral → distorted monoclinic/tetragonal structure/ solid-state reaction method	x = 0.20 max *ε’* @ RT (~90@450 Hz)		—		—	—	—	—	—	AFM behavior	
	x = 0.10					—		0.75	6.25	Unsaturated P–E loops	2500	0.15	0.25	
	x = 0.15					—		0.35	3.1		3700	0.65	1.25	
	x = 0.20					—		0.25	15	20	2800	0.4	0.65	
	x = 0.25					—		0.75	35	leaky P–E	2800	0.2	0.25	

**Table 5:** Effect of B-site doping on the structural, dielectric, ferroelectric, and magnetic properties of BFO.

Dopants with Source	Sample	Secondary Phase (s)/Crystallite/Particle/Grain Size	Structure/ Fabrication Method	(ε’)/tanδ @ 1 kHz	(ε’)/tanδ @ 1 MHz	Ec ~ (kV cm^−1^)	P_r_ ~ (μC cm^−2^)	P_s_ ~ (μC cm^−2^)	Hc ~ (Oe)	M_r_ ~ (emu g^−1^)	M_s_ ~/M_max_@H (emu g^−1^ @ Oe)	
**Dysprosium (Dy)** **[167]**	BiFe_1−x_Dy_x_O_3_ x = 0.05	Pure Phase (crystallite 38 nm, grain ~1.3 µm)	*R3c* → orthorhombic/ solid-state reaction	720/0.31	447/0.23	4.5	0.07	Unsaturated/leaky P–E loops	300	1.45	AFM + weak FM 49@20k	
**Manganese (Mn)** **[161]**	BiFe_1−x_Mn_x_O_3_ x = 0	Pure Phase (nanoparticles)	*R3c* retained; preferred (110) orientation/ organo-metallic sol–gel	14.9/0.5	10/1	—	—	—	69	9.11 × 10^−9^	0.077 @15k	AFM behavior + weak FM contribution
	x = 0.1			28.7/2	10/1				280	5.5 × 10^−3^	0.12 @15k	
	x = 0.2			221.6/9	50/1				127	2.9 × 10^−3^	0.17 @15k	
**Zirconium (Zr^4+^)** **[166]**	Bi_X_Fe_0.9_Zr_0.1_O_3_ x = 1	Pure Phase	*R3c* Retained/ solid-state reaction	—	—	FE properties are believed to remain unchanged			—	—	—	
	x = 0.975								—	—	—	
	x = 0.95								6300	0.05	AFM behavior + FM contribution	
**Vanadium (V^5+^)** **[163]**	BiFe_1−x_V_x_O_3_ x = 0	Bi_2_O_3_	*R3c* retained; lattice distortion/ rapid liquid-phase sintering			20	1.25	Unsaturated/leaky P-E loop	—	—	—	
	x = 0.03			44/0.05	25/0.7	48	0.9					
**Titanium (Ti)** **[162]**	BiFe_1−x_Ti_x_O_3_ x = 0.25	Bi_46_Fe_2_O_72_ and Bi_2_Fe_4_O_9_ (grain ~1 µm)	*R3c* retained; lattice distortion/ solid-state reaction	300/0.14	250/0.03	2571	0.081	Unsaturated P-E loop	AFM behavior + weak FM contribution			
**Holmium (Ho)** **[118]**	BiHo_X_Fe_1−X_O_3_ x = 0	Pure Phase (Crystallite 45.8, 37.51, 30.60, 18 nm increasing Ho content)	*R3c* retained/ solid-state reaction	15/0.18	30/0.1	Unsaturated/leaky P-E loop			AFM behavior			
	x = 0.05			66/0.2	30/0.12	74	4.2	7				
	x = 0.1			157/0.3	30/0.14	36	5.4	5.3	AFM behavior + weak FM contribution			
	x = 0.15			240/0.4	30/0.15	74	8.4	13.4	—	0.0258	0.364	
**Tantalum (Ta)** **[164]**	BiFe_1−x_Ta_x_O_3+x_ *x* = 0	Improved (Bi_25_FeO_40_/Bi_2_Fe_4_O_12_ reduced) (grain ~50 µm for BFO; <1 µm for BFTO)	*R*3*c* retained/ solid-state reaction	—	—	—	—	Unsaturated P-E loop	—	—	AFM behavior + FM contribution	
	x = 0.01			125/-- (10 kHz)	120/-- (100 kHz)		0.22		6200	0.06		
	x = 0.03			100/--	--		0.15		6200	0.06		
**Zinc (Zn)** **[165]**	BiFe_1−x_Zn_x_O_3_ *x* = 0	Pure phase up to x = 0.025; Bi_2_Fe_4_O_9_/Bi_25_FeO_40_ at x = 0.05 (grain ~28 µm for x = 0 and 0.025; ~11 µm for x = 0.05 and 0.1)	*R*3*c* retained/ hydrothermal method	—	—	—	—	—	—	0.7 × 10^−3^	AFM behavior	
	x = 0.025									3 × 10^−3^		
	x = 0.05									0.7 × 10^−3^	AFM behavior + weak FM contribution	
	x = 0.1									0.25 × 10^−3^		
**Nickel (Ni)** **[169]**	BiFe_1−x_Ni_x_O_3_ *x* = 0	Minor secondary phases	*R*3*c* retained/ solid-state reaction	—	—	—	—	—	—	0.006	AFM behavior + weak FM contribution	
	*x* = 0.05									0.10	AFM + FM 0.41	
**Cobalt (Co)** **[168]**	BiFe_1−x_Co_x_O_3_ x = 0	Pure Phase	transition from rhombohedral (0 ≤ x ≤ 0.2) to a monoclinic (x = 0.3) and to a tetragonal BiCoO_3_-type (0.5 ≤ x ≤ 0.6)/ high-pressure solid-state synthesis	—	—	—	—	—	—	—	AFM	
	*x* = 0.1								—	0.025	AFM behavior + FM contribution	
	*x* = 0.2								—	0.025		
	*x* = 0.3								—	—	AFM	
	*x* = 0.5								—	—	AFM	
**Tin (Sn)** **[200]**	BiFe_1−x_Sn_x_O_3_ x = 0	Bi_2_Fe_4_O_9_/Bi_2_O_3_; lowest impurity at x = 0.15 (particle 64–40 nm increasing % Sn)	rhombohedral → orthorhombic/ sol–gel method	—	—	—	—	—	—	—	—	
	*x* = 0.05								168	0.132	0.491	
	*x* = 0.10								195	0.203	0.808	
	*x* = 0.15								235	0.304	1.151	
	*x* = 0.25								192	0.212	0.706	
**Selenium (Se)** **[201]**	BiFe_1−x_Se_x_O_3_ x = 0	No clear improvement; Se-related peak (~30°) at higher doping (nanostructures with morphological changes increasing Se-concentration)	rhombohedral → distorted orthorhombic/double-solvent sol–gel method	—	—	—	—	—	AFM + weak FM			
	*x* = 0.1								410	0.013	0.0390	
	*x* = 0.25								270	0.034	0.0990	
	*x* = 0.5								341	0.0072	0.0207	
	*x* = 1								250	0.0005	0.0025	
**Aluminum (Al)** **[202]**	BiFe_1−x_Al_x_O_3_ x = 0	Single phase ≤ 0.5 mol%; Bi_2_Fe_4_O_9_/Bi_25_FeO_39_ at higher Al (grain 1.6 → 0.45 μm with increasing Al)	*R3c* retained/ solid-state reaction	400/0.67	50/0.07	14	0.15	Unsaturated/leaky P-E loop	—	—	—	
	*x* = 0.005			355/0.65	50/0.045	16	0.42					
	*x* = 0.01			350/0.47	50/0.07	18	0.22					
	*x* = 0.015			80/0.25	50/0.003	—	—					
	*x* = 0.02			120/0.65	50/0.015	—	—					
**Chromium (Cr)** **[203]**	BiFe_1−x_Cr_x_O_3_ x = 0	Pure Phase (particle ~10–100 nm pure BFO; ~50 nm Cr-doped)	*R3c* retained/ hydrothermal synthesis	—	—	—	—	—	AFM			
	*x* = 0.05								AFM behavior + weak FM contribution			
	*x* = 0.10											
	*x* = 0.15											
	*x* = 0.20											
**Indium (In)** **[204]**	BiFe_1−x_In_x_O_3_ x = 0	Reduced BFO fraction (85.8%) with In (23.3%) (grain 2.04 → 0.23 μm with increasing In)	*R3c* → *R3c* + BiInO_3_ + Bi_25_FeO_40_ (x ≥ 0.1); secondary phases become dominant at x = 0.5–0.6/ solid-state reaction	26.5/3.52	17/0.1	—	—	—	93.13	5.16 × 10^−4^	0.035@10k	AFM behavior + weak FM contribution
	*x* = 0.1			54.2/12.57	16/0.1				23.93	2.72 × 10^−4^	0.040@10k	
	*x* = 0.2			140/7.94	15/0.1				201.1	5.20 × 10^−4^	0.007@10k	
	*x* = 0.3			199/4.74	9.6/0.3				85.91	6.22 × 10^−4^	0.040@10k	
	*x* = 0.4			255/2.59	10/0.3				32.90	1.76 × 10^−4^	0.026@10k	
	*x* = 0.5			256/1.42	9.8/0.2				82.50	2.30 × 10^−4^	0.010@10k	
	*x* = 0.6			372/0.59	9.6/0.7				54.70	4.25 × 10^−4^	0.030@10k	

**Table 6:** Effect of co-doping on the structural, dielectric, ferroelectric, and magnetic properties of BFO.

Dopants with Source	Sample	Secondary Phase(s)/Crystallite/Particle/Grain Size	Structure/ Fabrication Method	(ε’)/Loss @ 1 kHz	(ε’)/Loss @ 1 MHz	Ec ~ (kV cm^−1^)	P_r_ ~ (μC cm^−2^)	P_s_ ~ (μC cm^−2^)	Hc ~ (Oe)	M_r_ ~ (emu g^−1^)	M_s_ ~/M_max_@H (emu g^−1^ @ Oe)	
**Lanthanum (La) and Erbium (Er)** **[171]**	Bi_0.9–x_ La_0.1_Er_x_ FeO_3_ x = 0	Pure phase	*R3c* → monoclinic C2(*x* = 0.05); mixed tetragonal/rhombohedral (*x* = 0.07)/ solid-state reaction	~68/0.015	70/0.01	60	11	18.4	AFM			
	*x* = 0.05			~52/0.05	50/0.02	75	9	16.3	—	0⋅076	AFM behavior + weak FM contribution	
	*x* = 0.07			~43/0.05	40/0.028	90	7.5	13.4	—	0⋅1164		
**Lanthanum (La) and Vanadium (V).** **[163]**	*x* = 0.1	Bi_2_O_3_	*R3c* retained/ rapid liquid-phase sintering	~45/0.09	40/0.1	40	1.5	Unsaturated P-E loop	—	—	—	
**Barium (Ba) and Tantalum (Ta)** **[172]**	Bi_0.8_Ba_0.2_Fe_1−x_Ta_x_O_3_ x = 0	Bi_25_FeO_39_ (x = 0–0.10); Bi_3_TaO_7_ + pyrochlore (x = 0.15) (grain 0.11 → 0.23 μm with increasing Ta)	Tetragonal *P4mm* (x = 0–0.10); *P4mm/R3c* coexistence (x = 0.15)/solid-state reaction	140/0.3	110/0.05	9	8	—	1300	0.206	0.55	FM component
	x = 0.05			222/0.4	130/0.07	8.5	39		1090	0.101	0.297	
	x = 0.1			250/0.12	206/0.085	2.5	0.19		190	0.07	0.22	
	x = 0.15			284/0.08	182/0.093	1	0.05		100	0.002	0.02	
**Zirconium (Zr) and Copper (Cu)** **[205]**	Bi_1−x_ Zr_x_Fe_0.98_Cu_0.02_O_3_ *x* = 0	Pure phase (nanoparticles)	*R3c* retained/ sol–gel method	—	8.13/1454	—	—	—	—	—	—	
	*x* = 0.01			—	6.79/634							
	*x* = 0.02				12.63/596							
	*x* = 0.03				6398/0.71							
	*x* = 0.04				6942/0.57							
	*x* = 0.05				8702/0.39							
**Holmium (Ho) and Titanium (Ti)** **[185]**	Bi_0.9_Ho_0.1_Fe_0.95_O_3_	Bi_2_Fe_4_O_9_ (grain < 1 μm)	*R3c* retained/ solid-state reaction	950/0.6	150/0.4	—	—	—	—	0.024	AFM behavior + weak FM contribution	
	Bi_0.9_Ho_0.1_Fe_0.9_Ti_0.05_O_3_			168/0.02	150/0.04	20	0.4	Unsaturated P-E loop		0.043		
**Neodymium (Nd) and Manganese (Mn)** **[180]**	Bi_1−x_Nd_x_Fe_1−y_Mn_y_O_3_ *x* = 0, y = 0	Pure Phase (grain ~ 1–5 μm)	*R3c* → *P4mm/* rapid liquid-phase sintering	50/0.2	45/0.112	—	—	Leaky PE loop	AFM			
	*x* = 0.15, y = 0.02			170/0.05	160/0.008	110	31	40	5800	0.075	AFM behavior + FM contribution	
**Lanthanum (La) and Nickel (Ni)** **[173]**	Bi_1−x_La_x_Fe_1−y_NiyO_3_ *x* = 0.2, y = 0	Pure Phase (particle 100–150 nm for BFO; 50−60 nm for x, y = 0.2)	*R3c* → Orthorhombic (*Pbnm*)/ ethylene-glycol-based sol–gel	70/0.16	50/0.03	—	—	—			0.14	
	*x* = 0.2, y = 0.1			90/0.25	60/0.08				—	—	0.3	
	*x* = 0.2, y = 0.2			85/0.075	61/0.015						0.16	
**Calcium (Ca) and Neobium (Nb)** **[174]**	Bi_0.82_Ca_0.18_Fe_0.91_Nb_0.09_O_3_	Pure Phase	R3c retained/ solid-state reaction	—	—	—	—	—	—	—	0.3	
**Samarium (Sm) and Scandium (Sc)** **[175]**	(1−x)Bi_1−y_Sm_y_FeO_3_-xBiScO_3_ *x* = 0.05, y = 0	Pure Phase	*R3c* → orthorhombic (*Pnma*) at y = 0.15/rapid thermal quenching technique	64/0.02 @ 10 kHz		53.1	14.2	—	—	—	—	
	*x* = y = 0.05			70/0.002 @ 10 kHz		50–55	5–5.5	—				
	*x* = 0, y = 0.05			65/0.002 @ 10 kHz		50–55	7	—				
	*x* = 0.75, y = 0.05			75/0.02 @ 10 kHz		60–65	6.5	—				
**Yttrium (Y) and Nickel (Ni)** **[176]**	Bi_0.99_Y_0.01_Fe_1−x_Ni_x_O_3_ BFO, *x* = 0	Fe_3_O_4_ secondary phase (crystallite 218–94 nm increasing Ni)	*R3c* retained/ solid-state reaction method	—	—	—	—	—	AFM			
	BYFNO-2, *x* = 0.02								138	0.22	1.05	AFM behavior + FM contribution
	BYFNO-5, *x* = 0.05								—	—	—	
**Neodymium (Nd) and Zinc (Zn)** **[177]**	Bi1-xNdxFe_0.975_Zn_0.025_O_3_ x = 0	Pure Phase (grain 200–60 nm increasing Nd)	*R3c* retained/ sol–gel method	—	—	—	—	—	AFM			
	x = 0.025											
	x = 0.05											
	x = 0.075								1000	0.03	AFM behavior + FM contribution	
	x = 0.1								7000	0.105		
**Lanthanum (La) and Cobalt (Co)** **[178]**	La_0.1_Bi_0.9_Fe_1−*x*_Co*_x_*O_3_ *x* = 0	Pure Phase (grain size decreases with Co)	Not reported/ modified rapid sintering process	75.1/0.017	70/0.11	—	—	—	3800	0.04	AFM behavior + FM contribution	
	x = 0.01			30/0.1	28/0.12				960	0.08		
	x = 0.03			30/0.25	27/0.15				850	0.23		
**Zinc (Zn) and Nickel (Ni)** **[179]**	Bi_1−x_Zn_x_Fe_1−y_Ni_y_O_3_ x = y = 0.025	Bi_12_(Bi_0_._5_Fe_0_._5_)O_19_._5_ and Bi_12_NiO_19_ impurities (crystallite 19–47 nm)	*R3c* retained/ solution combustion method	36/0.13	28/0.03	—	—	Unsaturated P-E loop	—	—	AFM behavior + FM contribution	
	y = 0.05, x = 0.05			31/0.12	22/0.02							
	y = 0.075, x = 0.075			54/0.2	32/0.03							
	y = 0.1, x = 0.1			70/0.3	32/0.06							
**Lanthanum (La) and Titanium (Ti)** **[206]**	Bi_0.85_La_0.15_Fe_0.9_Ti_0.1_O_3_	Pure Phase	Tetragonal/ Rapid liquid-phase sintering	300/0.1	200/0.07	—	—	—	—	—	AFM behavior + FM contribution	
**Lead (Pb) and Titanium (Ti)** **[207]**	Bi_0.9_Pb_0.1_Fe_1−x_Ti_x_O_3_ x = 0.05	Bi_2_Fe_4_O_9_, Fe_2_O_3_, PbO, PbTiO_3_ (traces) (crystallite 62–92 nm; grain 1.6–1.4 μm)	*R3c* + *P4mm*/ solid-state reaction	~70/0.35	~10/~ 0.01	—	—	—	204	0.007	AFM behavior + FM contribution	
	x = 0.10			~70/0.35					195	0.017		
	x = 0.15			~70/0.27					170	0.008		
	x = 0.20			~70/0.18					104	0.020		
**Barium (Ba) and Titanium (Ti)** **[208]**	Bi_0.9_Ba_0.1_Fe_0.925_Ti*_x_*O_3_ *x* = 0.0625	Bi_46_Fe_2_O_72_ and Bi_2_Fe_4_O_9_	*R3c* retained; subtle lattice distortions/ two-step doping	350/0.1	210/0.2	—	—	—	3500	1.25	AFM behavior + FM contribution	
	*x* = 0.08125			370/0.05	220/0.2				3000	1.4		
	*x* = 0.0875			210/0.05	200/0.15				3500	1.45		
	*x* = 0.11			225/0.04	190/0.14				4000	1.5		
**Europium (Eu) and Titanium (Ti)** **[186]**	Bi_0.95_Eu_0.05_Fe_1−x_Ti_x_O_3_ x = 0	Improved (Bi_24_FeO_39_/Bi_2_Fe_4_O_9_ eliminated after doping) (grain size decreases with Ti)	*R3c* → structural phase transition (x ≥ 0.10)/solid-state reaction method followed by rapid liquid-phase sintering	650/0.85	20/0.1	—	—	Unsaturated P-E loop	—	AFM		
	x = 0.05			400/0.45	100/0.08		0.048			0.036	0.219	Week FM behavior
	x = 0.10			330/0.3	170/0.07		0.17			0.047	0.381	
	x = 0.15			330/0.19	320/0.07		0.28			0.075	0.547	
**Zinc (Zn) and Titanium (Ti)** **[188]**	BiFe_1−2x_Zn_x_Ti_x_O_3_ x = 0	Bi_25_FeO_39_	*R3c* → tetragonal-like (MPB)/ sol–gel followed by conventional sintering	600/0.7	40/	—	—	Unsaturated/leaky P-E loop	AFM behavior			
	x = 0.005			1300/0.7	75/-	—	—					
	x = 0.01			1100/0.3	65/-	—	—					
	x = 0.015			300/0.25	45/-	—	—					
	x = 0.02			250/0.2	85/-	7.5	0.31					
	x = 0.025			100/0.1	85/-	15.5	0.4					
**Neodymium (Nd) and Niobium (Nb)** **[187]**	BiFeO_3_	Pure phase (grain from 2.16 μm to <30 μm increasing Nd)	*R3c* → *R3c* + *Pnma*/ sol–gel synthesis of the precursor nanopowders + spark plasma sintering	Not reported	197/0.7	4.45	0.41	Unsaturated P-E loop	—	5.67 × 10^−4^	AFM	
	Bi_1−x_Nd_x_Fe_0.95_Nb_0.05_O_3_ x = 0.05				206/0.28	11.40	1.86		6170	0.07	AFM behavior + FM contribution	
	x = 0.10				233/0.28	11.10	3.12		8840	0.12		
	x = 0.15				247/0.25	6.13	0.51		9800	0.15		
	x = 0.20				150/0.2	6.36	0.36		9050	0.14		
**Samarium (Sm) and Cobalt (Co)** **[184]**	BiFeO_3_	Minor secondary phase, increase with dopant % (crystallite 66 to 23 nm increasing dopant concentration)	*R3c* → *Pnma*/ sol–gel method	—	—	1.54	0.76	Unsaturated P-E loop	−110/210	−0.25/0.13	0.66	
	Bi_0.9_Sm_0.1_Fe_1−x_Co_x_O_3_ x = 0.05					3.30	3.78		−640/960	−1.16/0.88	2.13	
	x = 0.10					2.91	2.88		−590/710	−1.41/1.19	2.94	
	x = 0.15					5.64	7.21		−480/820	−1.68/1.24	3.20	

## 5. Conclusions and Future Perspectives

### 5.1. Critical Assessment of Chemical Substitution Strategies in BiFeO_3_

A plethora of studies investigated strategies for tailoring the electrical and magnetic properties of BiFeO_3._ One such route is nanostructuring, whereby size reduction and the high surface-to-volume ratio of nanoparticles can modify the magnetic response. In particular, particle dimensions approaching or below the characteristic spin-cycloid period (~62 nm) can contribute to enhanced magnetization through suppression or modification of the cycloidal spin structure. Despite these potential advantages, the available evidence highlights several important limitations, such as impurity phases and high leakage in BiFeO_3_ nanoparticles. The literature shows considerable variability in synthesis methods, calcination and sintering conditions, precursor chemistry, grain size, density, oxygen-vacancy concentration, and secondary-phase formation, all of which strongly influence the measured functional properties. Consequently, nominally identical compositions frequently exhibit substantially different electrical and magnetic responses, preventing meaningful comparison between independent studies and often obscuring the intrinsic influence of chemical substitutions.

It is evident that strategic site-selective doping can tailor the magnetic and electrical properties of both ceramics and nanoparticles. The enhanced functional properties rarely originate from the chemical identity of the dopant alone. Instead, they result from a complex interaction between lattice distortion, defect compensation, suppression of oxygen vacancies, modification of the Fe–O–Fe superexchange interaction, grain growth, phase evolution, and partial suppression of the cycloidal spin structure. Consequently, it is difficult to identify a universally superior dopant, since different compositions often achieve comparable performance through similar structural and defect-mediated mechanisms. However, systematic A-site substitution offers distinct benefits, as it modifies the structure and controls the volatility of Bi ions, thus improving the microstructure and decreasing the secondary phases, leading to enhanced electric and magnetic behavior. A-site doping with an ion of similar valence could lead to a structural transition with an increase in doping from rhombohedral to orthorhombic to tetragonal. The instability created by the A-site doping can also influence the magnetic exchange interactions and the resulting magnetic response. Overall improvement in microstructure and phase purity of the sample enhances the ferroelectric and magnetic properties. On the other hand, aliovalent doping at the A-site could enhance magnetic behavior; however, charge compensation could create oxygen vacancies or variable Fe valence states.

Similarly, B-site substitution more directly modifies the FeO_6_ octahedral network and Fe–O–Fe superexchange interactions. Aliovalent substitution can promote spin canting and destabilization of the cycloidal spin structure, thereby enhancing the magnetic response; however, the associated charge-compensation mechanisms may also increase oxygen-vacancy concentration and leakage current, adversely affecting the electrical properties. Isovalent B-site substitution, such as Cr^3+^ for Fe^3+^, can instead modify the magnetic structure while limiting the need for charge compensation and the formation of oxygen vacancies. Appropriate Cr^3+^ concentrations have been associated with reduced secondary-phase formation and the emergence of weak ferromagnetism through modification of the antiferromagnetic spin structure.

Co-doping at A- and B-sites is predicted to enhance the ferroelectric and ferromagnetic properties. However, its effectiveness strongly depends on the appropriate selection and concentration of the substituting ions, since improvements in density and phase stability may coexist with defect-related contributions, such as oxygen vacancies, that adversely affect the ferroelectric behavior of BFO. The evidence discussed throughout this review further indicates that the multifunctional nature of BiFeO_3_ remains insufficiently explored. Most experimental studies remain focused on improving individual properties, such as polarization, dielectric constant, leakage current, or magnetization, whereas comprehensive investigations combining structural characterization with ferroelectric, magnetic, dielectric, and magnetoelectric measurements under identical experimental conditions remain comparatively scarce. This fragmented approach limits a complete understanding of the coupling mechanisms governing the multifunctional response and complicates the interpretation and cross-comparison of the reported improvements.

Finally, although numerous studies have successfully induced rhombohedral-to-orthorhombic, rhombohedral-to-tetragonal, or mixed-phase structures through chemical substitution, the precise relationship between structural phase evolution and multifunctional properties remains poorly understood. In particular, the role of morphotropic or polymorphic phase boundaries in enhancing polarization, piezoelectricity, magnetic ordering, and magnetoelectric coupling has not yet been systematically established for bulk BiFeO_3_ ceramics. Establishing these structure–property relationships represents one of the most important scientific challenges for the field.

### 5.2. Future Perspectives

Future research should move beyond empirical compositional optimization toward mechanism-driven materials design. Particular emphasis should be placed on establishing predictive structure–processing–defect–property relationships and on understanding how these interconnected factors influence ferroic coupling. Such knowledge would enable the rational selection of dopants capable of simultaneously optimizing ferroelectric, magnetic, and magnetoelectric properties.

Improving reproducibility should also become a major priority. Standardized synthesis protocols, processing conditions, and characterization methodologies are essential for reducing the significant variability currently observed between independent studies. More consistent reporting of grain size, density, secondary phases, oxygen-vacancy concentration, leakage current, and experimental measurement conditions would greatly facilitate meaningful comparison among published results and accelerate the development of reliable structure–property relationships.

Another promising direction involves a more systematic investigation of structural phase transitions, space-group symmetry, and phase coexistence. While numerous studies have reported enhanced functional responses near rhombohedral, orthorhombic, tetragonal, and mixed-phase regions, the mechanisms responsible for these improvements remain incompletely understood. Recent work has emphasized that space-group symmetry itself can serve as an important framework for understanding the relationship between structural distortions and the coupled ferroic and energy-related functionalities of BiFeO_3_. Future work should clarify whether morphotropic or polymorphic phase boundaries can be reliably engineered in bulk BiFeO_3_ through controlled chemical substitution and processing.

A particularly important challenge is achieving robust room-temperature magnetoelectric coupling. Although many studies report enhanced magnetization or improved ferroelectric behavior following chemical substitution, direct measurements of magnetoelectric coupling remain comparatively limited. Future investigations should combine advanced structural characterization with in situ electric and magnetic field measurements to establish quantitative relationships between spin cycloid suppression, magnetic exchange interactions, structural distortion, and the resulting magnetoelectric response. Particular attention should also be devoted to distinguishing intrinsic magnetoelectric coupling from extrinsic contributions and to standardizing measurement conditions to enable meaningful comparison of reported α_ME_ values.

BiFeO_3_ is an adaptive functional material where versatility is enabled through composition, and microstructure and structural engineering make it suitable for various applications. In ceramics, composition-induced changes in domain configuration and structural state can redirect the ferroelectric response toward functionalities such as capacitive energy storage [209]. With scalability, control over size, morphology, interfaces, and polarization provides additional degrees of freedom and can enable functionalities such as polarization-controlled ionic transport [210]. Such exploration in breadth of functionality portrays BiFeO_3_ as a chameleon where internal properties can be selectively reconfigured from ceramic energy storage devices to technologically scalable biomedical devices, sensors, and photocatalytic activities.

Ultimately, the future of bulk BiFeO_3_ lies not in identifying another high-performing dopant, but in developing predictive design principles that quantitatively relate composition, processing, crystal structure, defect chemistry, and coupled ferroic responses. This transition from empirical optimization to predictive, knowledge-driven materials engineering will lay the foundation for the next generation of high-performance, room-temperature multiferroic ceramics.

## Data Availability

No new data were created or analyzed in this study. Data sharing is not applicable to this article.

## References

[1] Schmid H. (1994). Multi-ferroic magnetoelectrics. Ferroelectrics.

[2] Fiebig M. (2005). Revival of the magnetoelectric effect. J. Phys. D. Appl. Phys..

[3] Eerenstein W., Mathur N.D., Scott J.F. (2006). Multiferroic and magnetoelectric materials. Nature.

[4] Jie W., Hao J. (2014). Graphene-based hybrid structures combined with functional materials of ferroelectrics and semiconductors. Nanoscale.

[5] Jiang Q., Nan C., Wang Y., Liu Y., Shen Z. (2007). Synthesis and properties of multiferroic BiFeO_3_ ceramics. J. Electroceramics.

[6] Rojac T., Bencan A., Malic B., Tutuncu G., Jones J.L., Daniels J.E., Damjanovic D. (2014). BiFeO_3_ ceramics: Processing, electrical, and electromechanical properties. J. Am. Ceram. Soc..

[7] Lebeugle D., Colson D., Forget A., Viret M., Bataille A.M., Gukasov A. (2008). Electric-field-induced spin-flop in BiFeO_3_ single crystals at room-temperature. Phys. Rev. Lett..

[8] Liu Y., Wang Y., Ma J., Li S., Pan H., Nan C.W., Lin Y.H. (2022). Controllable electrical, magnetoelectric and optical properties of BiFeO_3_ via domain engineering. Prog. Mater. Sci..

[9] Yang J.-C., He Q., Yu P., Chu Y.-H. (2015). BiFeO_3_ Thin Films: A Playground for Exploring Electric-Field Control of Multifunctionalities. Annu. Rev. Mater. Res..

[10] Zhao T., Scholl A., Zavaliche F., Lee K., Barry M., Doran A., Cruz M.P., Chu Y.H., Ederer C., Spaldin N.A. (2006). Electrical control of antiferromagnetic domains in multiferroic BiFeO_3_ films at room temperature. Nat. Mater..

[11] Spaldin N.A. (2019). Advances in magnetoelectric multiferroics. Nat. Mater..

[12] You L., Wang J. (2018). Multiferroism and Magnetoelectric Applications in Bismuth Ferrite. Functional Materials and Electronics.

[13] Srihari N.V., Vinayakumar K.B., Nagaraja K.K. (2020). Magnetoelectric coupling in Bismuth Ferrite—Challenges and perspectives. Coatings.

[14] Tu C.S., Ding Y., Yang W.C., Wang T.H., Chien R.R., Schmidt V.H., Yao Y.D., Wu K.T. (2011). Dielectric permittivity and magnetoelectric coupling in multiferroic BiFeO_3_ and (Bi_0.95_La_0.05_)FeO_3_ ceramics. IEEE Trans. Magn..

[15] Kumar M., Sati P.C., Kumar A., Sahni M., Negi P., Singh H., Chauhan S., Chaurasia S.K. (2021). Recent advances on magnetoelectric coupling in BiFeO_3_: Technological achievements and challenges. Mater. Today Proc..

[16] Wu J., Fan Z., Xiao D., Zhu J., Wang J. (2016). Multiferroic bismuth ferrite-based materials for multifunctional applications: Ceramic bulks, thin films and nanostructures. Prog. Mater. Sci..

[17] Jain A., Wang Y.G., Kumar A., Gupta N., Kumar K., Goyal A.K. (2025). BiFeO_3_-based lead-free materials: Recent breakthroughs and their multifunctional applications. J. Alloys Compd..

[18] Sharma S., Mahadevan S., Pandey K., Singh P., Chauhan S., Kumar M., Siqueiros J.M., Herrera O.R. (2025). Phase transition driven enhancements of multiferroic properties in doped BiFeO_3_: A comprehensive review. Mater. Sci. Semicond. Process..

[19] Wadhawan V.K. (2002). Ferroic Materials: A Primer. Resonance.

[20] Zheng Y., Chen W.J., Schmid H. (2008). Some symmetry aspects of ferroics and single phase multiferroics*. J. Phys. Condens. Matter.

[21] Chu Y.-H., Martin L.W., Holcomb M.B., Gajek M., Han S.-J., He Q., Balke N., Yang C.-H., Lee D., Hu W. (2008). Electric-field control of local ferromagnetism using a magnetoelectric multiferroic. Nat. Mater..

[22] Bibes M., Barthélémy A. (2008). Towards a magnetoelectric memory. Nat. Mater..

[23] Bichurin M., Petrov R., Leontiev V., Semenov G., Sokolov O. (2017). Magnetoelectric current sensors. Sensors.

[24] Rivera J.-P. (2011). On definitions, units, measurements, tensor forms of the linear magnetoelectric effect and on a new dynamic method applied to Cr-Cl boracite. Ferroelectrics.

[25] Schmid H. (2011). Introduction to the proceedings of the 2nd international conference on magnetoelectric interaction phenomena in crystals, MEIPIC-2. Ferroelectrics.

[26] Debye P. (1926). Bemerkung zu einigen neuen Versuchen über einen magneto-elektrischen Richteffekt. Z. Für Phys..

[27] Dzyaloshinskiǐ I. (1960). On the magneto-electrical effects in antiferromagnets. Sov. Phys. JETP.

[28] Astrov D.N. (1960). The Magnetoelectric Effect in Antiferromagnetics. J. Exptl. Theor. Phys..

[29] Rado G.T., Folen V.J. (1961). Observation of the Magnetically Induced Magnetoelectric Effect and Evidence for Antiferromagnetic Domains. Phys. Rev. Lett..

[30] Folen V.J., Rado G.T., Stalder E.W. (1961). Anisotropy of the Magnetoelectric Effect in Cr_2_O_3_. Phys. Rev. Lett..

[31] Smolenskiǐ G.A., Agranovskaya A. (1958). Dielectric Polarization and Losses of Some Complex Compounds. Zhur. Tekh. Fiz..

[32] Wang J., Neaton J.B., Zheng H., Nagarajan V., Ogale S.B., Liu B., Viehland D., Vaithyanathan V., Schlom D.G., Waghmare U.V. (2003). Epitaxial BiFeO_3_ multiferroic thin film heterostructures. Science.

[33] Kimura T., Lawes G., Goto T., Tokura Y., Ramirez A.P. (2005). Magnetoelectric phase diagrams of orthorhombic RMnO_3_ (R=Gd, Tb, and Dy). Phys. Rev. B Condens. Matter Mater. Phys..

[34] Hur N., Park S., Sharma P.A., Ahn J.S., Guha S., Cheong S.W. (2004). Electric polarization reversal and memory in a multiferroic material induced by magnetic fields. Nature.

[35] Lawes G., Harris A.B., Kimura T., Rogado N., Cava R.J., Aharony A., Entin-Wohlman O., Yildirim T., Kenzelmann M., Broholm C. (2005). Magnetically driven ferroelectric order in Ni_3_V_2_O_8_. Phys. Rev. Lett..

[36] Khomskii D. (2009). Classifying multiferroics: Mechanisms and effects. Physics.

[37] Van Aken B.B., Palstra T.T.M., Filippetti A., Spaldin N.A. (2004). The origin of ferroelectricity in magnetoelectric YMnO_3_. Nat. Mater..

[38] Kubel F., Schmid H. (1990). Structure of a ferroelectric and ferroelastic monodomain crystal of the perovskite BiFeO_3_. Acta Crystallogr. Sect. B.

[39] Belik A.A. (2012). Polar and nonpolar phases of BiMO_3_: A review. J. Solid State Chem..

[40] Lee J.H., Fang L., Vlahos E., Ke X., Jung Y.W., Kourkoutis L.F., Kim J.W., Ryan P.J., Heeg T., Roeckerath M. (2010). A strong ferroelectric ferromagnet created by means of spin-lattice coupling. Nature.

[41] Ederer C., Spaldin N.A. (2006). Origin of ferroelectricity in the multiferroic barium fluorides BaMF_4_: A first principles study. Phys. Rev. B Condens. Matter Mater. Phys..

[42] Scott J.F., Blinc R. (2011). Multiferroic magnetoelectric fluorides: Why are there so many magnetic ferroelectrics?. J. Phys. Condens. Matter.

[43] Garcia-Castro A.C., Ibarra-Hernandez W., Bousquet E., Romero A.H. (2018). Direct Magnetization-Polarization Coupling in BaCuF_4_. Phys. Rev. Lett..

[44] Iwata N., Kohn K. (1998). Dielectric anomalies at magnetic transitions of hexagonal rare earth manganese oxides RMnO_3_. J. Phys. Soc. Jpn..

[45] Aoyama T., Iyama A., Shimizu K., Kimura T. (2015). Multiferroicity in orthorhombic RMnO_3_ (R = Dy, Tb, and Gd) under high pressure. Phys. Rev. B Condens. Matter Mater. Phys..

[46] Xu X., Wang W. (2014). Multiferroic hexagonal ferrites (h-RFeO_3_, R = Y, Dy-Lu): A brief experimental review. Mod. Phys. Lett. B.

[47] Wang W., Zhao J., Wang W., Gai Z., Balke N., Chi M., Lee H.N., Tian W., Zhu L., Cheng X. (2013). Room-temperature multiferroic hexagonal LuFeO_3_ films. Phys. Rev. Lett..

[48] Warshi M.K., Mishra V., Sagdeo A., Mishra V., Kumar R., Sagdeo P.R. (2018). Structural, optical and electronic properties of RFeO_3_. Ceram. Int..

[49] Disseler S.M., Luo X., Gao B., Oh Y.S., Hu R., Wang Y., Quintana D., Zhang A., Huang Q., Lau J. (2015). Multiferroicity in doped hexagonal LuFeO_3_. Phys. Rev. B Condens. Matter Mater. Phys..

[50] Han H., Kim D., Chu K., Park J., Nam S.Y., Heo S., Yang C.H., Jang H.M. (2018). Enhanced Switchable Ferroelectric Photovoltaic Effects in Hexagonal Ferrite Thin Films via Strain Engineering. ACS Appl. Mater. Interfaces.

[51] Chen R., Wang J.F., Ouyang Z.W., He Z.Z., Wang S.M., Lin L., Liu J.M., Lu C.L., Liu Y., Dong C. (2018). Magnetic field induced ferroelectricity and half magnetization plateau in polycrystalline R_2_V_2_O_7_ (R = Ni, Co). Phys. Rev. B.

[52] Liu M., Lin L., Zhang Y., Li S., Huang Q., Garlea V.O., Zou T., Xie Y., Wang Y., Lu C. (2017). Cycloidal magnetism driven ferroelectricity in double tungstate LiFe(WO_4_)_2_. Phys. Rev. B.

[53] Terada N., Ikedo Y., Sato H., Khalyavin D.D., Manuel P., Miyake A., Matsuo A., Tokunaga M., Kindo K. (2017). Rich magnetoelectric phase diagrams of multiferroic single-crystal α-NaFeO_2_. Phys. Rev. B.

[54] Liu Y.J., Wang J.F., He Z.Z., Lu C.L., Xia Z.C., Ouyang Z.W., Liu C.B., Chen R., Matsuo A., Kohama Y. (2018). Unusual magnetoelectric memory and polarization reversal in the kagome staircase compound Ni_3_V_2_O_8_. Phys. Rev. B.

[55] Ghara S., Suard E., Fauth F., Tran T.T., Halasyamani P.S., Iyo A., Rodríguez-Carvajal J., Sundaresan A. (2017). Ordered aeschynite-type polar magnets RFeWO_6_ (R = Dy, Eu, Tb, and Y): A new family of type-II multiferroics. Phys. Rev. B.

[56] Zhou L., Dai J., Chai Y., Zhang H., Dong S., Cao H., Calder S., Yin Y., Wang X., Shen X. (2017). Realization of Large Electric Polarization and Strong Magnetoelectric Coupling in BiMn_3_Cr_4_O_12_. Adv. Mater..

[57] Wang X., Chai Y., Zhou L., Cao H., Dela Cruz C., Yang J., Dai J., Yin Y., Yuan Z., Zhang S. (2015). Observation of Magnetoelectric Multiferroicity in a Cubic Perovskite System: LaMn_3_Cr_4_O_12_. Phys. Rev. Lett..

[58] Cong J., Zhai K., Chai Y., Shang D., Khalyavin D.D., Johnson R.D., Kozlenko D.P., Kichanov S.E., Abakumov A.M., Tsirlin A.A. (2018). Spin-induced multiferroicity in the binary perovskite manganite Mn_2_O_3_. Nat. Commun..

[59] Oh Y.S., Artyukhin S., Yang J.J., Zapf V., Kim J.W., Vanderbilt D., Cheong S.W. (2014). Non-hysteretic colossal magnetoelectricity in a collinear antiferromagnet. Nat. Commun..

[60] Cai G.H., Greenblatt M., Li M.R. (2017). Polar Magnets in Double Corundum Oxides. Chem. Mater..

[61] Khanh N.D., Abe N., Sagayama H., Nakao A., Hanashima T., Kiyanagi R., Tokunaga Y., Arima T. (2016). Magnetoelectric coupling in the honeycomb antiferromagnet Co_4_Nb_2_O_9_. Phys. Rev. B.

[62] Wang Y., Pascut G.L., Gao B., Tyson T.A., Haule K., Kiryukhin V., Cheong S.W. (2015). Unveiling hidden ferrimagnetism and giant magnetoelectricity in polar magnet Fe_2_Mo_3_O_8_. Sci. Rep..

[63] Kurumaji T., Ishiwata S., Tokura Y. (2015). Doping-tunable ferrimagnetic phase with large linear magnetoelectric effect in a polar magnet Fe_2_Mo_3_O_8_. Phys. Rev. X.

[64] Li M.R., McCabe E.E., Stephens P.W., Croft M., Collins L., Kalinin S.V., Deng Z., Retuerto M., Gupta A.S., Padmanabhan H. (2017). Magnetostriction-polarization coupling in multiferroic M_n2_MnWO_6_. Nat. Commun..

[65] Terada N., Khalyavin D.D., Manuel P., Yi W., Suzuki H.S., Tsujii N., Imanaka Y., Belik A.A. (2015). Ferroelectricity induced by ferriaxial crystal rotation and spin helicity in a B -site-ordered double-perovskite multiferroic In_2_NiMnO_6_. Phys. Rev. B Condens. Matter Mater. Phys..

[66] Baum M., Komarek A.C., Holbein S., Fernández-Díaz M.T., André G., Hiess A., Sidis Y., Steffens P., Becker P., Bohatý L. (2015). Magnetic structure and multiferroic coupling in pyroxene NaFeSi_2_O_6_. Phys. Rev. B Condens. Matter Mater. Phys..

[67] Dong S., Liu J.M., Dagotto E. (2014). BaFe_2_Se_3_: A high TC magnetic multiferroic with large ferrielectric polarization. Phys. Rev. Lett..

[68] Zhang Y., Lin L., Zhang J.J., Huang X., An M., Dong S. (2017). Exchange striction driven magnetodielectric effect and potential photovoltaic effect in polar CaOFeS. Phys. Rev. Mater..

[69] De C., Ter-Oganessian N.V., Sundaresan A. (2018). Spin-driven ferroelectricity and large magnetoelectric effect in monoclinic MnSb_2_S_4_. Phys. Rev. B.

[70] Miyake A., Shibuya J., Akaki M., Tanaka H., Tokunaga M. (2015). Magnetic field induced polar phase in the chiral magnet CsCuCl_3_. Phys. Rev. B Condens. Matter Mater. Phys..

[71] Wu H.C., Chandrasekhar K.D., Yuan J.K., Huang J.R., Lin J.Y., Berger H., Yang H.D. (2017). Anisotropic spin-flip-induced multiferroic behavior in kagome Cu_3_Bi(SeO_3_)_2_O_2_Cl. Phys. Rev. B.

[72] Zhao L., Hung T.L., Li C.C., Chen Y.Y., Wu M.K., Kremer R.K., Banks M.G., Simon A., Whangbo M.H., Lee C. (2012). CuBr_2_-A New Multiferroic Material with High Critical Temperature. Adv. Mater..

[73] Polat O., Coskun M., Coskun F.M., Durmus Z., Caglar M., Turut A. (2018). Os doped YMnO_3_ multiferroic: A study investigating the electrical properties through tuning the doping level. J. Alloys Compd..

[74] Delaney K.T., Mostovoy M., Spaldin N.A. (2009). Superexchange-driven magnetoelectricity in magnetic vortices. Phys. Rev. Lett..

[75] Kimura T., Kawamoto S., Yamada I., Azuma M., Takano M., Tokura Y. (2003). Magnetocapacitance effect in multiferroic BiMnO_3_. Phys. Rev. B Condens. Matter Mater. Phys..

[76] Akshayveer A., Buroni F.C., Melnik R., Rodriguez-Tembleque L., Saez A. (2025). Environment-friendly technologies with lead-free piezoelectric materials: A review of recent developments, applications, and modelling approaches. arXiv.

[77] Bell A.J., Deubzer O.J., Bell A., Deubzer O. (2018). Lead-free piezoelectrics-The environmental and regulatory issues. Mrs Bull..

[78] Lebeugle D., Colson D., Forget A., Viret M. (2007). Very large spontaneous electric polarization in BiFeO_3_ single crystals at room temperature and its evolution under cycling fields. Appl. Phys. Lett..

[79] Catalan G., Scott J.F. (2009). Physics and applications of bismuth ferrite. Adv. Mater..

[80] Lee N., Vecchini C., Choi Y.J., Chapon L.C., Bombardi A., Radaelli P.G., Cheong S.W. (2013). Giant tunability of ferroelectric polarization in GdMn_2_O_5_. Phys. Rev. Lett..

[81] Kumar M., Yadav K.L., Varma G.D. (2008). Large magnetization and weak polarization in sol–gel derived BiFeO_3_ ceramics. Mater. Lett..

[82] Yin L.H., Zhao B.C., Fang J., Zhang R.R., Tang X.W., Song W.H., Dai J.M., Sun Y.P. (2012). Improved leakage current and ferromagnetic properties in magnetic field annealed BiFeO_3_-based ceramics. J. Solid State Chem..

[83] Lebeugle D., Colson D., Forget A., Viret M., Bonville P., Marucco J.F., Fusil S. (2007). Room-temperature coexistence of large electric polarization and magnetic order in BiFeO_3_ single crystals. Phys. Rev. B.

[84] Tu C.S., Chen C.S., Chen P.Y., Wei H.H., Schmidt V.H., Lin C.Y., Anthoniappen J., Lee J.M. (2016). Enhanced photovoltaic effects in A-site samarium doped BiFeO_3_ ceramics: The roles of domain structure and electronic state. J. Eur. Ceram. Soc..

[85] Luo J., Wang L., Sun Z., Liu P., Lai Y., Zhu L., Guo H. (2021). Novel bismuth ferrite nanopowder prepared by polyethylene glycol-assisted two-step solid-state reaction: Synthesis and magneto-optical properties. Ceram. Int..

[86] Liu L., Zhu X.L., Chen X.M. (2024). Improved ferroelectricity and magnetoelectric coupling effect of multielements co-substituted BiFeO_3_ ceramics. J. Am. Ceram. Soc..

[87] Kharbanda S., Dhanda N., Sun A.C.A., Thakur A., Thakur P. (2023). Multiferroic perovskite bismuth ferrite nanostructures: A review on synthesis and applications. J. Magn. Magn. Mater..

[88] Priya A.S., Henry J., Indhumathi R., Brindha E., Aepuru R. (2025). A comprehensive review on the effect of synthesis, doping, and composites on bismuth ferrite properties and applications. Ceram. Int..

[89] Michel C., Moreau J.M., Achenbach G.D., Gerson R., James W.J. (1969). The atomic structure of BiFeO_3_. Solid State Commun..

[90] Moreau J.M., Michel C., Gerson R., James W.J. (1971). Ferroelectric BiFeO_3_ X-ray and neutron diffraction study. J. Phys. Chem. Solids.

[91] Neaton J.B., Ederer C., Waghmare U.V., Spaldin N.A., Rabe K.M. (2005). First-principles study of spontaneous polarization in multiferroic BiFeO_3_. Phys. Rev. B Condens. Matter Mater. Phys..

[92] Glazer A.M. (1972). The classification of tilted octahedra in perovskites. Acta Crystallogr. Sect. B Struct. Crystallogr. Cryst. Chem..

[93] Ederer C., Spaldin N.A. (2005). Weak ferromagnetism and magnetoelectric coupling in bismuth ferrite. Phys. Rev. B.

[94] Cai S. (2013). Bismuth-Containing Multiferroics; Synthesis, Structure and Magnetic Properties. Master’s Thesis.

[95] Ismailzade I.G. (1966). X-Ray Diffraction Study of Phase Transitions in Bismuth Ferrite. Dokl. Akad. Nauk SSSR.

[96] Singh U., Kashyap A., Kainthla I., Shandilya M., Vernardou D., Le T.K., Kumar S. (2026). The role of space group symmetry in BiFeO_3_ for multifunctional energy harvesting applications. J. Electroceramics.

[97] Bernardo M.S. (2014). Synthesis, microstructure and properties of BiFeO_3_-based multiferroic materials: A review. Bol. Soc. Esp. Ceram. Vidr..

[98] Selbach S.M., Einarsrud M.A., Tybell T., Grande T. (2007). Synthesis of BiFeO_3_ by Wet Chemical Methods. J. Am. Ceram. Soc..

[99] Thomas N.W., Beitollahi A. (1994). Inter-relationship of octahedral geometry, polyhedral volume ratio and ferroelectric properties in rhombohedral perovskites. Acta Crystallogr. Sect. B.

[100] Guo R., Fang L., Dong W., Zheng F., Shen M. (2010). Enhanced photocatalytic activity and ferromagnetism in Gd doped BiFeO_3_ nanoparticles. J. Phys. Chem. C.

[101] Bucci J.D., Robertson B.K., James W.J. (1972). The precision determination of the lattice parameters and the coefficients of thermal expansion of BiFeO_3_. J. Appl. Crystallogr..

[102] Abe K., Sakai N., Takahashi J., Itoh H., Adachi N., Ota T. (2010). Leakage Current Properties of Cation-Substituted BiFeO_3_ Ceramics. Jpn. J. Appl. Phys..

[103] Kamba S., Nuzhnyy D., Savinov M., Šebek J., Petzelt J., Prokleška J., Haumont R., Kreisel J. (2007). Infrared and terahertz studies of polar phonons and magnetodielectric effect in multiferroic BiFeO_3_ ceramics. Phys. Rev. B.

[104] Redfern S.A.T., Wang C., Hong J.W., Catalan G., Scott J.F. (2008). Elastic and electrical anomalies at low-temperature phase transitions inBiFeO3. J. Phys. Condens. Matter.

[105] Lu J., Schmidt M., Lunkenheimer P., Pimenov A., Mukhin A.A., Travkin V.D., Loidl A. (2010). Magnetic susceptibility, phonons and dielectric constant of single crystalline BiFeO_3_. J. Phys. Conf. Ser..

[106] Zhang H., Jo W., Wang K., Webber K.G. (2014). Compositional dependence of dielectric and ferroelectric properties in BiFeO_3_–BaTiO_3_ solid solutions. Ceram. Int..

[107] Monteduro A.G., Rizzato S., Leo C., Karmakar S., Sirsi F., Leo A., Tasco V., Esposito M., Passaseo A., Caricato A.P. (2019). Dielectric and Ferroelectric Response of Multiphase Bi-Fe-O Ceramics. Phys. Status Solidi A.

[108] Palkar V.R., John J., Pinto R. (2002). Observation of saturated polarization and dielectric anomaly in magnetoelectric BiFeO_3_ thin films. Appl. Phys. Lett..

[109] Huyan H., Li L., Addiego C., Gao W., Pan X. (2019). Structures and electronic properties of domain walls in BiFeO3 thin films. Natl. Sci. Rev..

[110] Streiffer S.K., Parker C.B., Romanov A.E., Lefevre M.J., Zhao L., Speck J.S., Pompe W., Foster C.M., Bai G.R. (1998). Domain patterns in epitaxial rhombohedral ferroelectric films. I. Geometry and experiments. J. Appl. Phys..

[111] Teague J.R., Gerson R., James W.J. (1970). Dielectric hysteresis in single crystal BiFeO_3_. Solid State Commun..

[112] Ederer C., Spaldin N.A. (2005). Effect of Epitaxial Strain on the Spontaneous Polarization of Thin Film Ferroelectrics. Phys. Rev. Lett..

[113] Ravindran P., Vidya R., Kjekshus A., Fjellvåg H., Eriksson O. (2006). Theoretical investigation of magnetoelectric behavior in BiFeO_3_. Phys. Rev. B.

[114] Shvartsman V.V., Kleemann W., Haumont R., Kreisel J. (2007). Large bulk polarization and regular domain structure in ceramic BiFeO_3_. Appl. Phys. Lett..

[115] Lu J., Günther A., Schrettle F., Mayr F., Krohns S., Lunkenheimer P., Pimenov A., Travkin V.D., Mukhin A.A., Loidl A. (2010). On the room temperature multiferroic BiFeO_3_: Magnetic, dielectric and thermal properties. Eur. Phys. J. B.

[116] Cheng Z.X., Li A.H., Wang X.L., Dou S.X., Ozawa K., Kimura H., Zhang S.J., Shrout T.R. (2008). Structure, ferroelectric properties, and magnetic properties of the La-doped bismuth ferrite. J. Appl. Phys..

[117] Long S., Liu L., Wang Z., Zhang X. (2025). Enhanced ferroelectric and magnetoelectric properties of BiFeO_3_-based ceramics modified by SrTiO_3_. Ceram. Int..

[118] Pradhan S.K. (2013). Influence of iron deficiency on electric and magnetic behavior of Ho doped BiFeO_3_ electroceramic. J. Mater. Sci. Mater. Electron..

[119] Sosnowska I., Neumaier T.P., Steichele E. (1982). Spiral magnetic ordering in bismuth ferrite. J. Phys. C Solid State Phys..

[120] Ruette B., Zvyagin S., Pyatakov A.P., Bush A., Li J.F., Belotelov V.I., Zvezdin A.K., Viehland D. (2004). Magnetic-field-induced phase transition in BiFeO_3_ observed by high-field electron spin resonance: Cycloidal to homogeneous spin order. Phys. Rev. B.

[121] Sosnowska I., Przeniosło R. (2011). Low-temperature evolution of the modulated magnetic structure in the ferroelectric antiferromagnet BiFeO_3_. Phys. Rev. B Condens. Matter Mater. Phys..

[122] Ramazanoglu M., Ratcliff W., Choi Y.J., Lee S., Cheong S.-W., Kiryukhin V. (2011). Temperature-dependent properties of the magnetic order in single-crystal BiFeO_3_, Temperature-dependent properties of the magnetic order in single-crystal BiFeO_3_. Phys. Rev. B—Condens. Matter Mater. Phys..

[123] Przenioslo R., Regulski M., Sosnowska I. (2006). Modulation in Multiferroic BiFeO_3_: Cycloidal, Elliptical or SDW?. J. Phys. Soc. Jpn..

[124] Ramazanoglu M., Laver M., Ratcliff W., Watson S.M., Chen W.C., Jackson A., Kothapalli K., Lee S., Cheong S.W., Kiryukhin V. (2011). Local Weak Ferromagnetism in Single-Crystalline Ferroelectric BiFeO_3_. Phys. Rev. Lett..

[125] Chen P., Günaydın-Şen Ö., Ren W.J., Qin Z., Brinzari T.V., McGill S., Cheong S.-W., Musfeldt J.L. (2012). Spin cycloid quenching in Nd^3+^-substituted BiFeO_3_. Phys. Rev. B.

[126] Fischer P., Polomska M., Sosnowska I., Szymanski M. (1980). Temperature dependence of the crystal and magnetic structures of BiFeO_3_. J. Phys. C Solid State Phys..

[127] Xu T., Kan Y., Jin Y., Lu X., Wu H., He J., Lu X., Zhu W., Huang F., Zhu J. (2015). Effect of substrates on magnetization of BiFeO_3_ films. J. Appl. Phys..

[128] Liang X., Chen H., Sun N.X. (2021). Magnetoelectric materials and devices. APL Mater..

[129] Kadomtseva A.M., Zvezdin A.K., Popov Y.F., Pyatakov A.P., Vorob’ev G.P. (2004). Space-time parity violation and magnetoelectric interactions in antiferromagnets. J. Exp. Theor. Phys. Lett..

[130] Mostovoy M. (2006). Ferroelectricity in Spiral Magnets. Phys. Rev. Lett..

[131] Cheong S.-W., Mostovoy M. (2007). Multiferroics: A magnetic twist for ferroelectricity. Nat. Mater..

[132] Masashi T., Masaki A., Yuichi S. (2010). High-Field Study of Strong Magnetoelectric Coupling in Single-Domain Crystals of BiFeO_3_. J. Phys. Soc. Jpn..

[133] Ederer C., Fennie C.J. (2008). Electric-field switchable magnetization via the Dzyaloshinskii–Moriya interaction: FeTiO_3_ versus BiFeO_3_. J. Phys. Condens. Matter.

[134] Kawachi S., Miyake A., Ito T., Dissanayake S.E., Matsuda M., Ratcliff W., Xu Z., Zhao Y., Miyahara S., Furukawa N. (2017). Successive field-induced transitions in BiFeO_3_ around room temperature. Phys. Rev. Mater..

[135] Liu L., Li Q.T., Long S., Chen X.M. (2026). Structural evolution and improved magnetoelectric coupling effect in BiFeO_3_ ceramics based on the multi-element co-substitution. J. Magn. Magn. Mater..

[136] Naik V.B., Mahendiran R. (2009). Magnetic and magnetoelectric studies in pure and cation doped BiFeO_3_. Solid State Commun..

[137] Marzouki A., Harzali H., Loyau V., Gemeiner P., Zehani K., Dkhil B., Bessais L., Megriche A. (2018). Large magnetoelectric response and its origin in bulk Co-doped BiFeO_3_ synthesized by a stirred hydrothermal process. Acta Mater..

[138] Kolte J., Daryapurkar A.S., Agarwal M., Gulwade D.D., Gopalan P. (2017). Magnetoelectric properties of microwave sintered BiFeO_3_ and Bi_0.90_La_0.10_Fe_0.95_Mn_0.05_O_3_ nanoceramics. Mater. Chem. Phys..

[139] Wang N., Cheng J., Pyatakov A., Zvezdin A.K., Li J.F., Cross L.E., Viehland D. (2005). Multiferroic properties of modified BiFeO_3_-PbTiO_3_-based ceramics: Random-field induced release of latent magnetization and polarization. Phys. Rev. B—Condens. Matter Mater. Phys..

[140] Lee D., Kim M.G., Ryu S., Jang H.M., Lee S.G. (2005). Epitaxially grown La-modified BiFeO_3_ magnetoferroelectric thin films. Appl. Phys. Lett..

[141] Béa H., Bibes M., Barthélémy A., Bouzehouane K., Jacquet E., Khodan A., Contour J.-P., Fusil S., Wyczisk F., Forget A. (2005). Influence of parasitic phases on the properties of BiFeO_3_ epitaxial thin films. Appl. Phys. Lett..

[142] Jartych E., Pikula T., Kowal K., Dzik J., Guzdek P., Czekaj D. (2016). Magnetoelectric Effect in Ceramics Based on Bismuth Ferrite. Nanoscale Res. Lett..

[143] Ding Y., Wang T.H., Yang W.C., Lin T.C., Tu C.S., Yao Y.D., Wu K.T. (2011). Magnetization, magnetoelectric effect, and structure transition in BiFeO_3_ and Bi_0.95_La_0.05_)FeO_3_ multiferroic ceramics. IEEE Trans. Magn..

[144] Rai R., Mishra S.K., Singh N.K., Sharma S., Kholkin A.L. (2011). Preparation, structures, and multiferroic properties of single-phase BiRFeO_3_, R = la and Er ceramics. Curr. Appl. Phys..

[145] Bhushan B., Wang Z., van Tol J., Dalal N.S., Basumallick A., Vasanthacharya N.Y., Kumar S., Das D. (2012). Tailoring the magnetic and optical characteristics of nanocrystalline BiFeO_3_ by Ce doping. J. Am. Ceram. Soc..

[146] Dai H., Li T., Chen Z., Liu D., Xue R., Zhao C., Liu H., Huang N. (2016). Studies on the structural, electrical and magnetic properties of Ce-doped BiFeO3ceramics. J. Alloys Compd..

[147] Rhaman M.M., Matin M.A., Hakim M.A., Islam M.F. (2019). Dielectric, ferroelectric and ferromagnetic properties of samarium doped multiferroic bismuth ferrite. Mater. Res. Express.

[148] Nalwa K.S., Garg A. (2008). Phase evolution, magnetic and electrical properties in Sm-doped bismuth ferrite. J. Appl. Phys..

[149] Vashisth B.K., Bangruwa J.S., Gairola S.P., Verma V. (2018). Structural, dielectric, ferroelectric and magnetic properties of Gd doped BiFeO_3_. Integr. Ferroelectr..

[150] Chen J., Xu B., Liu X.Q., Gao T.T., Bellaiche L., Chen X.M. (2019). Symmetry Modulation and Enhanced Multiferroic Characteristics in Bi_1-x_Nd_x_FeO_3_ Ceramics. Adv. Funct. Mater..

[151] Remya K.P., Amirthapandian S., Manivel Raja M., Viswanathan C., Ponpandian N. (2016). Effect of Yb substitution on room temperature magnetic and dielectric properties of bismuth ferrite nanoparticles. J. Appl. Phys..

[152] Ozdilek C., Ozenbas M. (2020). Hydrothermal synthesis of Yb-doped BiFeO_3_ crystallites and their structural, magnetic and electrical properties. Ceram. Int..

[153] Yan Z., Wang K.F., Qu J.F., Wang Y., Song Z.T., Feng S.L. (2007). Processing and properties of Yb-doped BiFeO_3_ ceramics. Appl. Phys. Lett..

[154] Cyriac J., John J.C., Kalarikkal N., Augustine S. (2019). Tailoring the dielectric and magnetic properties of Eu-substituted BiFeO_3_ nanoparticles. Mater. Today Proc..

[155] Song G.L., Ma G.J., Su J., Wang T.X., Yang H.Y., Chang F.G. (2014). Effect of Ho^3+^ doping on the electric, dielectric, ferromagnetic properties and TC of BiFeO_3_ ceramics. Ceram. Int..

[156] Das R., Mandal K. (2012). Magnetic, ferroelectric and magnetoelectric properties of Ba-doped BiFeO_3_. J. Magn. Magn. Mater..

[157] Naushin N., Md Shahriar S., Roy O., Sharif A. (2020). Modification in Structural, Morphological, Magnetic and Ferroelectric Properties of Calcium-Doped Bismuth Ferrite Nanoparticles. J. Eng. Sci..

[158] Verma K.C., Kotnala R.K. (2016). Tailoring the multiferroic behavior in BiFeO_3_ nanostructures by Pb doping. RSC Adv..

[159] Maleki H., Zakeri M., Fathi R. (2018). Experimental study of the effect of yttrium on the structural, thermal, and magnetic properties of BiFeO_3_. Appl. Phys. A.

[160] Aziz S., Monteduro A.G., Rawat R., Rizzato S., Leo A., Khalid S., Maruccio G. (2024). Preparation and Characterization of BXFO High-Entropy Oxides. Magnetochemistry.

[161] Sharma A.D., Sharma H.B. (2019). Electrical and Magnetic Properties of Mn-Doped BiFeO_3_ Nanomaterials. Integr. Ferroelectr..

[162] Kumar M., Yadav K.L. (2006). Study of room temperature magnetoelectric coupling in Ti substituted bismuth ferrite system. J. Appl. Phys..

[163] Bari V., de Maria B., Mazzucco C.E. (2008). Effects of ion doping at different sites on electrical properties of multiferroic BiFeO_3_ ceramics. J. Phys. D. Appl. Phys. To.

[164] Jun Y.K., Lee S.B., Kim M., Hong S.H., Kim J.W., Kim K.H. (2007). Dielectric and magnetic properties in Ta-substituted BiFeO_3_ ceramics. J. Mater. Res..

[165] Gholam T., Ablat A., Mamat M., Wu R., Aimidula A., Bake M.A., Zheng L., Wang J., Qian H., Wu R. (2017). Local electronic structure analysis of Zn-doped BiFeO_3_ powders by X-ray absorption fine structure spectroscopy. J. Alloys Compd..

[166] Wei J., Haumont R., Jarrier R. (2010). Nonmagnetic Fe-site doping of BiFeO_3_ multiferroic ceramics. Appl. Phys. Lett..

[167] Thansanga L., Shukla A., Kumar N., Choudhary R.N.P. (2020). Study of effect of Dy substitution on structural, dielectric, impedance and magnetic properties of bismuth ferrite. J. Mater. Sci. Mater. Electron..

[168] Kubota M., Oka K., Yabuta H., Miura K., Azuma M. (2013). Structure and Magnetic Properties of BiFe_1–x_CoxO_3_ and Bi_0.9_Sm_0.1_Fe_1–x_CoxO_3_. Inorg. Chem..

[169] Yoo Y.J., Hwang J.S., Lee Y.P., Park J.S., Kang J.H., Kim J., Lee B.W., Seo M.S. (2013). High ferromagnetic transition temperature in multiferroic BiFe _0.95_Ni_0.05_O_3_ compound. J. Appl. Phys..

[170] Deng X.L., Cai W., Fu C.L., Zhang Y.Q., Gao R.L., Chen G., Huang J.M. (2015). Effect of Mn Substitution on Microstructures, Dielectric and Magnetic Properties of BiFeO_3_ Ceramics. Mater. Sci. Forum.

[171] Pandit P., Satapathy S., Sharma P., Gupta P.K., Yusuf S.M., Sathe V.G. (2011). Structural, dielectric and multiferroic properties of Er and La substituted BiFeO_3_ ceramics. Bull. Mater. Sci..

[172] Islam M.R., Islam M.S., Zubair M.A., Usama H.M., Azam M.S., Sharif A. (2018). Evidence of superparamagnetism and improved electrical properties in Ba and Ta co-doped BiFeO_3_ ceramics. J. Alloys Compd..

[173] Srivastava A., Singh A.K., Srivastava O.N., Tewari H.S., Masood K.B., Singh J. (2020). Magnetic and Dielectric Properties of La and Ni Co-substituted BiFeO_3_ Nanoceramics. Front. Phys..

[174] Troyanchuk I.O., Tereshko N.V., Karpinsky D.V., Kholkin A.L., Kopcewicz M., Bärner K. (2011). Enhanced piezoelectric and magnetic properties of Bi_1−x_Ca_x_Fe_1−x/2_Nb_x/2_O_3_ solid solutions. J. Appl. Phys..

[175] Lv J., Wu J., Wu W. (2015). Enhanced Electrical Properties of Quenched (1−x)Bi_1−y_Sm_y_FeO_3_−xBiScO_3_ Lead-Free Ceramics. J. Phys. Chem. C.

[176] Ratkovski D.R., Ribeiro P.R.T., Machado F.L.A., Banerjee P., Franco A. (2018). On the magnetic properties of the multiferroic ceramics Bi_0.99_Y_0.01_Fe_1−x_Ni_x_O_3_ (0.01 ≤ x ≤ 0.05). J. Magn. Magn. Mater..

[177] Shu H., Ma Y., Wang Z., Mao W., Chu L., Yang J., Wu Q., Min Y., Song R., Li A. (2017). Structural, Optical and Multiferroic Properties of (Nd, Zn)-Co-doped BiFeO_3_ Nanoparticles. J. Supercond. Nov. Magn..

[178] ShouYu W., Yu F., WeiFang L., DaShu Y., DeJun L. (2013). Effects of Co doping on electronic structure and electric/magnetic properties of La_0.1_Bi_0.9_FeO_3_ ceramics. Sci. Chi-Na-Phys. Mech. Astron.

[179] Chaudhari Y.A., Singh A., Mahajan C.M., Jagtap P.P., Abuassaj E.M., Chatterjee R., Bendre S.T. (2013). Multiferroic properties in Zn and Ni co-doped BiFeO_3_ ceramics by solution combustion method (SCM). J. Magn. Magn. Mater..

[180] Hu Z., Li M., Yu Y., Liu J., Pei L., Wang J., Liu X., Yu B., Zhao X. (2010). Effects of Nd and high-valence Mn co-doping on the electrical and magnetic properties of multiferroic BiFeO_3_ ceramics. Solid State Commun..

[181] Pradhan D.K., Tripathy S.N., Mishra K.K., Sen S., Mishra B.G., Pradhan D.K., Palai R. (2013). Phase transition and magneto-electric coupling of BiFeO_3_–YMnO_3_ multiferroic nanoceramics. J. Appl. Phys..

[182] Pillai S., Bhuwal D., Banerjee A., Shelke V. (2013). Bulk interface engineering for enhanced magnetization in multiferroic BiFeO_3_ compounds. Appl. Phys. Lett..

[183] Pillai S., Tripathi D., Ahmad Para T., Das A., Shripathi T., Shelke V. (2016). Confirmation of enhanced magnetic moment in interface-engineered BiFeO_3_-LaMnO_3_ composites. J. Appl. Phys..

[184] Rhaman M.M., Matin M.A., Hossain M.N., Khan M.N.I., Hakim M.A., Islam M.F. (2020). Ferromagnetic, electric, and ferroelectric properties of samarium and cobalt co-doped bismuth ferrite nanoparticles. J. Phys. Chem. Solids.

[185] Gu Y.-H., Liu Y., Yao C., Ma Y.-W., Wang Y., Chan H.L.-W., Chen W.-P. (2014). Ho and Ti co-doped BiFeO_3_ multiferroic ceramics with enhanced magnetization and ultrahigh electrical resistivity. Chin. Phys. B.

[186] Dai H., Xue R., Chen Z., Li T., Chen J., Xiang H. (2014). Effect of Eu, Ti co-doping on the structural and multiferroic properties of BiFeO_3_ ceramics. Ceram. Int..

[187] Wang T., Ye H., Wang X., Cui Y., Mei H., Song S., Zhao Z., Wang M., Rosaiah P., Ma Q. (2025). Improved Ferroelectric and Magnetic Properties of Bismuth Ferrite-Based Ceramics by Introduction of Non-Isovalent Ions and Grain Engineering. Nanomaterials.

[188] Zhu J.H., Dai J.Q., Xu J.W., Li X.Y. (2018). Effect of Zn and Ti Co-doping on structure and electrical properties of BiFeO_3_ ceramics. Ceram. Int..

[189] Satyanarayana S., Sarma S.C., Peter S.C., Bhattacharya S. (2019). Magnetic characterization of nano-sized terbium doped bismuth ferrite synthesized by sol-gel method. J. Magn. Magn. Mater..

[190] Notonegoro H.A., Soegijono B., Budianto E., Yogatama A. (2019). Structural, non-volatile magnetization, and dielectric studies on zinc-doped BiFeO_3_. J. Phys. Conf. Ser..

[191] Dhahri J., Boudard M., Zemni S., Roussel H., Oumezzine M. (2008). Structure and magnetic properties of potassium doped bismuth ferrite. J. Solid State Chem..

[192] Ahmed M.A., Mansour S.F., El-Dek S.I., Abu-Abdeen M. (2014). Conduction and magnetization improvement of BiFeO_3_ multiferroic nanoparticles by Ag+ doping. Mater. Res. Bull..

[193] Mishra M.K., Mahaling R.N. (2017). Mg doping in BiFeO_3_: An advantage over pure BiFeO3 having enhanced ferroelectric and optical properties for opto-electronic device applications. Ferroelectrics.

[194] Chowdhury S.S., Kamal A.H.M., Hossain R., Hasan M., Islam M.F., Ahmmad B., Basith M.A. (2017). Dy doped BiFeO_3_: A bulk ceramic with improved multiferroic properties compared to nano counterparts. Ceram. Int..

[195] Zhang H., Liu W., Wu P., Hai X., Guo M., Xi X., Gao J., Wang X., Guo F., Xu X. (2014). Novel behaviors of multiferroic properties in Na-Doped BiFeO_3_ nanoparticles. Nanoscale.

[196] Uniyal P., Yadav K.L. (2009). Pr doped bismuth ferrite ceramics with enhanced multiferroic properties. J. Phys. Condens. Matter.

[197] Taryana E., Soegijono B., Notonegoro H. (2021). The Influence of Li-doping on the Microwave Absorption Properties of BiFeO_3_ Nanoparticles. J. Surf. Sci. Nanotechnol..

[198] Anjitha V.K., Athulya M., Adarsh V.S., Vinod Kumar R. (2025). Investigation of Structural, Optical, and Magnetic Properties of Molybdenum Substituted Bismuth Ferrite Nanoparticles. ChemistrySelect.

[199] Chauhan S., Kumar M., Pal P. (2016). Substitution driven structural and magnetic properties and evidence of spin phonon coupling in Sr-doped BiFeO_3_ nanoparticles. RSC Adv..

[200] Kiani M., Tian X.Q., Kiani A.B., Rehman S.U., Khan S.A., Khan K., Tareen A.K., Khan Q.U., Mahmood I. (2021). A first principle study: Effect of tin substitution on magnetic properties of bismuth ferrite nanoparticles prepared by sol-gel synthesis method. Inorg. Chem. Commun..

[201] Rizwan S., Umar M., Babar Z.U.D., Awan S.U., Rehman M.A.U. (2019). Selenium-enriched flower-like of bismuth ferrite nanosheets assembly with associated magnetic properties. AIP Adv..

[202] Bai P., Zeng Y., Han J., Wei Y., Li M. (2019). Influence of Al doping on structural, dielectric, and ferroelectric properties of multiferroic BiFeO_3_ ceramics. Ceram. Int..

[203] Wang Y., Zhang X. (2022). Cr doping effects on the structural and magnetic properties of BiFeO_3_ nanoparticles synthesized hydrothermal method. J. Magn. Magn. Mater..

[204] Najm A.A.A., Baqiah H., Shaari A.H., Kechik M.M.A., Kien C.S., Zahari R.M., Li Q. (2021). Investigation of structural, dielectric, impedance and magnetic properties of BiFe_1−x_In_x_O_3_ (0.0 ≤ x ≤ 0.6) ceramics. Results Phys..

[205] Sathiya Priya A., Geetha D. (2021). Impact of (Zr, Cu) Ion Substitution on the Optical, Dielectric, and Impedance Behavior of BiFeO_3_. Braz. J. Phys..

[206] Cui Y.F., Zhao Y.G., Luo L.B., Yang J.J., Chang H., Zhu M.H., Xie D., Ren T.L. (2010). Dielectric, magnetic, and magnetoelectric properties of La and Ti codoped BiFeO_3_. Appl. Phys. Lett..

[207] Abdelmadjid K., Lukacs V.A., Gheorghiu F. (2023). Dielectric and magnetic properties of (Pb, Ti) co-doped BiFeO_3_ multiferroic ceramics. Ceram. Int..

[208] Zhu S., Gu Y., Xiong Y., Zhou X., Liu Y., Wang Y., Chen W. (2016). Effects of Ba and Ti co-doping on BiFeO_3_ multiferroic ceramics optimized through two-step doping. J. Adv. Ceram..

[209] Zhou Z., Bai W., Liu N., Zhang W., Chen S., Wang P., Liu J., Zhai J., Guo J., Du G. (2025). Ultrahigh capacitive energy storage of BiFeO3-based ceramics through multi-oriented nanodomain construction. Nat. Commun..

[210] Chiang C.H., Wei C.C., Kuo P.C., Hung W.L., Lin Y.C., Wang L.S., Lung C.A., Liu T.R., Cyue Z.W., Shiue J. (2026). Single symmetric nanopores in ultrathin crystalline ferroelectric BiFeO_3_ as polarization-switchable ionic diodes. Nat. Commun..

